# Radiological predictors of shunt response in the diagnosis and treatment of idiopathic normal pressure hydrocephalus: a systematic review and meta-analysis

**DOI:** 10.1007/s00701-022-05402-8

**Published:** 2022-11-26

**Authors:** Santhosh G. Thavarajasingam, Mahmoud El-Khatib, Kalyan Vemulapalli, Hector A. Sinzinkayo Iradukunda, Sajeenth Vishnu K., Robin Borchert, Salvatore Russo, Per K. Eide

**Affiliations:** 1 Faculty of Medicine, Imperial College London, London, UK; 2 Department of Clinical Neurosciences, Cambridge University Hospital NHS Trust, Cambridge, UK; 3 Department of Neurosurgery, Imperial College Healthcare NHS Trust, London, UK; 4 Department of Neurosurgery, Oslo University Hospital – Rikshospitalet, Oslo, Norway; 5 Institute of Clinical Medicine, Faculty of Medicine, University of Oslo, Oslo, Norway

**Keywords:** Idiopathic normal pressure hydrocephalus, iNPH, Normal pressure hydrocephalus, NPH, Predict, Shunt response, Prediction, Radiological predictor, Radiological marker, DESH, Callosal angle, Evan's Index

## Abstract

**Background:**

Patients with the dementia subtype idiopathic normal pressure hydrocephalus (iNPH) may improve clinically following cerebrospinal fluid (CSF) diversion (shunt) surgery, though the predictors of shunt response remain debated. Currently, radiological features play an important role in the diagnosis of iNPH, but it is not well established which radiological markers most precisely predict shunt responsive iNPH.

**Objective:**

To conduct a systematic review and meta-analysis to identify radiological predictors of shunt responsiveness, evaluate their diagnostic effectiveness, and recommend the most predictive radiological features.

**Methods:**

Embase, MEDLINE, Scopus, PubMed, Google Scholar, and JSTOR were searched for original studies investigating radiological predictors of shunt response in iNPH patients. Included studies were assessed using the ROBINS-1 tool, and eligible studies were evaluated using a univariate meta-analysis.

**Results:**

Overall, 301 full-text papers were screened, of which 28 studies were included, and 26 different radiological features were identified, 5 of these met the inclusion criteria for the meta-analysis: disproportionately enlarged subarachnoid space (DESH), callosal angle, periventricular white matter changes, cerebral blood flow (CBF), and computerized tomography cisternography. The meta-analysis showed that only callosal angle and periventricular white matter changes significantly differentiated iNPH shunt responders from non-responders, though both markers had a low diagnostic odds ratio (DOR) of 1.88 and 1.01 respectively. None of the other radiological markers differentiated shunt responsive from shunt non-responsive iNPH.

**Conclusion:**

Callosal angle and periventricular changes are the only diagnostically effective radiological predictors of shunt responsive iNPH patients. However, due to the DORs approximating 1, they are insufficient as sole predictors and are advised to be used only in combination with other diagnostic tests of shunt response. Future research must evaluate the combined use of multiple radiological predictors, as it may yield beneficial additive effects that may allow for more robust radiological shunt response prediction.

**Supplementary Information:**

The online version contains supplementary material available at 10.1007/s00701-022-05402-8.

## Introduction

Normal pressure hydrocephalus (NPH) was first described by Hakim and Adams in 1965 [1] and classically presents with the clinical triad of dementia, urinary incontinence and ataxia, and imaging features of enlarged ventricles [19]. Idiopathic NPH (iNPH) is the most common form of adult-onset hydrocephalus, and the current gold standard for definitive diagnosis of iNPH is a positive clinical response to shunt surgery [40]. Despite marked clinical improvement in those who respond to shunting, depending on the criteria for shunting, clinical shunt response differs in the various series and even has been reported as 50% [15, 19, 30, 56]. Given the significant proportion of iNPH patients who do not benefit from shunting, as well as the complication risk of shunt surgery, it is pivotal pre-operatively to distinguish likely shunt responsive and shunt non-responsive iNPH [48].

Since fulfilling the diagnostic criteria of iNPH may not imply shunt response, various supplementary tests to predict treatment response have been used. A recent systematic review and meta-analysis disclosed that the supplementary tests most precisely predicting shunt response are intracranial pulse pressure monitoring, followed by extended lumbar drainage and thereafter infusion testing [54]. However, since invasive tests give higher costs and a higher risk profile, there is a need for less invasive predictive tests, e.g., biochemical and radiological ones, as recently outlined by Eide and Sorteberg [11]. In this regard, radiological markers stand out. Since iNPH was first described, radiological measures of ventriculomegaly have been essential for the diagnosis, as reflected by the current American-European and Japanese diagnostic iNPH guidelines [40, 48]. As such, the recently updated Japanese guidelines [38, 40] highlight the importance of the so-called disproportionately enlarged sub-arachnoid space hydrocephalus (DESH) in differentiating shunt responsive versus shunt non-responsive iNPH. In addition, the updated guidelines [40] recommend the use of Evan’s index (EI) and callosal angle (CA) as radiological predictors of shunt-responsive iNPH. It is, however, important to bear in mind that the updated guidelines [40] refer to a narrative review that lacks a systematic search strategy as well as a robust quantitative analysis. Hence, it is difficult to ascertain reliable sensitivities and specificities, as well as values for overall diagnostic accuracy.

Two systematic reviews [43, 44] with meta-analyses have been published recently evaluating Evan’s index, callosal angle, and DESH that both performed a quantitative analysis comparing diagnostic effectiveness of these radiological markers. Both reviews found that callosal angle outperformed Evan’s index as predictor of shunt responsiveness in terms of diagnostic accuracy and intra-observer agreement. However, these reviews share a significant methodological limitation that undermines the statistical and clinical significance of their findings: their inclusion criteria did not incorporate the salient point that a definitive NPH diagnosis must be defined by studies as a positive response to shunt surgery [40]. This would require a comparison between shunt responsive and shunt non-responsive iNPH. Instead, most studies included by both reviews used healthy subjects as a control group compared with those diagnosed with iNPH, which is not reflective of the clinical problem, namely distinguishing NPH shunt responders from non-responders. Therefore, both reviews may aid in distinguishing patients with iNPH from healthy patients without iNPH radiologically; however, the clinical relevance of this is limited.

Given the limitations of the existing literature, this present study aims to be the first meta-analysis to evaluate all radiological imaging markers used in the prediction of shunt responsiveness in iNPH patients.

## Methods

### Literature search

This systematic review was conducted following the Cochrane Collaboration guidelines [57] and Preferred Reporting Items for Systematic Reviews and Meta-Analyses (PRISMA) [37]. The completed PRISMA Checklist can be found in Supplementary Material: Table 1. This review was not registered. A comprehensive search of MEDLINE, Embase, Scopus, PubMed, Google Scholar, and JSTOR was conducted from January 1965 to September 2021 performed to answer the following research question: “Which radiological features predict shunt-responsive iNPH?” Normal-pressure hydrocephalus was first described in 1965 [1]. The search term used in all databases was “Normal Pressure Hydrocephalus.” The specific search string can be found in Supplementary Material: Table 2.

### Study inclusion and exclusion criteria

A table of the inclusion and exclusion criteria used in this review can be found in Supplementary Material: Table 3. In the first abstract screening, conducted by two reviewers, all original articles in the English language that reported on iNPH diagnosis were included. Subsequently, from this preliminary list, only studies reporting the use of radiological features for the prediction of shunt response in iNPH management, as well as those fulfilling our inclusion criteria, were included. Our inclusion criteria included the following: adult iNPH patients, radiological confirmation of hydrocephalus, 1 or more clinical features of iNPH, use of cerebrospinal fluid (CSF) shunt, objective system of functional grading of patients preoperatively, and a minimum of 3 months post-operatively, and that the radiological test was evaluated for the ability to predict SR.

### Eligibility assessment, data extraction, and quality assessment

Following the abstract screening, all included papers were assessed for eligibility by two independent reviewers. Any disagreements were resolved by consensus after discussion with a third and subsequently a fourth reviewer. All relevant data were extracted manually using the Covidence data collection tool [9]. Relevant data included author names, publication dates, number of shunted patients, study methodology (specific radiological methodology, cutoff specification, image specification, image plane), criteria for NPH diagnosis, criteria for shunt response, main reported outcomes (differences in radiological markers between shunt response and shunt non-responsive; area under curve, sensitivity and specificity of the radiological marker for predicting shunt-responsive iNPH, relevant statistical analyses including positive and negative predictive values), complications and drop-out rates, funding declarations, and conflicts of interests. No assumptions were made regarding any studies’ content. All articles were critically appraised, and risk of bias was determined against all the domains of the ROBINS-I [53] tool by two independent reviewers, and a consensus was reached by discussion with a third reviewer, shown in Supplementary Material: Table 4. An explanation for the risk of bias scoring was provided for those studies being scored as serious or critical overall bias in Supplementary Material: Table 4. Furthermore, the level of evidence for each included article was scored using the Oxford Centre of Evidence-Based Medicine (OCEBM) Levels of Evidence Table, shown in Supplementary Material: Table 5 [21].

### Statistical analysis

An Egger’s regression and asymmetry test [57] were used to assess publication bias (*p* < 0.05% = significant). Data preparation, statistical analysis, and plot synthesis were carried out by utilizing meta package with the R software (version 4.0.4) [47]. The R code is shown in Supplementary Material: Table 6. A random-effects sub-group meta-analysis was conducted for each radiological marker that had three or more studies evaluating its use and provided appropriate statistical data to allow for meta-analysis. Studies must have included the following information: sample size for shunt responsive and non-shunt responsive group and for each radiological marker; the diagnostic odds ratio and/or sensitivity and specificity and/or positive predictive value and negative predictive value and/or true positives, false positives, true negatives, and false negatives for the respective radiological marker in the context of SR prediction. These values were needed to calculate the treatment effect size for the respective radiological marker, namely the diagnostic odds ratio. If only two studies discussed a biomarker, then the biomarker was included in the albatross plot but not in the meta-analysis. The inverse variance method was used for pooling effect sizes [13]. The Hartung-Knapp [18] method was used to adjust test statistics and confidence intervals. The Restricted maximum-likelihood estimator was used to analyze variance between studies. The *t*-test was used to calculate the overall statistical result of each meta-analysis with the associated *p*-value. Heterogeneity was estimated using the chi-squared statistic (*I*) with the associated *p*-value. A statistical significance was assumed for *p* < 0.05. A sensitivity analysis was performed in two steps. Firstly, if included studies for each radiological marker included in the meta-analysis were rated at “serious” or “critical” overall risk of bias according to ROBINS-I tool [53], an additional sub-group random-effects meta-analysis without these studies was performed by utilizing meta package [17] with the R software (version 4.0.4) [47]. Secondly, a multivariate mixed-effects meta-regression model was built and calculated by utilizing meta package [17] with the R software (version 4.0.4) [47]. The following regression equation was employed:

$${\widehat{\theta }}_{k}= \theta + {\beta }_{1}{x}_{k }+{\epsilon }_{k }+{\zeta }_{k}$$

Reading the equation left to right, ${\widehat{\theta }}_{k}$ denotes the observed effect size of each study ($k$) and acts as the dependent variable. $\theta$ denotes the *y*-axis intercept, and ${\beta }_{1}{x}_{k}$ is the independent variable, an arm-level covariate vector. The variables ${\epsilon }_{k }$ and ${\zeta }_{k}$ denote two independent error variables. ${\zeta }_{k}$ explains that even the measured true effect size of each study is merely sampled from an overarching effect size distribution, which implies that heterogeneity variance exists between studies. The error term ${\epsilon }_{k}$ describes the underlying independent sampling error which causes the effect size of a study to deviate from the true effect size. In this study, the following explanatory variable model was chosen to explain and represent the error term ${\epsilon }_{k}$:

$${\epsilon }_{k }=\left({{\beta }_{{2}_{Sample}}+\beta }_{{3}_{Year}}{+ \beta }_{{4}_{Age}}+ {\beta }_{{5}_{Females}}+ {\beta }_{{6}_{HTN}} + {\beta }_{{7}_{Gait- }}+ {\beta }_{{8}_{mRS }}+ {\beta }_{{9}_{MMSE }}{+ \beta }_{{10}_{EI }}+{{+ \beta }_{{11}_{CA }}+ \beta }_{{12}_{Depression }}+ {\beta }_{{13}_{S-R }}{+ \beta }_{{14}_{Compl. }}{+ \beta }_{{15}_{Imaging plane }}{+ \beta }_{{16}_{Imaging modality}} \right){x}_{k}$$

The error term ${\epsilon }_{k}$ is hypothesized to be influenced by the sample size (${\beta }_{{2}_{Sample}})$, the year of publication (${\beta }_{{3}_{Year}})$, the mean age of the overall population sample (${\beta }_{{4}_{Age}})$, the proportion of females (${\beta }_{{5}_{Females}})$, with arterial hypertension (${\beta }_{{6}_{HTN}}$), with gait deficits (${\beta }_{{7}_{Gait-}}$), the mean MMSE score (${\beta }_{{9}_{MMSE}}$), the mean Evan’s index score (${\beta }_{{10}_{mRS}}$), the mean callosal angle (${\beta }_{{11}_{CA}}$), the proportion of patients with depression (${\beta }_{{12}_{Depression}}$), the proportion of shunt-responsive patients (${\beta }_{{13}_{S-R}}$), the proportion of patients experiencing complications (${\beta }_{{14}_{Compl.}}$), the imaging plane used (${\beta }_{{15}_{Imaging plane}}$), and the imaging modality used (${\beta }_{{16}_{Imaging modality}}$). The different explanatory variables were calculated singularly as sole covariates in separate meta-regressions, and if significant coefficients were yielded, further regression analyses were performed by adding additional covariates to the sole covariate to assess if significance was retained. Finally, an additional meta-analysis was subsequently performed by removing the studies that caused the significant covariates. The significant studies were identified by examining the bubble plots for outliers. The heatmap was generated using the R software (version 4.0.4) [47]. To produce a more robust and useful heatmap, the machine learning (ML) algorithm and package *MICE* [58], multivariate imputation by chained equations function, was employed to impute missing variables and obliterate variables with zero covariance that may skew the visualization. The correlation matrix was also generated using the R software (version 4.0.4) [47]. A non-imputed multivariate correlation matrix was generated first, given sufficient data for each parameter included. Then, to ensure validity of this correlation matrix, a second multivariate correlations matrix based on imputation by *MICE* [58] was generated for the same data, as well as a univariate scatterplot matrix to visualize pair-wise relationship of the correlation variables including regression line and a bivariate scatter plot of matrices (SPLOM) with locally estimated scatterplot smoothing line (LOASS) for bivariate correlation analysis with Pearson coefficient and histograms.

## Results

**Fig. 1:**
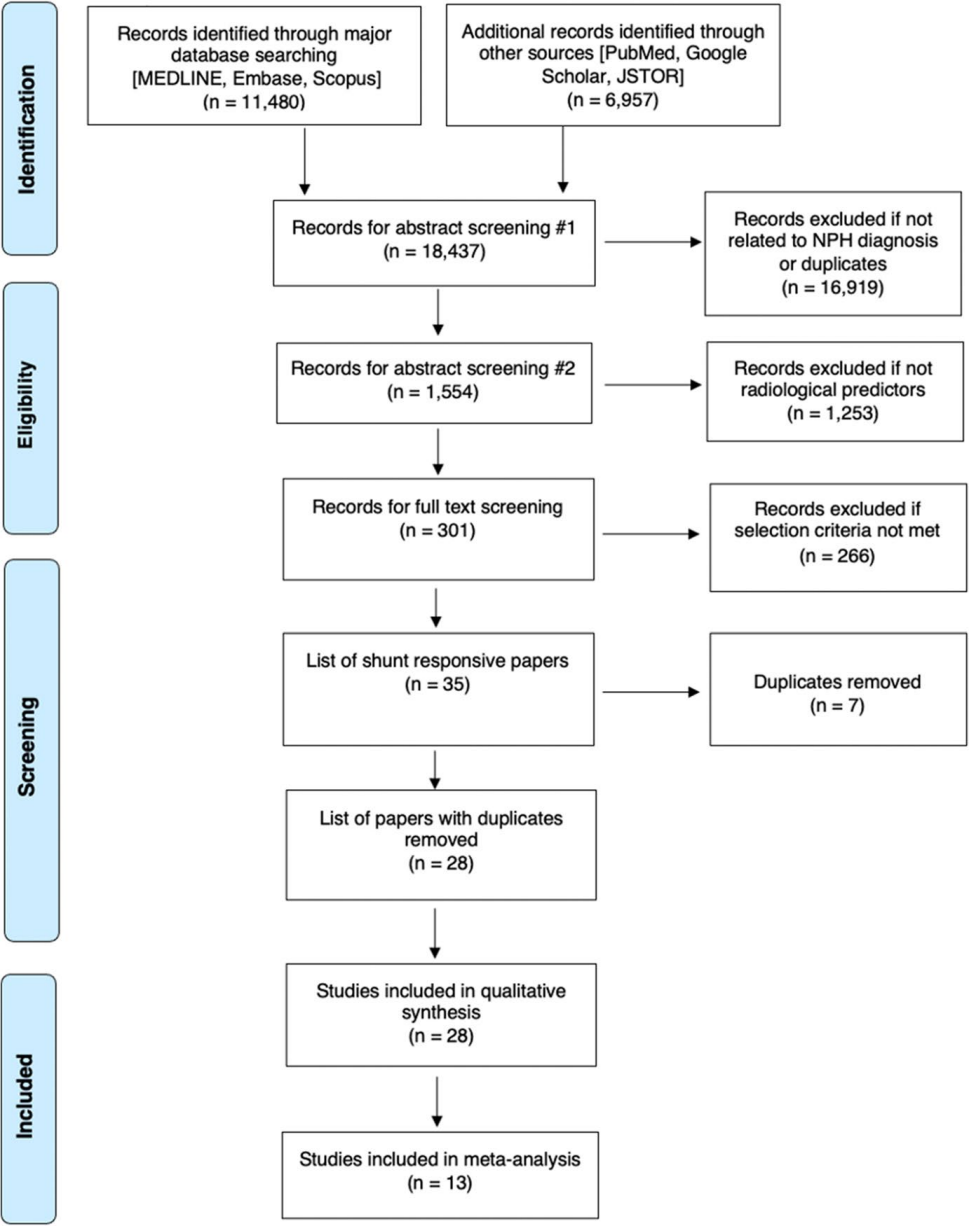
Preferred Reporting Items for Systematic Reviews and Meta-Analyses (PRISMA) flowchart outlining the study selection process

**Fig. 2:**
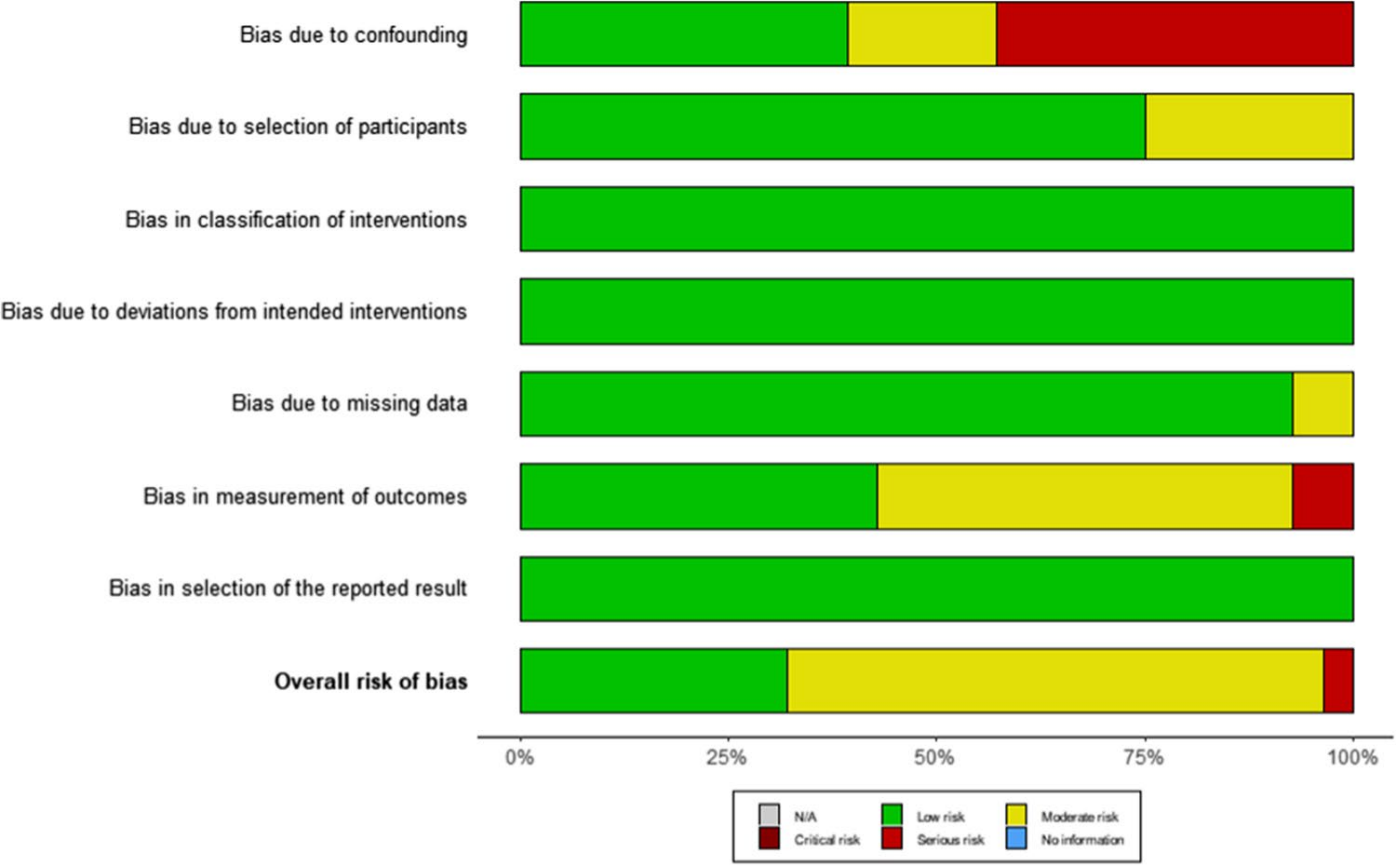
A risk of bias summary plot for non-randomized studies with bar chart of the distribution of risk-of-bias judgments for all included studies (*n* = 28) [2–5, 8, 14, 16, 20, 23, 25–27, 31, 33, 34, 39, 41, 42, 45–47, 49, 59–64] cross the domains of the ROBINS-I tool, shown in percentages (%) is shown. In the bottom, an overall risk of bias, which represents the collated risk-of-bias judgments for all domains, is depicted

**Fig. 3:**
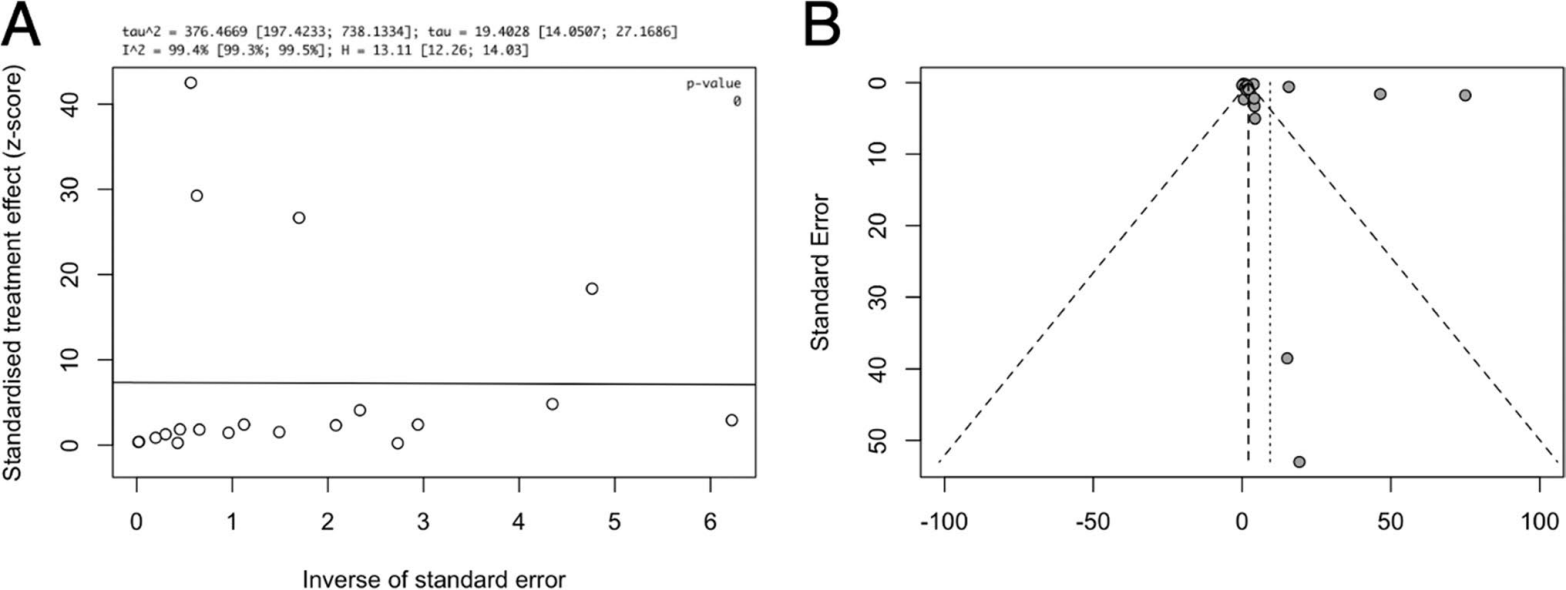
**A** An Egger’s asymmetry plot of all data points included in the meta-analysis (*n* = 20) [2, 5, 14, 16, 20, 23, 26, 27, 33, 34, 59, 60, 62, 64]; 14 original studies but used and counted multiple times due to reporting on multiple radiological markers indicating presence and degree of publication bias is shown. The *x*-axis represents the inverse of standard error, and the *y*-axis the standardized treatment effect (as *z*-score). Furthermore, at the top of the graph different parameters of heterogeneity, including *I*^2^, are shown. *p*-value < 0.05 is deemed to be significant and implicates publication bias. Egger’s asymmetry test yielded *p* = 0, calculated running an Egger’s regression (see Egger’s regression line) on the collated DOR and standard errors of all data used in the meta-analysis (*n* = 20). **B** A funnel plot is shown, which plots every study included in the meta-analysis (*n* = 20). 13 original studies but used and counted multiple times due to some studies reporting on multiple radiological markers) [], particularly their observed effect sizes (diagnostic odds ratio) on the *x*-axis against a measure of their standard error on the y-axis. Visually an n asymmetry is detected, with three outliers lying right and completely outside of the funnel (the right interrupted diagonal line), and two outliers lying right of the mean (vertical interrupted line) but inside the funnel plot

A total of 18,437 papers underwent initial abstract screening for duplicates and exclusion of studies not related to iNPH diagnosis. In the second round of abstract screening, 1554 papers underwent screening and studies not discussing radiological predictors of shunt response in iNPH were excluded. Thirdly, 301 papers received a full-text review, and 28 studies met the inclusion criteria. The pooled sample size of these studies was *n* = 1676 shunted patients (Fig. 1). Nine studies [8, 14, 14, 23, 26, 27, 31, 41, 59] cored a low risk of bias overall using the ROBINS-I [53] while 18 scored moderate [2–5,20,25,34,40,41,46,47,49,50,61Supp-65] risk and 1 study was rated as serious risk [34] (Fig. 2, Supplementary Material: Table 4). The OCEBM analysis scored 17 studies level 2, 6 level 3 and 5 level 2. (Table. 1) Funnel plot asymmetry was detected visually (Fig. 3). Twenty-five different radiological markers were investigated which are presented in Tables 2, 3, 4, 5, 6, 7, 8, 9, 10, 11, 12, 13, 14, 15, 16, 17, 18, 19, 20, and 21 and categorized anatomically and functionally according to the following: variables of the “Rad scale” [28], which is the most updated radiological scale to assess iNPH; DESH, that recently was given high attention in the updated Japanese guidelines [40]; measures of cerebral ventricle size; measures of altered CSF flow; other radiological measures.

**Table 1:** A list of all included studies in this systematic review

Name	Study design	Sample size	NPH criteria	Shunt response criteria	Complications and dropouts	Overall risk of bias (ROBINS-1)	Level of evidence (OCEBM)
Agerskov et al. [2]	Prospective cohort study	*n* = 168	•Patients with probable NPH according to Relkin guidelines	•2 gait (Timed 10 m walk test, Timed Up and Go test) and 2 cognition (Identical Forms test and Bingley Memory test) tests were each scored out of 100 •Composite score calculated using a mean of the 4 test scores •Improvement if increase by ≥ 5 points at 3 or 6 months post shunting	•None reported	Moderate	•1b
Agerskov et al. [3]	Prospective cohort study	*n* = 20	•Patients with probable NPH according to Relkin guidelines were shunted •Assessed using the NPH scale developed by Hellstrom et al	•5-point increase in NPH scale	•1 chronic subdural hematoma	Moderate	•2b
Aoki et al. [4]	Prospective cohort study	*n* = 34	•Patents aged above 60 with one of Hakims triad with enlarged ventricles and DESH on MRI with normal CSF pressure and contents by lumbar puncture	•Gait or cognitive improvement at 6 months using validated scales for both	•None reported	Moderate	2b
Chen et al. [8]	Prospective cohort study	*n* = 28	•Patients with gait disturbance with or without cognitive and urinary disturbance, with ventriculomegaly were shunted •Assessed according to the scale developed by Research Committee on Intractable Hydrocephalus, Ministry of Health and Welfare of Japan, 1996 [36]	•Improvement in the NPH grading scale by 1 or more points before discharge	•3 peri-procedural complications •1 slit-ventricle syndrome •2 seizures post shunting •3-year follow-up: 5 dead, 6 lost to follow up	Low	•1b
Garcia-Armengol et al. [14]	Prospective cohort study	*n* = 89	•Patients between ages 60–85 with one of Hakim’s Triad, ventriculomegaly and B waves in > 10% of time in ICPM were shunted	•1-point improvement in NPH score after 1 year	•1 ICP catheter complication •1 wound infection	Low	•2b
Grahnke et al. [16]	Retrospective cohort study	*n* = 72	•Patients with clinical symptoms and imaging consistent with NPH who responded to CSF TT and underwent VPS were selected	•2-point improvement in Eide scale at 1 year	•27 patients had incomplete follow up were excluded	Low	•2b
Hong et al. [20]	Prospective cohort study	*n* = 31	•Patients with probable NPH according to Relkin guidelines were included •Patients with deep white matter intensities or over aged 85 were excluded	•Improvement ≥ 3 in iNPH total score or ≥ 2 in MRS	•1 death at 1 year and 4 lost to follow up •Acute myocardial infarct and septic shock 11 months from the shunt surgery, acute cholecystitis after 2 weeks, traumatic intracranial hemorrhage after 6 months, shunt revision due to malfunction after 12 months, chronic subdural hemorrhage that needs burr hole drainage after 4 months, and 1 patient expired after 2 months of shunt due to pulmonary embolism •Non-serious adverse events were reported in 7 patients and the most common adverse events were asymptomatic minimal intraventricular and/or intracranial hemorrhage (4 patients) that resolved spontaneously during follow-up	Moderate	•1b
Ishii et al. [23]	Prospective Cohort Study	*n* = 84	•Patients between 60 and 85 with one of Hakims Triad, ventriculomegaly and DESH on imaging with normal content and pressure of CSF on lumbar puncture	• ≥ 1-point improvement on mRS	•None reported	Low	•1b
Jurcoane et al. [25]	Prospective cohort study	*n* = 12	•Patients with probable NPH according to Relkin guidelines underwent 3-day ELD with those improved offered shunting	•Gait and cognitive improvement using DemTect, MMSE and number of steps •Weighted sum of relative improvement to classify patients	•None reported	Moderate	•2b
Kawaguchi et al. [26]	Prospective cohort study	*n* = 100	•Age between 60 and 85 with one or more of Hakims Triad •Presence of Evan’s Index > 0.3 and DESH on MRI. Normal CSF content. All patients underwent CTC	• ≥ 1-point improvement on mRS	•31 dropouts •Thirty patients were excluded from the analysis because of the following: severe adverse events (15 patients), protocol violation within 6 days from the tap test (one patient); lack of record of CTC findings (two patients), and CTC failure (12 patients)	Low	•1b
Kazui et al. [27]	Prospective cohort study	*n* = 100	•Age between 60 and 85 with one or more of Hakims Triad •Presence of Evan’s Index > 0.3 and DESH on MRI. Normal CSF content. All patients underwent CTC and SPECT	• ≥ 1-point improvement on mRS 12 months after surgery	•29 patients excluded from analysis •15 patients suffered complications related to surgery or VP shunt	Low	•2b
Kuchcinski et al. [31]	Prospective cohort study	*n* = 38	•Patients with gait, cognitive and/or urinary impairment with ventriculomegaly were assessed with 2005 Relkin guidelines for probable iNPH and were offered a shunt if they met these guidelines	•2-point improvement on 10-point scale based on Larsson et al. [30] 3 months after surgery	•None reported	Low	•3b
Mantovani et al. [33]	Prospective cohort study	*n* = 62	•Probable NPH according to Relkin guidelines underwent TT and had a positive response were shunted. Patients underwent gait assessment including: 18 m walking test, (TUG-T), and Tinetti POMA scale as well as mRs and INPHGS grading	• ≥ 5-point increase in Tinetti POMA total score	•15 lost to follow-up	Moderate	•2b
McGirt et al. [34]	Prospective cohort study	*n* = 132	•Patients with 2 or more of Hakims triad with ventriculomegaly underwent pCSF monitoring and ELD. Patients were offered shunting if no pathological waves on pCSF monitoring and an objective improvement after ELD	•Improvement in one of triad symptoms at 6 months. Cognition: 3-point increase in MMSE. Improvement in urinary symptoms (decrease in incidence of urinary frequency, urgency, or incontinence). Gait examined using objective tests	•20 (15%) had headaches. Three (2%) had subdural hematoma. One (1%) frontal lobe hematoma leading to pulmonary embolism	Serious	•2b
Murakami et al. [39]	Prospective cohort study	*n* = 24	•Any combination of Hakim triad with confirmed ventriculomegaly (Evan’s Index > 0.3) on CT and replicable clinical improvement on two separate diagnostic TT	• ≥ 1 rank improvement in at least two separate categories of Mori scale	•None reported	Moderate	•3b
Narita et al. [41]	Retrospective cohort study	*n* = 103	•Symptomatic hydrocephalus with ≥ 1 of the triad of symptoms AND neuro imaging features of disproportionately enlarged subarachnoid space hydrocephalus (DESH) on MRI •Following neurocognitive and initial imaging patients underwent further 3D volumetric MRI, SPECT and CSF TT	• ≥ 1-point improvement on the iNPHGS, ≥ 10% increase in TUG time, and ≥ 3 points MMSE improvement after 1 year	•43 lost: 2 deaths, 7 complications: shunt system problems, 1 femoral fracture, 2 pneumonia, 1 cerebral infarction. 29 lost to follow-up. 4 had incomplete data	Low	•3b
Palm et al. [42]	Prospective cohort study	*n* = 26	•Patients with wide stepping gate or shuffling gate and dilated ventricles and frontal horn index > 0.4	•Clinical rating at 12 months	•3 died in follow-up period and LTFU	Moderate	•2b
Black. [5]	Retrospective cohort study	*n* = 62	•Patients with gait disturbance with enlarged ventricles had LP and if the pressure was < 180 mmH20 were offered a shunt	•Either improvement in Stein and Langfitt’s grading or a separate scale which compares to pre-illness morbidity. Mean follow-up of 36.5 months	•21 patients with complication. 1 sub-Dural hematoma. 7 sub-Dural collections. 4 had seizures post-op. 3 had transient neurological disturbances. 1 pneumonia and 1 transient pulmonary oedema. 5 deaths: MI, PE, aspiration pneumonia, cerebral infarct and unknown cause	Moderate	•2b
Poca et al. [45]	Prospective cohort study	*n* = 35	•All patients presented with all three of Hakim's Triad and ventriculomegaly went on to have ICPM. Those with active or compensated hydrocephalus (Mean ICP > 12 mmHg or pathological waves present) were shunted	•Improvement in “functional scales and neurophysiological tests” 6 months after surgery	•None reported	Moderate	•2b
Poca et al. [46]	Prospective cohort study	*n* = 43	•Patients with one of hakims triad of symptoms or parkinsonism refractory to medical treatment and ventriculomegaly on imaging were subject to ICPM. Patients with active or compensated hydrocephalus (I.e., presence of pathological waves or mean ICP > 12 mmHg)	•Improvement of 1 or more points in NPH scale	•6 (14%) complications. Early: 2 headaches, 1 sub-Dural hematoma. Late: three bilateral subdural collections. None LTFU	Moderate	•3b
Shinoda et al. [51]	Retrospective cohort study	*n* = 55	•Patients between 60 and 85 with one of Hakims Triad, ventriculomegaly and DESH on imaging with normal content and pressure of CSF on lumbar puncture. Secondary outcomes: ≥ 1 points on the iNPHGS, ≥ 3 points on MMSE, a decrease of > 30% on TMT-A, and a decrease > 10% on TUG-t post shunt	•improvement of: ≥ 1 on mRS,	•3 complications: Traumatic intracranial hemorrhage, acute ischemic stroke, aggravation of cirrhosis. 2 lost to follow-up	Moderate	•2b
Stecco et al. [52]	Retrospective cohort study	*n* = 38	•Patients with two or more features of Hakim's Triad and an Evan’s Index > 0.3 on MRI were offered a shunt	•Decrease of at least 2 points in the union of gait and urinary incontinence scales or a decrease of 1 point in either urinary incontinence or gait scales and > 2 increase in MMSE score	•None reported	Moderate	•2b
Virhammar et al., 2014 [60]	Retrospective cohort study	*n* = 108	•Hakim Triad and ventriculomegaly on imaging in absence of other neurological co-morbidities	•Any of: Motor function improvement of ≥ 1 on gait/ balance scale or ≥ 20% reduction in time/ number of steps in ≥ 50% in 3 tests; Cognition ≥ 4 improvement in MMSE; Continence scale ≥ 1 level and improvement in MMSE score ≥ 2	•29 had shunt related complications, 5 had co-morbidity related complications	Low	•2b
Virhammar et al., 2014 [59]	Retrospective cohort study	*n* = 108	•Hakim Triad and ventriculomegaly on imaging in absence of other neurological co-morbidities. CSF TT and LIT were used to assist selection	•Any of: Motor function improvement of ≥ 1 on gait/ balance scale or ≥ 20% reduction in time/ number of steps in ≥ 50% in 3 tests; Cognition ≥ 4 improvement in MMSE or > 2–3 for possible improvement; Continence scale ≥ 1 level improvement	•36 lacked preoperative MRI and 28 were not assessed after 12 months •29/109 (27%) had complications: 1 (1%) intracerebral hematoma, 10 (9%) subdural hematomas, 2 (2%) shunt infection treated by shunt revision, 16 (15%) underwent surgery due to proximal or distal catheter failure. Co-morbid complications: 1 stroke with motor symptoms, 1 lung resection, 1 radical cystectomy, 1 lower limb amputation, 1 femur fracture	Moderate	•2b
Wu et al. [61]	Retrospective cohort study	*n* = 41	•Patients with probable NPH according to classical symptoms with ventriculomegaly were offered shunting if they had a positive response to TT •Feature selection was performed on the training cohort (those who improved in TUG or Tinetti gait scale post 2 h CSF TT) •Recursive feature elimination (RFE), a type of machine learning was used to identify features which can predict drainage response •Performance of the algorithm was tested on the prognostic cohort who were shunted. Least absolute shrinkage and selection operator (LASSO) method was used to select optimal features which would predict Tinneti and MMSE score •NB the model also used age, gender, test score before shunting, the time between shunt surgery and post-surgical test, and time between the pre-surgical MRI and shunt surgery as input variables	•Improvement in Tinetti scale or MMSE score	•None reported	Moderate	•2b
Yamada et al. [62]	Prospective cohort study	*n* = 25	•One of hakims triad, ventriculomegaly and either positive tap test or cisternography	•Improvement in MMSE of 3 points or more	•None reported	Moderate	•2b
Yamamoto et al. [63]	Retrospective cohort study	*n* = 16	•Patients > 60 years with one or more of Hakims triad and ventriculomegaly and tightness of the high convexity who responded to shunting were included	•Improvement in: iNPHGS, TUG-T,10 m reciprocating walking test, MMSE, Alzheimer’s disease assessment scale, frontal assessment battery and trail making test A	•None reported	Moderate	•3b
Ziegelitz et al. [64]	Prospective cohort study	*n* = 22	•Patients with gait disturbance with cognitive or urinary dysfunction and ventriculomegaly were offered a shunt. Patients were assessed using the NPH scale	•Improvement in NPH scale of 5 points or more	•2 dropouts due to artifacts in imaging data	Moderate	•3b

An overview is provided on NPH diagnosis criteria, shunt response specification and complication rates of reported by all included studies. *SR*, shunt response; *S-NR*, shunt non-response; *CSF*, cerebrospinal fluid; *VPS*, ventriculoperitoneal shunt; *DESH*, disproportionately enlarged subarachnoid space hydrocephalus; *iNPHGS*, idiopathic normal-pressure hydrocephalus grading scale; *ELD*, external lumbar drainage; *MMSE*, mini mental state examination; *Tinetti POMA*, Tinetti performance oriented mobility assessment (POMA); *mRS*, modified Rankin scale; *CTC*, computerized tomographic cisternography; *TT*, tap test; *LP*, lumbar puncture; *SPECT*, single-photon emission computerized tomography; *ICPM*, intracranial pressure monitoring; *LIT*, lumbar infusion test; *TUG-t*, timed up and Go test; *TMT-A*, trail making test A

**Table 2:** The use of Evan’s Index on MRI and CT for prediction of shunt response in iNPH

Study	Sample size	Radiological methodology	Cutoff specification	Image specification	Image plane	Main reported outcomes
Narita et al. [41]	*n* = 103	•Ratio between max diameter of the frontal horns of lateral ventricles to the max skull inner diameter	N/A	•3D T1-weighted MRI obtained with a Signa 1.5 T MR imaging unit	•Transverse plane	•No significant association between Evan’s Index and post-surgical improvement reported. (Regression coefficient for total score, gait, cognitive, urinary subsections, TUG and MMSE was 7.34, 1.00, 0.97, 5.37, 60.96, 9.22 respectively *p* > 0.1)
Virhammar et al., 2014[60]	*n* = 108	•Ratio between max diameter of the frontal horns of lateral ventricles to the max skull inner diameter	N/A	•T2 Flair, T1-weighted MRI •9% of patients on 3 T scanner; 70% on a 1.5 T scanner, 14% on a 1 T scanner and 7% on a 0.5 T scanner	•Transverse plane	•*OR* between SR and SNR: 1.57 (0.97–2.52), *p* = 0.064)
Hong et al. [20]	*n* = 31	•Ratio between the max diameter of frontal horns of the lateral ventricles and the max inner diameter of the skull	N/A	•3.0 Tesla MRI scanner was used to gain axial FLAIR, T2- weighted images, T1-weighted images, and coronal T1-weighted images	•Transverse plane	•There was no significant difference in Evan’s Index between SR and SNR. SR had mean Evan’s Index of 0.37 ± 0.04 while SNR had mean Evan’s Index 0.37 ± 0.03 (*p* = 0.77)
Agerskov et al. [2]	*n* = 168	•Ratio between max diameter of frontal horns and the max inner skull diameter	N/A	•MRI 1.5 T •Trans-axial T1-weighted images	•Transverse plane in slice above the foramen of Monro	•All patients had Evan’s Index > 0.3 •There was no difference in Evan’s Index findings between SR (median 0.4) and SNR (median 0.39) [*p* > 0.05] and it could not be used to predict SR in multivariate logistical analysis •Its non-significant correlation coefficient with the composite score was -0.09
Wu et al. [61]	*n* = 41	•Not given	N/A	•High-resolution T1-weighted MRI	•Not given	•The model predictions using Evan’s Index alone with co-variates mentioned in methodology showed a low correlation with the ground truth (*r* = 0.48 for the Tinetti and *r* = 0.80 for MMSE) •When the co-variates were removed from the input, the prediction accuracy was 0.42 and 0.46 for the Tinetti and MMSE respectively

Studies included assessing the use of any MRI or CT Evan’s Index as predictor of shunt responsiveness. *SR*, shunt response; *S-NR*, shunt non-response; *FLAIR*, Fluid-attenuated inversion recovery; *MMSE*, mini mental state examination; *TUG*, timed up and go test

**Table 3:** The use of the callosal angle on MRI and CT for prediction of shunt response in iNPH

Study	Sample size	Radiological methodology	Cutoff specification	Image specification	Image plane	Main reported outcomes
Mantovani et al. [33]	*n* = 62	•Callosal angle: level of the posterior commissure orthogonal to the anterior commissure-posterior commissure (ACPC) line •Anterior callosal angle: at the level of the anterior commissure orthogonal to ACPC line	•CA: 59.5° •ACA: 112°	•3 T MRI	•Coronal plane	•Mean ACA was higher than mean CA, 103.6° ± 14.2° vs 58.9° ± 16.1° (*p* < 0.001) •Neither CA or ACA were found to significantly predict mRs or INPHGS outcome ACA: •There was a negative correlation between ACA and Tinetti total score (r = -0.306, *p* < 0.05) •Mean ACA in SR patients was smaller than SNR patients, (98.3° ± 11.4° and 108.6° ± 15.1° respectively) •Mean ACA was significantly smaller in those with reduced fall risk post shunt •Using a cutoff has a Youden’s Index = 0.344. The *OR* for ACA between SR and SNR is 2.97 (95% *CI* 1.04–8.5), CA: •There were no significant differences between SR. Values not given •There was no effect of CA size on fall risk post shunt •Using a cutoff shows Youden’s Index = 0.327. The *OR* between SR and SNR is 2.15 (95% *CI* 1.03–4.52)
Virhammar et al., 2014 [60]	*n* = 108	•Angle between lateral ventricles through the posterior commissure	•N/A	•MRI. T2 Flair, T1-weighted MRI. (9% of patients on 3 T scanner; 70% on a 1.5 T scanner, 14% on a 1 T scanner and 7% on a 0.5 T scanner	•Coronal plane, image taken perpendicular to the anterior/ posterior commissure plane	•*OR* between SR and SNR: 0.57[(0.36–0.91) *p* = 0.017]
Virhammar et al., 2014 [59] Callosal angle only	*n* = 108	•Angle between the lateral ventricles through the posterior commissure	•63°	•MRI 3D T1-weighted images. Ten (9%) on 3-T, 75 (69%) on 1.5-T, 16 (15%) on 1-T, and 8 (7%) on 0.5-T	•Coronal plane through posterior commissure, perpendicular to the anterior commissure -Posterior commissure plane	•CA was significantly smaller in SR [59° (95% *CI* 56°–63°)] than SNR [68° ( •95% *CI* 61°–75°)] (*p* < 0.05) •Multivariate analysis: Smaller CA was significantly associated SR (*OR* 0.97, [95% *CI* 0.94–0.99], *p* < 0.05) Cutoff: Sensitivity: 0.67, Specificity:0.65, Youden’s index: 0.33 •TP: 55, TN: 18, FP: 9, FN:27. N = 109 •Weak inverse correlation between EI and CA (r = -0.23, *p* < 0.05)
Narita et al. [41]	*n* = 103	•Angle between the left and right corpus callosum	•N/A	•3D T1-weighted MRI obtained with a Signa 1.5 T MR imaging unit	•Coronal plane at the posterior commissure	•Simple linear regression analysis showed significant association between CA and MMSE improvement (B = − 0.04, *R*^2^ = 0.08, *p* = .035). There was no significant association between CA and Total score, gait cognitive or urinary subjections or TUG (B = − 0.02, − 0.01, − 0.01, − 0.01, − 0.12 respectively)
Hong et al. [20]	*n* = 31	•Angle between the lateral ventricles	•N/A	•3.0 Tesla MRI scanner was used to gain Axial fluid-attenuated inversion recovery (FLAIR), T2- weighted images, T1-weighted images, and coronal T1-weighted images	•Coronal plane through posterior commissure, perpendicular to the anterior commissure -Posterior commissure plane	•Difference in mean CA in SR: 75.2 ± 21.7 and in SNR groups: 88.3 ± 18.2 was not significant (*p* = 0.109). No other statistical analysis was performed
Agerskov et al. [2]	*n* = 168	•N/A	•N/A	•MRI 1.5 T. T1-weighted images	•Coronal plane at level of the posterior commissure	•All patients had CA < 90° •There was no difference in CA findings between SR (median 68°) and SNR (median 69°) [*p* > 0.05] and it could not be used to predict SR in multivariate logistical analysis •Its non-significant correlation coefficient with the composite score was 0.17
Grahnke et al. [16]	*n* = 72	•Angle at the level of midpoint of corpus collosum	•105.4°	•CT or MRI	•Mid-sagittal plane, parallel to floor of 4th ventricle	•Mean CA in SR was 108.4 (SD: 16.8) while SNR was 117.6 (SD: 14.2), *p* = 0.030. Diagnostic accuracy AUC of 0.64 95% *CI* (0.50–0.78). Cutoff of 105.4 has sensitivity 0.415 and a specificity of 0.87. A patient is 4% more likely to have post-shunt benefit for every degree CA is lower: *OR* (unadjusted) 0.96 [(95% *CI*:0.93–0.998) *p* = 0.037], adjusted *OR*: 0.96 [(95% *CI*: 0.93–0.997) *p* = 0.036] •TP: 19, TN:23, FP:4, FN:26
Black [5]	*n* = 62	•Angle of the junction of frontal horn roofs	•120°	•Pneumoencephalogram	•AP projection	•There was no significant difference in CA between SR and SNR. The cutoff had a sensitivity of 50%, specificity of 60%, PPV of 42.9% and NPV of 66.7%. TP:3, FP:4, TN:6, FN:3

Studies included assessing the use of any MRI or CT callosal angle as predictor of shunt responsiveness. MRI studies are above the double solid lines, CT studies are below. *SR*, shunt response; *S-NR*, shunt non-response; *ACA*, anterior callosal angle; *CA*, callosal angle; *mRS*, modified Rankin scale; MMSE, mini mental state examination; *TUG*, timed up and go test; *OR*, odds ratio; *CI*, Confidence interval; *AUC*, area under the curve; *SD*, standard deviation; *NPV*, negative predictive value; *PPV*, positive predictive value; *TP*, true positives; *FP*, false positives; *TG*, true negatives; *FN*, false negatives

**Table 4:** The use of periventricular white matter changes prediction of shunt response in iNPH

Study	Sample size	Radiological methodology	Cutoff specification	Image specification	Image plane	Main reported outcomes
Poca et al	*n* = 43	•Periventricular lucencies notes in frontal or other locations	•N/A	•CT	•N/A	•22 (51%) patients had periventricular lucencies. 10 (23%) in the frontal horns and 12 (28%) in frontal and other areas •Patients with lucencies in frontal and other areas showed improvement in NPH scale (one-way ANOVA: 7.56, *p* = 0.002), the Memory Quotient (one-way ANOVA: 6.21, *p* = 0.006), and the Orientation part of the WMS (chi-square = 11.41, *p* = 0.003), compared with no lucencies or just frontal lucencies
McGirt et al. [34]	*n* = 132	•Not given	•N/A	•CT/MRI	•N/A	•58 (44%) patients had periventricular white matter changes. There was no significant relation between SR and periventricular white matter change. Univariate RR: 1.11 (95% *CI*: 0.74–1.66)
Agerskov et al. [2]	*n* = 168	•Evaluated using ordinal scale graded 0–3	•N/A	•MRI 1.5 T. Trans-axial FLAIR images	•Trans-axial	•0% of patients had grade 0, 57% had grade 1, 27% had grades 2 and 17% had grade 3 •There was no difference, in each grade, between SR and SNR
Narita et al. [41]	*n* = 103	•According to Fazekas et al. [12]	•N/A	•3D T1-weighted MRI obtained with a Signa 1.5 T MR imaging unit	•Transverse plane	•No significant association with post-surgical improvement reported. (Regression coefficient for total score, gait, cognitive, urinary subsections, TUG and MMSE was − 0.33, − 0.05, − 0.10, − 0.19, 3.19, − 0.25 respectively *p* > 0.1)
Virhammar et al. [60]	*n* = 108	•Periventricular hyperintensities along and in contact with the frontal and parietal portions of the lateral ventricles •Graded 0–2 •0 = normal, including “pencil-thin lining” along the ventricular wall and small caps around the frontal horns •1 = Increased PVH •2 = Irregular large symmetric hyperintensities	•N/A	•T2- FLAIR MRI. (9% of patients on 3 T scanner; 70% on a 1.5 T scanner, 14% on a 1 T scanner and 7% on a 0.5 T scanner	•Transverse plane in center of 3rd ventricle in AP direction	•*OR* between SR and SNR: 0.82 (0.39–1.72), *p* = 0.6) was statistically insignificant
Hong et al. [20]	*n* = 31	•Measured using Fazekas et al. [12] ordinal scale from 0–3	•N/A	•3.0 Tesla MRI scanner was used to gain Axial fluid-attenuated inversion recovery (FLAIR), T2- weighted images	•Transverse	•There was no significant difference between SR and SNR within each grade (*p* = 0.947). Grade 1 had 10 SR and 7 SNR, Grade 2 had 5 SR and 4 SNR, Grade 3 had 2 SR and 2 SNR •*OR* 0.600 (0.039–9.156) *p* = 0.713

Studies included assessing the use of any advanced imaging radiological marker as predictor of shunt responsiveness. *SR*, shunt response; *S-NR*, shunt non-response; *WMS*, Wechsler Memory Scale; *TUG*, timed up and go test; *FLAIR*, fluid-attenuated inversion recovery; *ANOVA*, analysis of variance; *RR*, risk ratio

**Table 5:** The use of absence of dilated cortical sulci for prediction of shunt response in iNPH

Study	Sample size	Radiological methodology	Cutoff specification	Image specification	Image plane	Main reported outcomes
Black [5]	*n* = 62	•N/A	•Absence of sulcal enlargement	•Used both pneumoencephalogram (PEG) and CT	•N/A	•PEG: There was no significant difference between SR and SNR. Sensitivity 66.7%, specificity: 35.7%, PPV:40% and NPV: 62.5%. TP 6, TN5, FP9, FN 3 •CT**:** Significant difference between SR and SNR, with those with no sulcal enlargement more likely to respond to shunt. Sensitivity 78.6%, specificity: 75.0%, PPV 84.6% and NPV 66.7%. TP: 11, TN:6, FP2, FN3
Poca et at. [46]	*n* = 43	•Scan categorized into normal, obliterated or enlarged cortical sulci	•N/A	•CT	•N/A	•Sulci were normal in 11, obliterated in 5 and enlarged in 27. There was a significant difference between groups in the information subset of WMS (chi-square = 10.05, *p* = 0.007). Those with enlarged sulci were less likely to improve in neurocognitive tests after shunting
Agerskov et al. [2]	*n* = 168	•Obliteration of high convexity sulci: No sulci on 10 most cranial slices covering vertex •Transport sulci: Focally enlarged sulci in absence of atrophy. Numbered as 0,1,2, > 2	•N/A	•MRI 1.5 T. T1-weighted images	•Trans-axial and coronal	Obliterated sulci: •There was no significant difference SR (36% had obliterated sulci) vs. SNR (35%) •There was also no significant correlation with the composite score. (0.19, 0.18, 0.13 for total, gait, and cognition respectively.) Transport sulci**:** •72% had 0, 17% 1 and 8% had 2 and 3% had > 2. There was no difference between SR and SNR in any of these categories •There was also no significant correlation with the composite score. (0.07, 0.18, 0.08 for total, gait, and cognition respectively.)
Narita et al. [41]	n = 103	•Presence (1) or absence (0) noted of focal cortical sulci were evaluated	•N/A	•3D T1-weighted MRI obtained with a Signa 1.5 T MR imaging unit	•Transverse plane	•No significant association with post-surgical improvement reported. (Regression coefficient for total score, gait, cognitive, urinary subsections, TUG and MMSE was 0.71, 0.19, 0.00, 0.53, − 2.25, 1.19 respectively *p* > 0.1)
Virhammar et al. [60]	*n* = 108	•Accumulation of CSF in focally enlarged sulci was graded as present or absent	•N/A	•9% of patients on 3 T scanner; 70% on a 1.5 T scanner, 14% on a 1 T scanner and 7% on a 0.5 T scanner	•N/A	•*OR* between SR and SNR [0.54 (0.63–3.73), *p* = 0.34] was statistically insignificant

Studies included assessing the use of any advanced imaging radiological marker as predictor of shunt responsiveness. *SR*, shunt response; *S-NR*, shunt non-response; *PEG*, pneumoencephalogram; *CSF*, cerebrospinal fluid; *MMSE*, mini mental state examination; *TUG*, timed up and go test; *WMS*, Wechsler Memory Scale; *NPV*, negative predictive value; *PPV*, positive predictive value; *TP*, true positives; *FP*, false positives; *TG*, true negatives; *FN*, false negatives

**Table 6:** The use of Sylvian fissure size for prediction of shunt response in iNPH

Study	Sample size	Radiological methodology	Cutoff specification	Image specification	Image plane	Main reported outcomes
Poca et al., 2004 [45]	*n* = 43	•Sylvian fissures were categorized into normal, obliterated or dilated	•N/A	•CT	•N/A	•13 patients had normal fissures, 29 had dilated fissures while 1 was obliterated. Patients with normal fissures showed greater improvement in Trail Making Test B (chi-square test: 7.18, *p* = 0.007)
Agerskov et al., 2019 [2]	*n* = 168	•Graded from 0–2	•N/A	•MRI 1.5 T. trans-axial T1-weighted images	•Axial slice	•28% of patients had grade 0, 45% grade 1 and 27% grade 2 •There was no difference, in each grade, between SR and SNR
Narita et al., 2016 [41]	*n* = 103	•Graded on visual ordinal scale from 0–3: 0 – narrowed, 1- normal; 2—mildly dilated, and 3—severely dilated	•N/A	•3D T1-weighted MRI obtained with a Signa 1.5 T MR imaging unit	•Transverse and axial	•Simple linear regression analysis: There was an association between Sylvian fissure dilation and change in iNPHGS gait (B = 0.59, R2 = 0.08, *p* = .029). There was no association with total score, cognitive or urinary subjection, or TUG or MMSE. (1.03, − 0.09, 0.53, − 2.65, 1.00 respectively)
Virhammar et al., 2014 [60]	*n* = 108	•Ordinal scale: Graded 0–3 evaluated at level of central part of brain stem, angulated along brain stem •Height: Mean (mm) measurement at 5 different locations perpendicular to fissure direction in midpoint between skull and insular cortex	•N/A	•T2 Flair, T1-weighted MRI. (9% of patients on 3 T scanner; 70% on a 1.5 T scanner, 14% on a 1 T scanner and 7% on a 0.5 T scanner	•Ordinal: Coronal •Height: Sagittal	•Ordinal: *OR* between SR and SNR [ 1.35 (0.57–3.21), *p* = 0.5] was not significant •Height: *OR* between SR and SNR [1.20 (0.59–2.43), *p* = 0.62] was not significant

Studies included assessing the use of any advanced imaging radiological marker as predictor of shunt responsiveness. *SR*, shunt response; *S-NR*, shunt non-response; *MMSE*, mini mental state examination; *TUG*, timed up and go test; *iNPHGS*, idiopathic normal-pressure hydrocephalus grading scale

**Table 7:** The use of Temporal Horn Size for prediction of shunt response in iNPH

Study	Sample size	Radiological methodology	Cutoff specification	Image specification	Image plane	Main reported outcomes
Poca et al., 2004 [45]	*n* = 43	•Temporal horns were categorized into normal or enlarged	•N/A	•CT	•N/A	•10 patients had normal horns while 33 had enlarged horns. There was no difference in outcome between the two groups
Agerskov et al., 2019 [2]	*n* = 168	•Maximum diameter	•N/A	•MRI 1.5 T. trans-axial T1-weighted images	•Axial slice	•There was no difference between SR (median 9.0 mm) and SNR (median 9.1 mm) [*p* > 0.05] and it could not be used to predict SR in multivariate logistical analysis
Virhammar et al., 2014 [60]	*n* = 108	•Average of left and right max diameter of temporal horns in mm	•N/A	•T2 FLAIR, T1-weighted MRI. (9% of patients on 3 T scanner; 70% on a 1.5 T scanner, 14% on a 1 T scanner and 7% on a 0.5 T scanner	•Transverse plane	•*OR* between SR and SNR: 1.84 (1.11–3.03), *p* = 0.018) was statistically significant

Studies included assessing the use of any advanced imaging radiological marker as predictor of shunt responsiveness. *SR*, shunt response; *S-NR*, shunt non-response; *FLAIR*, fluid-attenuated inversion recovery

**Table 8:** The use of high-convexity tightness alone for prediction of shunt response in iNPH

Study	Sample size	Radiological methodology	Cutoff specification	Image specification	Image plane	Main reported outcomes
Narita et al. [41]	*n* = 103	•Observed in 4 uppermost contiguous transverse sections and 3 contiguous coronal sections anterior to and on the posterior commissure. Graded on ordinal scale from 0–3	•N/A	•3D T1-weighted MRI obtained with a Signa 1.5 T MR imaging unit	•Transverse and Coronal	•Simple linear regression analysis: presurgical high-convexity tightness was associated with change in iNPHGS total score (regression coefficient [B] = 1.23, coefficient of determination [R2] = 0.13, *p* = .004), change in iNPHGS gait score (B = 0.59, R2 = 0.16, *p* = .002), and change in MMSE (B = 2.56, R2 = 0.17, *p* = 0.001) •Multiple linear regression analysis: high convexity tightness predicted change in the iNPHGS total score (B = 0.99, R2 = 0.24, *p* = .017) and the gait score (B = 0.52, R2 = 0.21, *p* = .006)
Virhammar et al. [60]	*n* = 108	•Graded 0–2 •0 = Normal or wider than normal •1 = slight compression •2 = definitive compression	•N/A	•T2 Flair, T1-weighted MRI. (9% of patients on 3 T scanner; 70% on a 1.5 T scanner, 14% on a 1 T scanner and 7% on a 0.5 T scanner	•Coronal and transverse plane	•*OR* between SR and SNR [1.43 (0.84–2.46), *p* = 0.2]

Studies included assessing the use of any advanced imaging radiological marker as predictor of shunt responsiveness. *SR*, shunt response; *S-NR*, shunt non-response; *FLAIR*, fluid-attenuated inversion recovery; *iNPHGS*, idiopathic normal-pressure hydrocephalus grading scale; *MMSE*, mini mental state examination

**Table 9:** The use of ventricular size excluding Evan’s Index for prediction of shunt response in iNPH

Study	Sample size	Radiological methodology	Cutoff specification	Image specification	Image plane	Main reported outcomes
Black [5]	*n* = 62	•Ventricular span	• > 55 mm	•Pneumoencephalogram (PEG)	•N/A	•There was no significant difference in those with spans over or under 55 mm between SR and SNR. Sensitivity: 90%, specificity 44.4%, PPV 50%, NPV 88.9%. TP 10, TN 8, FP 10 FN 1
Poca et al. [46]	*n* = 43	•Ventricular score: composite of maximal bifrontal distance, distance between the caudate nuclei at the level of the foramen of Monro, maximal width of the third ventricle, minimal width of both cella media, maximal inner diameter of the skull at the level of the measurement of the maximal bifrontal distance	•N/A	•CT	•N/A	•There was a positive correlation between score and % change on the Digit Span Forward attention test (rho = 0.46, *p* = 0.002)
Agerskov et al. [2]	*n* = 168	•Widest diameter of 3rd ventricle between anterior and posterior commissures •Widest AP diameter of 4th ventricle	•N/A	•MRI 1.5 T.T1-weighted images	•3rd ventricle: coronal slice •4th ventricle le: Sagittal	•There was no significant difference in 3^rd^ or 4^th^ ventricle size between SR and SNR •3^rd^ ventricle size: SR: 15.4 mm SNR: 16.5 mm •4^th^ ventricle: SR: 14.7 mm, SNR: 14.6 mm
Virhammar et al. [60]	*n* = 108	•Maximum 3rd ventricle diameter in widest part of inferior-superior direction	•N/A	•T1-weighted MRI. (9% of patients on 3 T scanner; 70% on a 1.5 T scanner, 14% on a 1 T scanner and 7% on a 0.5 T scanner	•Transverse plane in center of 3rd ventricle in AP direction	•*OR* between SR and SNR: 1.24 (0.78–1.97), *p* = 0.37) was statistically insignificant

Studies included assessing the use of any advanced imaging radiological marker as predictor of shunt responsiveness. *SR*, shunt response; *S-NR*, shunt non-response; *PEG*, pneumoencephalogram; *NPV*, negative predictive value; *PPV*, positive predictive value; *TP*, true positives; *FP*, false positives; *TG*, true negatives; *FN*, false negatives

**Table 10:** The use of flow void for prediction of shunt response in iNPH

Study	Sample size	Radiological methodology	Cutoff specification	Image specification	Image plane	Main reported outcomes
Agerskov et al. [2]	*n* = 168	•Void in Cerebral aqueduct and fourth ventricle. Evaluated using ordinal scale graded 0–3	•N/A	•MRI 1.5 T. T1-weighted images	•Coronal slice	•0% of patients had grade 0, 30% had grade 1, 42% had grade 2 and 28% had grade 3 •There was no significant difference, in each grade, between SR and SNR
Virhammar et al. [60]	*n* = 36	•Ordinal scale: Graded 0–3. 0 = no flow, 1 = flow void only in the aqueduct, 2 = flow void in the aqueduct and upper half of the fourth ventricle, 3 = flow that extends to caudal part 4th ventricle	•N/A	•T2-weighted images	•Sagittal images without flow compensation	•*OR* between SR and SNR: 4.25 (0.75–23.97), *p* = 0.1) was not significant

Studies included assessing the use of any advanced imaging radiological marker as predictor of shunt responsiveness. *SR*, shunt response; *S-NR*, shunt non-response

**Table 11:** The use of bulging of the lateral ventricular roof for prediction of shunt response in iNPH

Study	Sample size	Radiological methodology	Cutoff specification	Image specification	Image plane	Main reported outcomes
Narita et al., 2016[41]	*n* = 103	•Presence (1) or absence (0) noted at level above top of thalamus	•N/A	•3D T1-weighted MRI obtained with a Signa 1.5 T MR imaging unit	•Transverse plane	•No significant association with post-surgical improvement reported. (Regression coefficient for total score, gait, cognitive, urinary subsections, TUG and MMSE was 0.47, 0.31, 0.11, 0.05, 2.20, 1.20 respectively *p* > 0.1)
Virhammar et al., 2014 [60]	*n* = 108	•On roof of lateral ventricles. Graded as present or absent	•N/A	•T2 Flair, T1-weighted MRI. (9% of patients on 3 T scanner; 70% on a 1.5 T scanner, 14% on a 1 T scanner and 7% on a 0.5 T scanner	•Sagittal	•*OR* between SR and SNR: 3.22 (0.97–10.69), *p* = 0.055) was not significant

Studies included assessing the use of any advanced imaging radiological marker as predictor of shunt responsiveness. *SR*, shunt response; *S-NR*, shunt non-response; *FLAIR*, fluid-attenuated inversion recovery; *MMSE*, mini mental state examination; *TUG*, timed up and go test

**Table 12:** The use of CSF flow dynamics on MRI and CT for prediction of shunt response in iNPH

Study	Sample size	Radiological methodology	Cutoff specification	Image specification	Image plane	Main reported outcomes
Stecco et al., 2020 [52]	*n* = 38	•Aqueductal stroke volume (ACSV)	•N/A	•FFE 3D T2 weighted sequence on phase contrast cine MRI	•Sagittal plane	•Mean ACSV value in SR was 271.85 (± 143.032) while in SNR was 79.83 (± 31.24), *p* < 0.01
Poca et al., 2002 [45]	*n* = 35	•CSF flow velocity	•Hyperdynamic: aqueductal CSF peak velocities are > 97.5 percentile of control group of heathy volunteers	•Phase-Contrast Velocity MRI with Retrospective Cardiac Gating	•Sagittal plane through the aqueduct	•29 (83%) NPH patients were considered hyperdynamic. Hyperdynamic CSF velocity had a sensitivity 90%, specificity 50%, PPV 95.6& and NPV 25%. TP28, TN1, FN3, FP1

Studies included assessing the use of any MRI or CT CSF flow dynamics analyses as predictor of shunt responsiveness. MRI studies are above the double solid lines, CT studies are below. *SR*, shunt response; *S-NR*, shunt non-response; *ACSV*, aqueductal stroke volume; *NPV*, negative predictive value; *PPV*, positive predictive value; *TP*, true positives; *FP*, false positives; *TG*, true negatives; *FN*, false negatives

**Table 13:** The use of cerebral atrophy for prediction of shunt response in iNPH

Study	Sample size	Radiological methodology	Cutoff specification	Image specification	Image plane	Main reported outcomes
McGirt et al. [34]	*n* = 132	•Not given	•N/A	•CT/MRI	•N/A	•30 (28%) of patients had diffuse cerebral atrophy, however it was not associated with a change in its outcome, univariate analysis showed a RR: 1.13 (95% *CI*: 0.69–1.83)
Hong et al. [20]	*n* = 3	•Hippocampal atrophy was measures suing Scheltens visual grading scale from 0–4. 4 being most atrophied	•N/A	•3.0 Tesla MRI scanner. T- Weighted image	•Coronal	•There was no significant difference between SR and SNR (*p* = 0.831). SR mean grade was 1.8 ± 0.9 while SNR was 1.9 ± 0.6. Univariate analysis showed an *OR*: 0.534 •(95% *CI*: 0.169–1.693) was insignificant at *p* = 0287

Studies included assessing the use of any advanced imaging radiological marker as predictor of shunt responsiveness. *SR*, shunt response; *S-NR*, shunt non-response; *RR*, risk ratio; *OR*, odds ratio; *CI*, confidence interval

**Table 14:** The use of deep white matter hyperintensities for prediction of shunt response in iNPH

Study	Sample size	Radiological methodology	Cutoff specification	Image specification	Image plane	Main reported outcomes
Agerskov et al. [2]	*n* = 168	•Evaluated using ordinal scale graded 0–3	•N/A	•MRI 1.5 T. Trans-axial FLAIR images	•Trans-axial	•0 had grade 0, 46% had grade 1, 37% had grade 2 and 17% had grade 3. There was no difference, in each grade, between SR and SNR •There were significant (*p* < 0.05) but weak negative correlations between grade and composite score. (-0.22, -0.21, -0.17 for total, gait, and cognition respectively.)
Narita et al. [41]	*n* = 103	•According to Fazekas et al. [12]	•N/A	•3D T1-weighted MRI obtained with a Signa 1.5 T MR imaging unit	•Transverse plane	•No significant association with post-surgical improvement reported. (Regression coefficient for total score, gait, cognitive, urinary subsections, TUG and MMSE was − 0.20, − 0.07, 0.01, -0.14, 0.53, 0.10 respectively *p* > 0.1)
Virhammar et al. [60]	*n* = 108	•Graded 0–3 •0 = no lesions •1 = punctate foci •2 = beginning of confluence of foci •3 = large confluent areas	•N/A	•T2- FLAIR MRI. (9% of patients on 3 T scanner; 70% on a 1.5 T scanner, 14% on a 1 T scanner and 7% on a 0.5 T scanner	•Transverse plane in center of 3rd ventricle in AP direction	•*OR* between SR and SNR: 0.75 (0.42–1.33), *p* = 0.33) was statistically insignificant. There was no statistical difference in outcomes between less severe and severe DWMH
Hong et al. [20]	*n* = 31	•••Measured using Fazekas et al. [12] ordinal scale from 0–3	•N/A	•3.0 Tesla MRI, T2-FLAIR	•Transverse	•There was no significant difference between SR and SNR within each grade (*p* = 0.054). Grade 0 had 2 SR and 3 SNR, Grade 1 had 15 SR and 6 SNR, Grade 2 had 0 SR and 3 SNR and grade 3 had 0 SR and 1 SNR. Univariate analysis showed an *OR*: 0.00 (95% *CI*: 0.974) was insignificant at *p* = 0.999

Studies included assessing the use of any advanced imaging radiological marker as predictor of shunt responsiveness. *SR*, shunt response; *S-NR*, shunt non-response; *FLAIR*, fluid-attenuated inversion recovery; *TUG*, timed up and go test; *MMSE*, mini mental state examination; *DWMH*, deep white matter hyperintensity; *CI*, confidence interval

**Table 15:** The use of lacunae for prediction of shunt response in iNPH

Study	Sample size	Radiological methodology	Cutoff specification	Image specification	Image plane	Main reported outcomes
Hong et al. [20]	*n* = 31	•Manually counted by blinded neurologist	•Not specified	•3.0 Tesla MRI scanner was used to gain Axial fluid-attenuated inversion recovery (FLAIR), T2- weighted images	•Not specified	•There was a significant difference between SR (mean lacunae 0.1 ± 0.2) and SNR (mean: 1.1 ± 1.4) *p* = 0.009. Univariate analysis showed an *OR*: 0.161 •(95% *CI*: 0.021–1.269) was insignificant at *p* = 0.083. Multivariate analysis: *OR*: 0.000, *p* = 0.098
Murakami et al. [39]	*n* = 24	•Not specified	•Absence of lacunae	•Regional cerebral blood flow analysis through N-isopropyl-p-[123I] iodoamphetamine (IMP) enhanced Single-photon emission computed tomography (SPECT)	•Not specified	•Presence of lacunae was significantly associated with SNR (*p* = 0.0153). Sensitivity 71.4%, specificity:80%, PPV 83.3% and NPV 66.7%. TP 10, TN 8, FP 2, FN4

Studies included assessing the use of any advanced imaging radiological marker as predictor of shunt responsiveness. *SR*, shunt response; *S-NR*, shunt non-response; *FLAIR*, fluid-attenuated inversion recovery; *SPECT*, single-photon emission computerized tomography; *NPV*, negative predictive value; *PPV*, positive predictive value; *TP*, true positives; *FP*, false positives; *TG*, true negatives; *FN*, false negatives; *OR*, odds ratio; *CI*, confidence interval

**Table 16:** The use of miscellaneous radiological markers on MRI and CT for prediction of shunt response in iNPH

Study	Sample size	Radiological methodology	Cutoff specification	Image specification	Image plane	Main reported outcomes
McGirt et al. [34]	*n* = 132	•Corpus callosum distention	•N/A	•CT/MRI	•N/A	•30 (28%) of patients had distention of the corpus callosum. Univariate analysis showed a significant likelihood of shunt response in those with distention, RR:1.64 (95% *CI*: 1.05–2.58). However, this was no shown in multivariate analysis: RR: 1.38 (95% *CI*: 0.85–2.20)
Agerskov et al. [2]	*n* = 168	•Widening of anterior part of Interhemispheric fissure. Graded for 0–2	•N/A	•MRI 1.5 T. trans-axial T1-weighted images	•Axial slice	•There was no difference, in each grade, between SR and SNR. There was also no significant correlation between grade and composite score. (-0.20, -0.04, -0.04 for total, gait, and cognition respectively.)

Studies included assessing the use of any miscellaneous radiological markers on MRI or CT as predictors of shunt responsiveness. MRI studies are above the double solid lines, CT studies are below. *SR*, shunt response; *S-NR*, shunt non-response; *RR*, risk ratio; *CI*, confidence interval

**Table 17:** The use of DESH on MRI and CT for prediction of shunt response in iNPH

Study	Sample size	Radiological methodology	Cutoff specification	Image specification	Image plane	Main reported outcomes
Shinoda et al. [51]	*n* = 55	•DESH ventriculomegaly, dilated sylvian fissures, tight high convexity, acute callosal angle, and focal sulcal dilation. Combined to form DESH score	•N/A	•MRI (no other information given)	•Transverse: EI, Tight high convexity •Coronal: Fissure Dilatation –Tight High Convexity, acute callosal angle	•Inverse correlation between the DESH score and the rate of change in the mRS score post shunting (r = -0.749) Shunt responders had higher pre-operative DESH score (6.50 ± 2.0 vs 3.94 [SNR] ± 1.5; *p* < 0.001) •Secondary outcomes: For INPHGS improvement: DESH score 6.39 ± 1.76 vs 4.26 ± 1.69; *p* < 0.001), for MMSE (DESH score 6.63 ± 1.82 vs 5.09 ± 1.93; *p* = 0.010), for TMT-A (DESH score 6.32 ± 1.97 vs 5.13 ± 1.93; *p* = 0.042), and for TUG-t (DESH score 6.48 ± 1.81 vs 4.33 ± 1.59; *p* < 0.001)
Virhammar et al. [60]	*n* = 108	•DESH present if narrow sulci at high convexity and Sylvian fissure ordinal were graded ≥ 1	•N/A	•T2 Flair, T1-weighted MRI. (9% of patients on 3 T scanner; 70% on a 1.5 T scanner, 14% on a 1 T scanner and 7% on a 0.5 T scanner	•Transverse and coronal plane	•Normal Sylvian fissures was associated with greater: mRS results (*p* = 0.01), balance scale (P = 0.01), 10 m walk, and the walking backward test (*p* = 0.05) compared with dilated Sylvian fissures •*OR* for DESH and its components: Narrow sulci 1.43 [(0.83–2.46) *p* = 0.2]. Ordinal sylvian fissure 1.35 [(0.57–3.21) *p* = 0.5]. DESH: 2.78[(1.09–7.061, *p* = 0.032]
Garcia-Armengol et al. [14]	*n* = 89	•DESH present if disproportionate enlargement the inferior subarachnoid spaces and tight high-convexity subarachnoid spaces	•N/A	•MRI: spin-echo T1-weighted	•Coronal perpendicular to the anterior commissure	•SR patients were significantly more likely to have DESH than SNR (79.7% vs 20%, *p* < 0.001) •Sensitivity: 0.794, specificity: 0.808, PPV: 0.909, NPV: 0.618. positive likelihood ratio: 3.98, negative likelihood ratio: 0.25 and Youden index 0.60 •TP:51, TN:20 FP:13, FN:5
Hong et al. [20]	*n* = 31	•narrowing of high cortical convexity sulci despite the widened Sylvian fissure	•N/A	•3.0 Tesla MRI scanner was used to gain Axial fluid-attenuated inversion recovery (FLAIR), T2- weighted images, T1-weighted images, and coronal T1-weighted images	•Coronal section	•Positive DESH finding in 13/14 SR and 6/12 SNR (*p* = 0.026) •Univariate analysis: DESH positivity had *OR* of 15.167 [(1.509–152.461 95% *CI*) *p* = 0.021]. On multivariate logistic regression analysis: DESH positivity had *OR* of 6.500 [(0.460–91.924 95% *CI*), *p* = 0.166)]
Agerskov et al. [2]	*n* = 168	•Ordinal rating 1 or 2 in Sylvian fissure dilation with obliterated sulci at the high convexity	•N/A	•MRI 1.5 T. The imaging protocol: 1) a sagittal T1-weighted volume sequence, 2) a trans axial FLAIR sequence, 3) a flow-sensitive sagittal TSE sequence, 4) an aqueduct-centered turbo field echo sequence	•Transverse and coronal images	•There was no difference in DESH findings between SR (present in 36%) and SNR (present in 34%) and it could not be used to predict SR in multivariate logistical analysis •TP:42, TN:35, FP:18, TN:73 •Its non-significant correlation coefficient with the composite score was 0.11
Grahnke et al. [16]	*n* = 72	•N/A	•N/A	•CT or MRI	•N/A	•No significant difference found between SR and SNR. DESH pattern found in 9 SR (20%) and in 7 SNR (26%) *p* = 0.55 •*OR* 19.250; 95% *CI*: 1.768–209.546; *p* = 0.015

Studies included assessing the use of any MRI or CT DESH as predictor of shunt responsiveness. MRI studies are above the double solid lines, CT studies are below. *SR*, shunt response; *S-NR*, shunt non-response; *DESH*, disproportionately enlarged subarachnoid space hydrocephalus; *FLAIR*, fluid-attenuated inversion recovery; *TSE*, turbo spin echo; *iNPHGS*, idiopathic normal-pressure hydrocephalus grading scale; *TMT-A*, trail making test A; *NPV*, negative predictive value; *PPV*, positive predictive value; *TP*, true positives; *FP*, false positives; *TG*, true negatives; *FN*, false negatives; *CI*, confidence interval

**Table 18:** The use CTC for prediction of shunt response in iNPH

Study	Sample size	Radiological methodology	Cutoff level	Contrast used	Time of CT	Main reported outcomes
Kazui et al. [27]	*n* = 100	•CTC score: 0–3 depending on stasis and density of contrast in ventricle compared to surrounding brain parenchyma 0 = density in the CSF space is same as in baseline CT scan •Score = 1, density is between 0 and 2 •Score = 2, density is same as the brain parenchyma in baseline CT scan •Score = 3, density is higher than brain parenchyma in baseline CT scan •Lateral ventricles, Sylvian fissure, and parietal sulci	•Low score (0–1)	•iohexol (Omnipaque®: 180 mg/ml)	•0, 6, 24 and 48 h after injection	•Parietal sulci after 48 h- *OR*: 0.47 (95% *CI*: 0.25–0.88) *p* = 0.02 for disappearance of urinary symptoms •CTC score Mean 1.4, SD:1.0
Kawaguchi et al. [26]	*n* = 100	•CTC score. Stasis of the contrast medium at the lateral ventricles (positive ventricular stasis) and at the Sylvian fissure or the parietal sulci (positive surface stasis)	•High score (indicated more stasis)	•iohexol (Omnipaque®: 180 mg/ml)	•0, 6, 24 and 48 h after injection	•CTC success rate: 85.4% •Ventricular stasis: sensitivity: 0.867, Specificity: 0.20, PPV:0.867, NPV: 0.2 •Surface stasis: sensitivity 0.817, specificity: 0.00, PPV:0.831, NPV 0 •Overall CTC (positive surface AND ventricular stasis): sensitivity 0.95, specificity 0, PPV: 0.851, NPV: 0. TP57, TN0, FP10, FN33
Black [5]	*n* = 62	•Images were graded as having delayed isotope clearance and failure of convexity ascent, mixed pattern or normal	•N/A	•Not available	•72 h	•11 (33%) of patients had ventricular entry, absence of convexity flow and delayed clearance, of these 73% improved. Normal pattern was seen in 9 with a 55% improvement. Mixed pattern was seen 13 with a 31% improvement rate. No differences were significant •TP: 9, TN: 13, FP:3, FN:9. Sensitivity: 47.1%, specificity: 81.3%, PPV: 72.7%, NPV:59.1%

Studies included assessing the use of any CTC radiological markers as predictor of shunt responsiveness. *SR*, shunt response; *S-NR*, shunt non-response; *CTC*, computerized tomographic cisternography *OR*, odds ratio; *CI*, confidence interval; *SD*, standard deviation; *NPV*, negative predictive value; *PPV*, positive predictive value; *TP*, true positives; *FP*, false positives; *TG*, true negatives; *FN*, false negatives

**Table 19:** The use cerebral blood flow for prediction of shunt response in iNPH

Study	Sample size	Radiological methodology	Cutoff level	Imaging technique	Main reported outcomes
Yamada et al. [62]	*n* = 25	•Regional CBF of 12 paired segments	•CBF Improvement of < 20% post acetazolamide injection	•SPECT. Following technetium-99 m-L, L-ethylcysteinate dimer injection, the 3DSRT method was used. Baseline CBF then post acetazolamide injection	•Sensitivity of 1.00, specificity of 0.60. TP:22 •TN:2, FP:1, FN:0
Ishii et al. [23]	*n* = 84	•Changes in regional CBF post injection: anterior-dominant CBF reduction type (A type), posterior-dominant CBF reduction type (P type), and mixed or diffuse CBF reduction type (M type)	•N/A	•SPECT. Imaging at baseline then Following technetium-99 m- injection. The 3DSPP method was used to analyze data	•PPV—A type: 0.83, P type: 0.9, M type 0.84
Kazui et al. [27]	*n* = 100	•Changes in regional CBF post injection: anterior-dominant CBF reduction type (A type), posterior-dominant CBF reduction type (P type), and mixed or diffuse CBF reduction type (M type)	•N/A	•SPECT. Imaging at baseline then Following technetium-99 m- injection. The 3DSPP method was used to analyze data	•The only statistically significant association was M type—*OR*: 0.26 (0.07–0.89) *p* = 0.03 for disappearance of urinary symptoms
Murakami et al. [39]	*n* = 24	Regional CBF	•N/A	•SPECT. Baseline CBF using 3D-SPP, imaging began 20 min after N-isopropyl-p-[123I] iodoamphetamine (IMP)	•Responders have reduced CBF in frontal base and the anterior part of limbic areas (cingulate gyrus). No *p*-values
Chen et al. [8]	*n* = 28	•CT Xenon CBF measurement. ACT challenge CT for cerebrovascular reactivity capacity (CRC) and CBF	•N/A	•rCBF: for 2 patients: 3 min inhalation protocol (30% xenon). For 26 patients 4 min (26% xenon) wash-in then 5 min washout protocol. Average rCBF: average of 2 measurements each at the anterior, middle and posterior centrum semiovale •CBF post ACT challenge also measured •CRC: scan 15–20 min post ACT challenge of 17 mg/Kg •For both, 4 contiguous slices at basal ganglia level to level of centrum semioval (CSWM)	•rCBF: There was no significant difference between SR (15.3 mL/min per 100 g (SD: 3.7) and SNR (17.9 mL/min per 100 g (SD: 3.8) •Post ACT-challenge: Significant difference between SNR (14.2mLper 100 g/min) vs SR (24.1 mL per 100 g/min) *p* = 0.008 •CRC: Average CRC could not alone be predictor of SR. Although CRC > 20% at the anterior area of CSWM was significantly different (SNR 1.06% vs SR 1.41%) *p* = 0.03 •There was strong positive correlation between the NPH scale and average rCBF (*p* < 0.02), average ACT challenge rCBF (*p* < 0.05), and CRC (*p* < 0.03)
Ziegelitz et al., 2014 [64]	*n* = 22	•CBF and Cerebral blood volume (calculated by Ostergaard et al. method). These were mapped onto FLAIR images to find rCBF in 15 anatomical locations of the brain	•basal, medial frontal > 0.798	•Dynamic susceptibility contrast (DSC) MRI perfusion study using 1.5 T Gyro-scan for a k-space gradient-echo echoplanar imaging (EPI) technique with 0.1 mmol/kg gadolinium-labeled diethylenetriaminepentaacetic acid bolus. ROI were taken from FLAIR images	•There were significant negative correlations between rCBF along the white matter profile (measured at 4 distances from ventricular wall) and improvement in NPH score. (0 mm: -0.448, 2 mm: -0.629, 5 mm: -0.616*7 mm: -0.564). (*p* < 0.05) •There was a significant negative correlation between rCBF in GM and degree of improvement in the shunt response group. (rho = -0.541, *p* < 0.05). PVWM rCBF did not significantly correlate with improvement in NPH score •SR had higher rCBF values in the basal medial frontal cortex than SNR (*p* = 0.019). ROC analysis resulted in AUC < 0.854, using a cutoff of > 0.798 has a sensitivity of 80% and specificity of 100% •TP:12, TN:6, FP: 0, FN: 3
Agerskov et al. [3]	*n* = 20	•Relative CBF and cerebral blood volume in 6 regions; 3 within upper part of the mesencephalon and 3 located 6 mm caudally in the pons. NB Relative to blood flow to occipital lobe •Mean transit time: CBV/CBF	•N/A	•1.5 T Gyroscan MRI. Dynamic susceptibility contrast using segmented k-space EPI technique was used to assess perfusion with a 5 ml/s bolus of 0.1 mmol/kg Gd-DTPA. Transverse FLAIR sequence was used to draw 6 ROIs across two regions. Perfusion estimates were calculated using arterial input function	•There was no significant relation between pre-operative rCBF and shunt response: •Mesencephalon: SR: 0.86 (IQR 0.77–0.93); SNR: 0.90 (IQR 0.74–1.02) •Pons: SR 0.69 (IQR: 0.61–0.77); SNR: 0.77 (IQR: 0.68–0.94) •There was no significant relation between MTT or CBV with shunt outcome •NB. There was an increase in rCBF in SR group in mesencephalon and pons but in SNR there was a decrease in the mesencephalon

Studies included assessing the use of any SPECT radiological markers as predictor of shunt responsiveness. *SR*, shunt response; *S-NR*, shunt non-response; *CBF*, cerebral blood flow; *CBV*, cerebral blood volume; *CRC*, cerebrovascular reactivity capacity; *CSWM*, centrum semiovale white matter; *ACT*, acetazolamide; *AUC*, area under the curve; *MTT*, mean transit time; *FLAIR*, fluid-attenuated inversion recovery; *SPECT*, single-photon emission computerized tomography; *ROI*, regions of interest; *NPV*, negative predictive value; *PPV*, positive predictive value; *TP*, true positives; *FP*, false positives; *TG*, true negatives; *FN*, false negatives

**Table 20:** The use of novel imaging techniques for prediction of shunt response in iNPH

Study	Sample size	Radiological methodology	Cutoff level	Image specification	Image plane	Main reported outcomes
Aoki et al. [4]	*n* = 34	•Exact-low-resolution-brain-electromagnetic-tomography. A type of approach to EEG which can localize electrical activity. Normalized power variance (NPV) calculated	•Prediction score = log (beta eLORETA-NPV) + 1.49 •If number is positive, it predicts patient will respond to shunt	•19-electrode EEG system •Analysis was computed for five frequency bands in high convexity areas.: •delta (1.5–4.0 Hz), •theta (4.5–7.0 Hz), •alpha (7.5–13.0 Hz), •beta (13.5–29.5 Hz), •gamma (30.0–59.5 Hz)	•N/A	•positive predictive value of 61.1% (11/18) and negative predictive value of 75.0% (12/16)
Jurcoane et al. [25]	*n* = 12	•Fractional anisotropy, mean diffusivity, radial diffusivity, axial diffusivity and magnetization transfer ratio	•a decrease of > 1% in axial diffusivity	•Diffuse tensor imaging using echo-planar sequence	•Corticospinal tract and the superior lateral fascicle	•Decrease of > 1% in axial diffusivity could differentiate between SR and SNR with a sensitivity of 87.5% and a specificity of 75% to predict SR
Chen et al. [8]	*n* = 28	•Magnetic resonance spectroscopy (MRS): N-acetyl aspartate/ creatine ratio change at the anterior, middle, and posterior Centrum Semiovale white matter (CSWM) in both sides. Average was taken of those 6	•N/A	•1.5-T MRI. T1/T2 and FLAIR in axial section. MRS using the with default chemical shift imaging method	•Transverse, sagittal and coronal	•NAA/Cre strongly correlated with rCBF (*p* < 0.001) and scores < 1.5 in anterior CSWM were found in all 23 patients with more than gait symptoms •NAA/Cre not significantly correlated with CRC (*p* = 0.635) •There was no significant correlation with clinical response, although in the 5 SNR, NAA/Cre was < 0.8 in at least 2 regions •There was a weak correlation between NAA/Cre and the NPH scale (R2 = 0.332, *p* < 0.1)
Agerskov et al. [3]	*n* = 20	•Apparent diffusion coefficients in 6 regions; 3 within upper part of the mesencephalon and 3 located 6 mm caudally in the pons	•N/A	•1.5 T Gyroscan MRI. ADC maps calculated using Transverse DWI. FLAIR sequence was used to draw 6 ROIs in two regions to map the ADC onto	•All sequences angulated parallel to the callosal plane	•There was no significant difference in ADC between SR and SNR groups •Mesencephalon: SR: 800 (IQR 750–821); SNR: 775 (IQR 753–828) •Pons: SR: 772 (IQR 738–813); SNR: 760 (IQR 721–798) •NB there was a post-operative increase in ADC in the mesencephalon and pons in responders
Wu et al. [61]	*n* = 41	•Volumetric analysis using Automatic whole-brain segmentation. Brain split into 283 ROIs. 5 levels of granularity were produced, depending on number of ROIs in each image. 7, 19, 54, 137, and 283 for levels 1–5 respectively	•N/A	•High-resolution T1-weighted MRI using MPRAGE sequence. Multi-atlas algorithm was used to segment the brain	•Sagittal	•Level 5 could not be used for statistical reasons •The correlation between the predictive model at each level and the ground truth for the Tinetti score were r = 0.55, 0.56, 0.76, and 0.80 at levels 1, 2, 3, and 4. For MMSE it was *r* = 0.85, 0.86, 0.87, and 0.88 at levels 1, 2, 3, and 4, respectively. When the predictive model only had the volumetric analysis as input, the *r* values at levels 1–4 were 0.53, 0.53, 0.77, and 0.75 for Tinetti and 0.61, 0.75, 0.69, and 0.83 for MMSE •Areas strongly which related to Tinetti score were ventricle and sulci, especially right parietal and frontal sulci and bilateral inferior left ventricle (a strong predictor) •Areas which strongly related to MMSE score were cortical gyri and white matter e.g., left angular gyrus, right cuneus, left fornix/stria terminalis, and left anterior deep white matter
Kuchcinski et al. [31]	*n* = 38	•Automated sulcal morphometry used to assess the size of the sulcal opening of 10 sulci. Two ratios between 4 different sulci were also calculated: Lateral/intraparietal and Calcarine/cingulate	•N/A	•3 T MR scanner Magnetization-Prepared Rapid Gradient-Echo 3D T1 sequence. Morphometry was performed using BrainVISA software	•Sagittal	•The lateral and superior temporal sulci were significantly correlated with score improvement r = 0.42, *p*-0.02; r = 0.38, *p* = 0.03 respectively •NB interesting when used to compare with population (healthy controls and cognitive dementia caused by vascular accidents, calcarine/cingulate ratio (AUC = 0.94; 95% *CI*: 0.89,0.99) was the most discriminative sulci, and a cutoff of 0.95 had sensitivity 96.8% and specificity 83.3%

Studies included assessing the use of any advanced imaging radiological marker as predictor of shunt responsiveness. *SR*, shunt response; *S-NR*, shunt non-response; *ADC*, apparent diffusion coefficients; *CBF*, cerebral blood flow; *CBV*, cerebral blood volume; *CRC*, cerebrovascular reactivity capacity; *CSWM*, centrum semiovale white matter; *EEG*, electroencephalogram; *ACT*, acetazolamide; *AUC*, area under the curve; *MTT*, mean transit time; *MRS*, magnetic resonance spectroscopy; *NAA*, N-acetyl aspartate; *CRE*, creatinine; *FLAIR*, fluid-attenuated inversion recovery; *SPECT*, single-photon emission computerized tomography; *ROI*, regions of interest; *IQR*, interquartile range; *NPV*, negative predictive value; *PPV*, positive predictive value; *TP*, true positives; *FP*, false positives; *TG*, true negatives; *FN*, false negatives

**Table 21:** The use of intracranial volume for prediction of shunt response in iNPH

Study	Sample size	Radiological methodology	Cutoff level	Image specification	Image plane	Main reported outcomes
Palm et al. [42]	*n* = 26	•4 variables were obtained: intracranial volume (parenchyma and CSF), total brain volume, ventricular CSF volume lateral, third, and fourth ventricles), and extra ventricular CSF volume. The latter 3 were used as a ratio to total intracranial volume	•N/A	•Dual spin-echo (proton attenuation and T2-weighted) images.0.5 T for 7 (26.9%) or 1.5 T for 19 (73.1%)	•N/A	•There was no significant difference between SR and SNR in any 4 of the variables obtained
Yamamoto et al. [63]	*n* = 16	•Using voxel-based morphology to measure CSF areas. Measured volume of ventricles and sylvian fissures (vVS) and volume of sub-arachnoid space at the high/ midline convexity (HCM). Calculated vVS/HCM ratio as measure of brain deformation	•N/A	•1.5 T MRI with a T1-weighted gradient echo sequence	•Sagittal	•Pre-shunting, the vHCM and vVS were negatively correlated (r = –0.59, *p* = 0.01)

Studies included assessing the use of any advanced imaging radiological marker as predictor of shunt responsiveness. *SR*, shunt response; *S-NR*, shunt non-response; *CSF*, cerebrospinal fluid; *vVS*, volume of ventricles and sylvian fissures; *HCM*, high/midline convexity

### Study characteristics

**Fig. 4:**
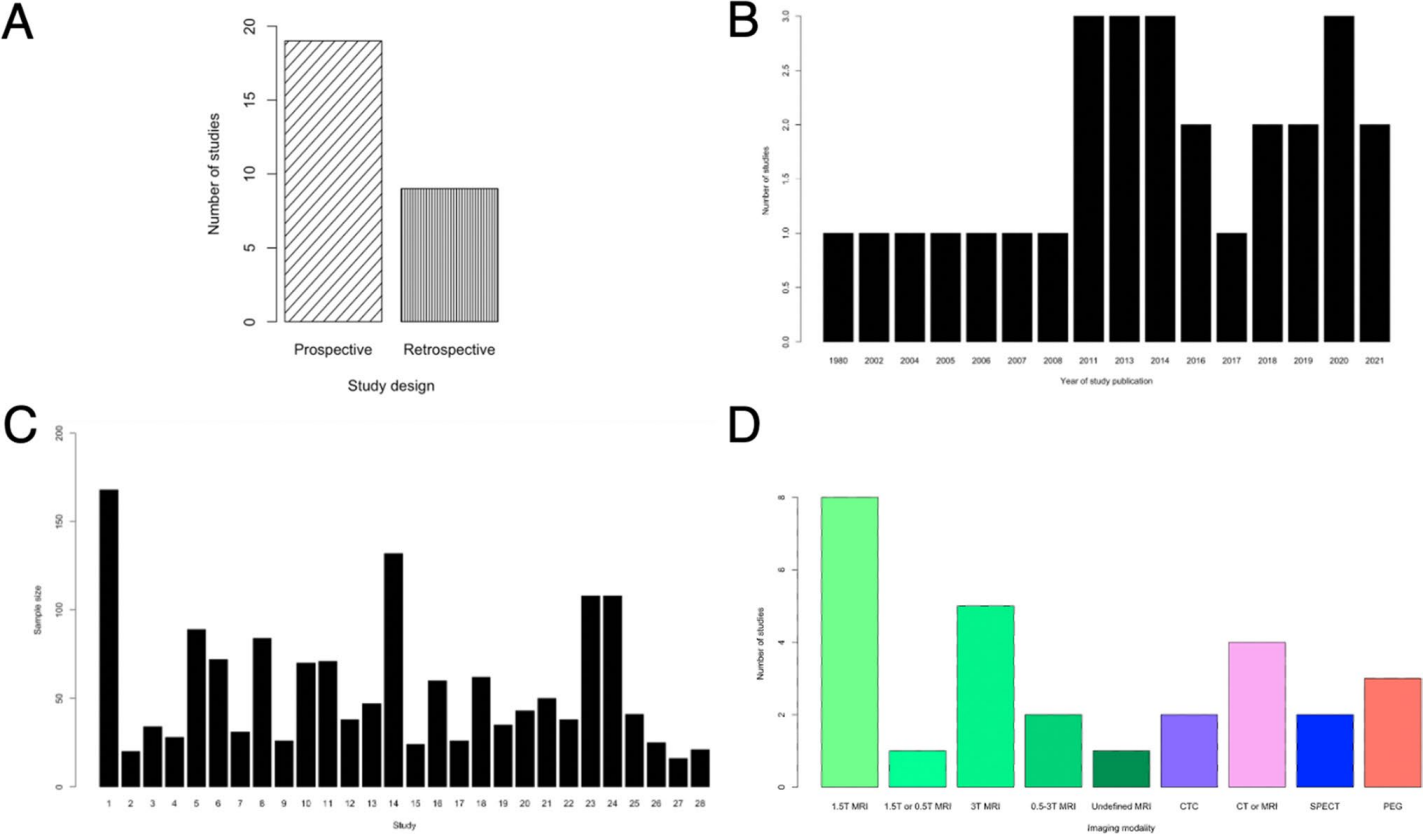
**A** A bar plot visualizes the number of prospective (*n* = 19) and retrospective (*n* = 9) studies included in the systematic review (*n* = 28) [2–5, 8, 14, 16, 20, 23, 25–27, 31, 33, 34, 39, 41, 42, 45–47, 49, 59–64]. **B** A bar plot displays the number of studies for the following years of publications: 1990 (*n* = 1), 2002 (*n* = 1), 2004 (*n* = 1), 2005 (*n* = 1), 2006 (*n* = 1), 2007 (*n* = 1), 2008 (*n* = 1), 2011 (*n* = 3), 2013 (*n* = 3), 2014 (*n* = 3), 2016 (*n* = 2), 2017 (*n* = 1), 2018 (*n* = 2), 2019 (*n* = 2), 2020 (*n* = 3), 2021 (*n* = 2). **C** A bar plot shows the sample size of each included study in the systematic review (*n* = 28). Studies are named numerically 1–28, each number refers to the cited studies in synchronized order [2]. **D** A bar plot visualizes the number of included studies (*n* = 28) [2–5, 8, 14, 16, 20, 23, 25–27, 31, 33, 34, 39, 41, 42, 45–47, 49, 59–64] that use each of the following imaging modalities: “1.5 T MRI” (*n* = 8), “1.5 T or 0.5 T MRI” (*n* = 1), “3 T MRI” (*n* = 5),”0.5-3 T MRI” (*n* = 2), “Undefined MRI” (*n* = 1), “CTC” (*n* = 2), “CT or MRI” (*n* = 4), “SPECT” (*n* = 2), “PEG” (*n* = 3). MRI, magnetic resonance imaging; CT, computed tomography; CTC, computerized tomographic cisternography; SPECT, single-photon emission computerized tomography; PEG, pneumoencephalogram

All studies included in the systematic review (*n* = 28) are characterized in Fig. 4. Firstly, Fig. 4A demonstrates that the majority of papers (19/28) had a prospective study design [2–4, 8, 14, 20, 23, 25–27, 31, 33, 34, 39, 40, 45, 46, 62, 64], while the remaining (9/28) were retrospective [5, 14, 41, 47, 49, 59, 59–61]. The publication dates ranged between 1980 and 2021, with 27/28 studies published over the last 20 years and 20/28 studies published after 2010 (Fig. 4B). Sample sizes ranged from 12 to 168 and 12 studies had over 50 participants (Fig. 4C). Figure 4D compares the different imaging modalities used by the included studies. The majority employed MRI; eight used 1.5 T MRI, five used 3 T MRI, one used a combination of the two while two studies reported the use of “0.5-3 T” MRI, and one did not report MRI specifications (Fig. 4D). Four papers used both CT and MRI, two used computerized tomography cisternography (CTC), two used SPECT, and three used pneumoencephalogram (Fig. 4D).

#### Measurement of shunt response

The literature refers to several ways of characterising iNPH patients as shunt responders or shunt non-responders (Table 1). All studies used some form of scoring system measuring improving in gait, cognition urinary symptoms or a combination of the three, in esnnsence an improvement in these domains lead to a label of “shunt responder”, however the degree of improvement and method of testing this improvement differed among studies. Table 1 contains an in-depth summary of the methodology of each studies’ criteria for shunt response. Seven studies used the NPH grading scale [9, 18, 19, 36, 54, 60, 62], while 9 used all three of gait, urinary and cognitive symptoms assessed on separate scales [4, 15, 23, 24, 30, 40, 52, 60, 65], including the use of MMSE [6, 24, 29, 36, 62, 65] and Tinetti scales. Five used the modified Rankin score [8, 12, 18, 26, 27]. Palm et al. [42] used their own grading scale, while Black [30] employed Steins and Langfitts [30] scale to assess shunt response. 

### Patient characteristics

**Fig. 5:**
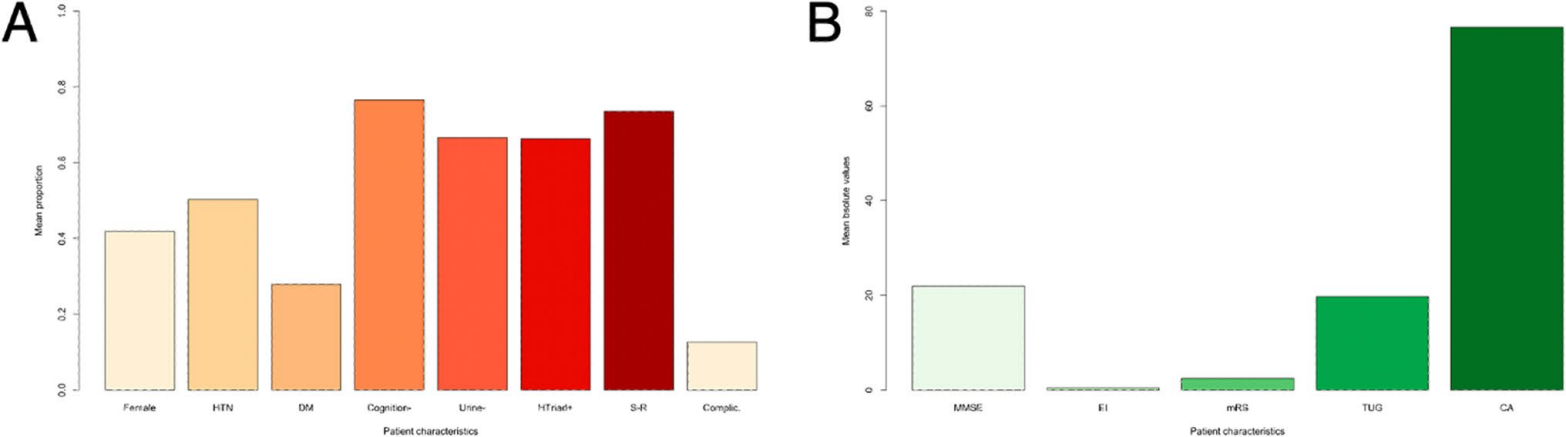
**A** A bar plot visualizes the mean total proportion (0–1) of the following patient characteristics (minimum of 7 studies have to report on it) among all included studies in the systematic review (*n* = 28), in the following order and converted into percentages and rounded to zero decimals: proportion of patient sample being female (“Female”, 42%, *n* = 28), pre-existing arterial hypertension (“HTN”, 50%, *n* = 11), pre-existing diabetes mellitus (“DM”, 28%, *n* = 7), cognitive deficits (“Cognition- “, 77%, *n* = 13), urinary dysfunction (“Urine- “, 67%, *n* = 13), patient presenting with the Hakim triad clinically (“HTriad + ”, 63%, *n* = 7), mean proportion of patients being shunt-responsive (“S-R”, 74%, *n* = 7) and proportion of complications (“Complic.”, 13%, *n* = 16). **B** A bar plot visualizes the mean absolute values (0–1) of the following patient characteristics (minimum of 7 studies have to report on it)among all included studies in the systematic review (*n* = 28), in the following order and rounded to two decimals: mean MMSE score (“MMSE”, 21.9, *n* = 16), mean Evan’s index result (“EI”, 0.373, *n* = 13), modified Rankin scale (“mRS”, 2.43, *n* = 7), timed-up-and-go test (“TUG”, 19.7 s, *n* = 8) and colossal angle (“CA”, 76.6°, *n* = 8)

Figure 5 displays the iNPH patient characteristics of the studies reporting on this subject (minimum seven studies had to report on the subject to be included in our qualitative analysis). About 41.8% of participants were female (reported in 27/28 studies). A total of 77.6% of participants presented with reduced cognition (reported in 13/28) and 66.6% with urinary symptoms (reported in 7/28); overall, 66.3% presented with all three of Hakims triad (reported in 7/28). Participants also presented with the following co-morbidities, 27.9% had diabetes mellitus (reported in 7/28), and 50.3% had hypertension (reported in 11/28) (Fig. 5A). As illustrated in Fig. 5B, the mean MMSE score was 21.9 (reported in 16/28), mean EI was 0.373 (reported in 13/28), mRS 2.53 (reported in 7/28), TUG 19.7 s (reported in 8/28), and the CA was 76.6° (reported in 8/28). Overall, 73.6% of participants responded well to shunting (reported in all 28 studies) and complications were seen in 12.7% (reported in 16/28).

### Overall trends and patterns

**Fig. 6:**
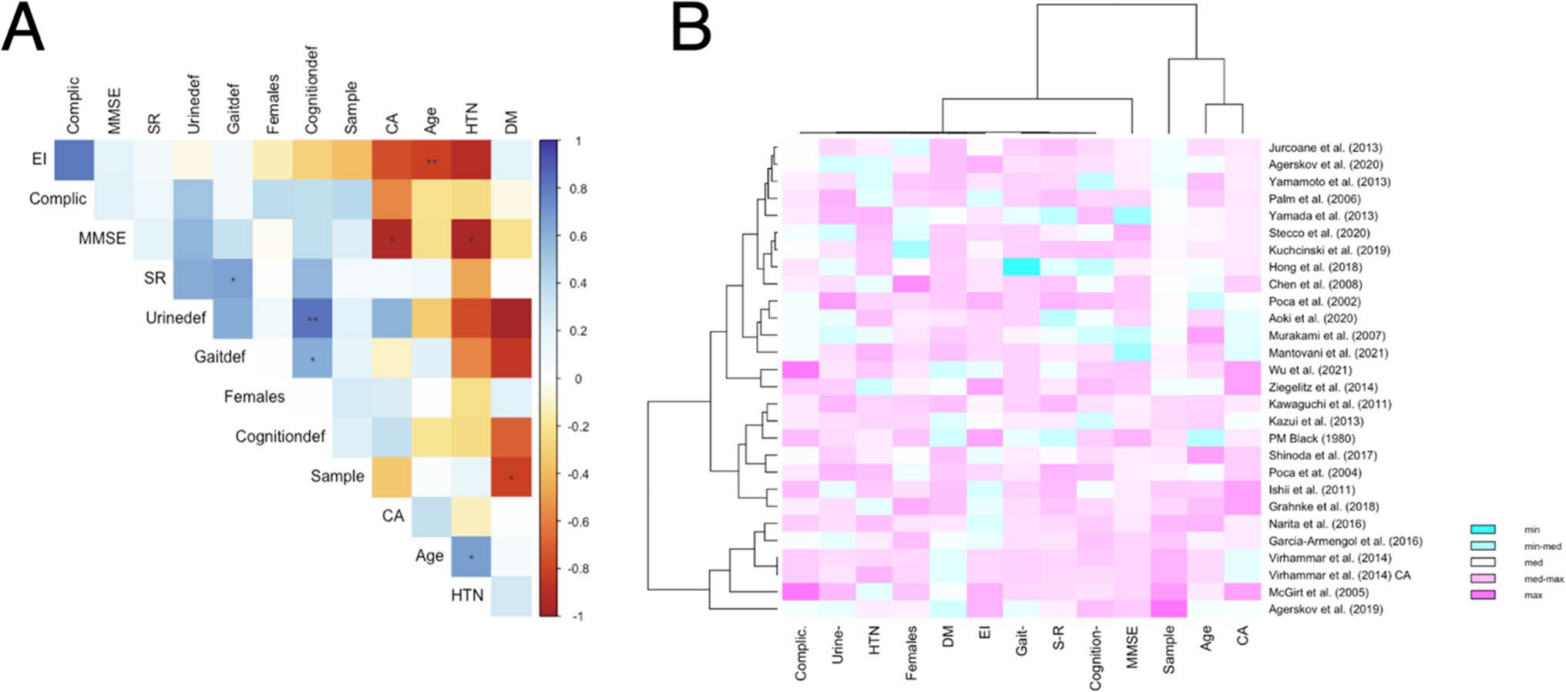
**A** A correlation matrix visualizes the relationships of following parameters among all studies included in the systematic review (*n* = 28): The following parameters are used here: Patient sample size (“Sample”), mean age of the patients (“Age”), proportion of patient sample being female (“Females”), pre-existing diabetes mellitus (“DM”), pre-existing arterial hypertension (“HTN”), gait deficits (“Gait- “ or “Gaitdef”), cognitive deficits (“Cognition- “ or “Cognitiondef”), urinary dysfunction (“Urine- “ or “Urinedef”), mean proportion of patients being shunt-responsive (“S-R”), and proportion of complications (“Complic.” or “Complic”), mean patient scores on the Mini Mental State Exam (“MMSE”). Furthermore, mean patient scores for Evan’s Index (“EI”) and mean values for Callosal Angle (“CA”). The legend bar at the right of the matrix explains the coloring. One asterisk (*) indicates a statistical significance of *p* < 0.05, two asterisks (**) indicate *p* < 0.01, three asterisks (***) indicate *p* < 0.001. **B** A heatmap based on machine learning imputation visualizes and compares the occurrence of the same parameters as **A** among all studies included in the systematic review (*n* = 28). The respective legend is shown at the bottom right corner. Turquoise coloring indicates minimum values (“min”), white coloring indicates medium values, and pink coloring indicated maximum values. Clustering is shown above and to the left of the graph

**Fig. 7:**
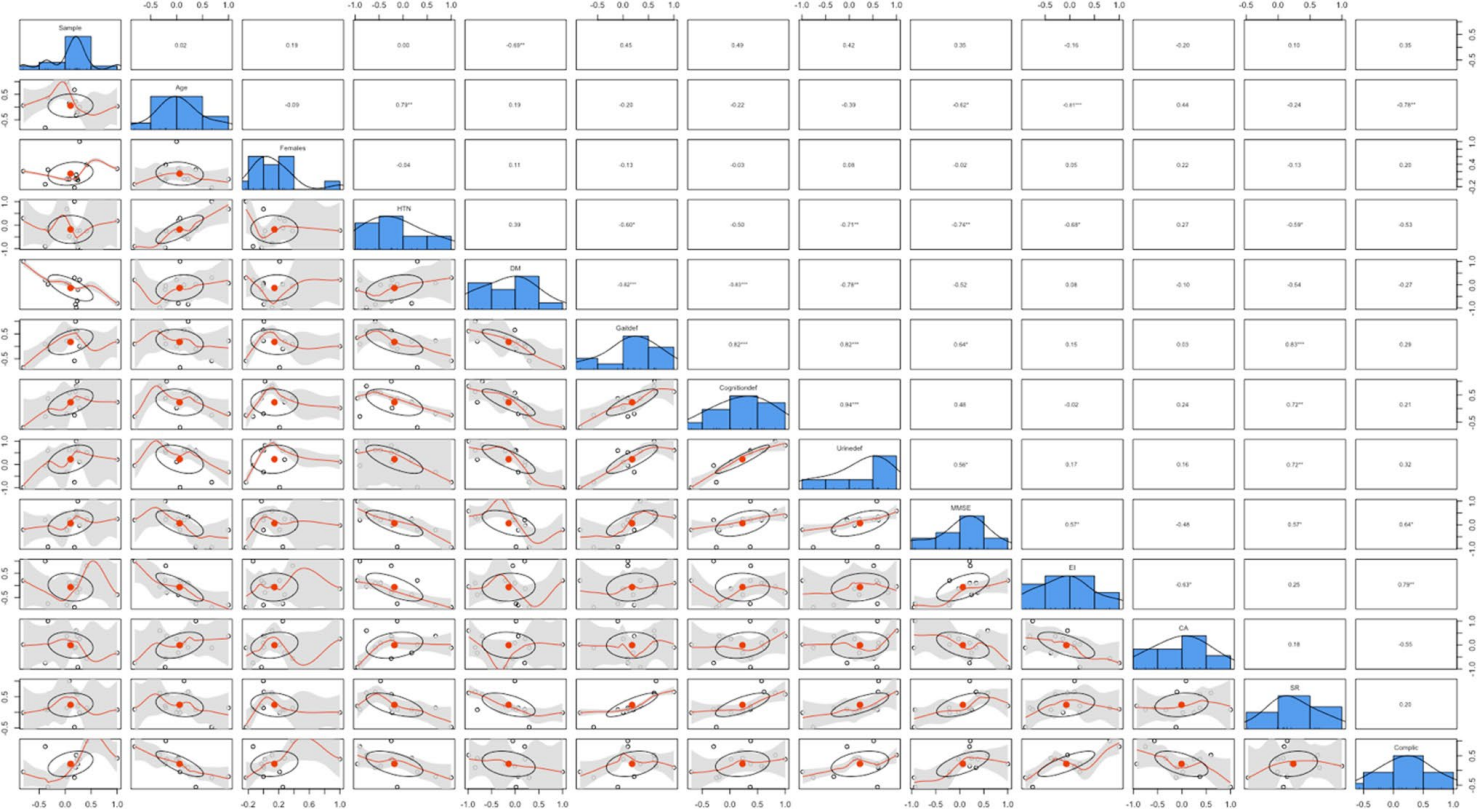
A scatter plot of matrices (SPLOM), with bivariate scatter plots is shown: below the diagonal, histograms on the diagonal, and the Pearson correlation above the diagonal. One asterisk (*) indicates a statistical significance of *p* < 0.05, two asterisks (**) indicate *p* < 0.01, three asterisks (***) indicate *p* < 0.001. A correlation analysis is run for following variables for all included studies in the systematic review (*n* = 28): Patient sample size (“Sample”), mean age of the patients (“Age”), proportion of patient sample being female (“Females”), pre-existing diabetes mellitus (“DM”), pre-existing arterial hypertension (“HTN”), gait deficits (“Gaitdef”), cognitive deficits (“Cognitiondef”), urinary dysfunction (“Urinedef”), mean proportion of patients being shunt-responsive (“S-R”), and proportion of complications (“Complic”), mean patient scores on the Mini Mental State Exam (“MMSE”). Furthermore, mean patient scores for Evan’s Index (“EI”) and mean values for Callosal Angle (“CA”). The red line is the locally estimated scatterplot smoothing (LOASS) line, and the ellipse is the correlation estimate ellipse. And the shaded area is the confidence interval area of the LOASS line

In Fig. 6A–B, a correlation matrix and a heatmap visualize and compare the occurrence of all numerical study characteristics and patient characteristics. The multivariate correlation matrix (Fig. 6A) visualizes the relationship between different relevant parameters from all 28 studies included in the systematic review. The main significant negative correlation found are as follows: age and EI are negatively correlated with high significance (*p* < 0.0.1), arterial hypertension and sample size are negatively correlated with significance (*p* < 0.05), callosal angle and MMSE are negatively correlated with significance (*p* < 0.05). The main significant positive correlations found are as follows: Cognitive deficits are positively correlated with urinary deficits with high significance (*p* < 0.01), shunt response and gait deficits are positively correlated with significance (*p* < 0.05), and cognitive deficits and gait deficits are positively correlated with significance (*p* < 0.05), and age and arterial hypertension are positively correlation with significance (*p* < 0.05). To ensure, that these findings were not skewed by missing values, a machine learning–based correlation matrix was employed for the same parameters and data as Fig. 6A, but missing values were imputed (Supplementary Material: Fig. 1). The machine learning–based correlation matrix consolidated the findings of the non-imputed matrix in general; most importantly, it also produced a very highly significant (*p* < 0.001) negative correlation between age and EI. Next, a SPOM (Fig. 7) was employed, to assess bivariate correlation. It also consolidated the findings of the initial multivariate correlation analysis in Fig. 6A, most importantly showing a negative correlation of EI and age with very high significance (*p* < 0.001) at Pearson correlation of (− 0.81). Finally, a scatterplot matrix was computed to show pair-wise univariate correlation including combined depiction of a linear regression line and LOASS line (Supplementary Material: Fig. 2). This scatterplot matrix also consolidated the findings of all previous correlation matrices. Importantly, it showed a low spread of the data for the EI and age correlation, with the linear regression line and LOASS being almost identical, which add to the robustness of this finding. Figure 6B is a machine learning–based heatmap, which visualizes and compares the occurrence of the same parameters presented in Fig. 6A among all 28 studies included in the systematic review. This shows a trend that studies with a smaller sample size tend to report less complications, which include less females, more patients with diabetes mellitus, less shunt responders, and less patients with cognitive deficits.

### Radscale markers

The widely used and cited Radscale consists of the following radiological markers: Evan’s index, callosal angle, size of temporal horns, narrow high-convexity sulci, dilated Sylvian fissures, focally dilated sulci, periventricular white matter changes and bulging of the lateral ventricular roof. Each one is reviewed individually below.

#### Evan’s Index

A total of 5 papers [2, 20, 41, 60, 61] investigated the use of Evan’s index in the prediction of SR in iNPH (Table 2). Measurements of EI were taken on T1-weighted MRI images in 3 studies [3, 41, 61]; in the other two [20, 60], the MRI sequence was not reported. The EI was defined as the ratio of the maximal diameter of the frontal horns of the lateral ventricles to the maximal inner diameter of the skull as measured on transverse sections in all papers but Wu et al. [61], where the definition was not given. Agerskov et al. [2] found all patients had an EI > 0.3; it could not be used to predict shunt response as there was no significant difference in Evan’s index between shunt responders (median 0.4) and shunt non-responders (median 0.39) (*p* > 0.05). This was also found by Hong et al. [20], and Virhammar et al. [60], the former reports no significant difference in mean EI between shunt responders (0.37 ± 0.04) and non-responders (0.37 ± 0.03) (*p* = 0.77). Narita et al. [41] reported no association between EI and post-shunt improvement, with no significant correlations reported between EI and post-shunt recovery. When Wu et al. [61] used a ML model to ascertain the usefulness of EI, the model’s prediction of MMSE and Tinetti score using EI alone correlated with the ground truth (actual MMSE and Tinetti score) at *r* = 0.42 and *r* = 0.6 respectively. However, when EI was used in conjunction with other features such as symptom severity, age, and sex in the model, its predictive accuracy increased to *r* = 0.48 for MMSE and *r* = 0.8 for Tinetti, highlighting its use in conjunction with other features.

#### Callosal angle

Eight papers investigated the use of callosal angle to predict shunt response in patients with iNPH [2, 5, 14, 20, 33, 41, 59, 60]. Six studies [2, 20, 33, 41, 59, 60] used MRI, Grahnke et al. [16] used both CT and MRI, while Black [5] used pneumoencephalograms. The plane of measurement was consistent in 6 of the 8 studies [2, 20, 33, 41, 59, 60]; perpendicular to the anterior–posterior commissure line. Black [5] used the AP projection of the pneumoencephalogram, while Grahnke et al. [16] used a mid-sagittal plane parallel to the floor of the 4th ventricle. The CA in 3 studies [20, 59, 60] was defined as the angle between the lateral ventricles on a coronal image; in 2 [20, 41], as the angle between the left and right corpus callosum; in 1 [5], as the angle of the junction of frontal horn roofs, while 2 [2, 33] did not state a definition. Three studies found no relation between callosal angle and shunt outcome. Hong et al. [20] found no significant difference (*p* = 0.109) in mean CA between responders (75.2°) and non-responders (88.3°); this was supported by the findings of Black [5], who also reported a cutoff of 120° had a sensitivity of 50% and specificity of 60%. Additionally, Agerskov et al. [2] found that it could not predict shunt outcome and it was not significantly correlated with outcome, reporting a median CA in responders of 68° and 69° in non-responders (*p* > 0.05). Two studies revealed mixed results, although Narita et al. [41] found significant associations between the presurgical callosal angle and MMSE (*B* =  − 0.04, *R*^2^ = 0.08, *p* = 0.035); they found no significant correlation between callosal angle and total INPH grading scale or TUG. Mantovani et al. [33] found that CA did not correlate with modified Rankin scale (mRs) or iNPH grading scale improvement and there was no difference in preoperative CA between shunt responders and non-responders, but a ROC analysis revealed a significant odds ratio (*OR*) of 2.15 (95% *CI* 1.03–4.52) when using a 59.5° cutoff. They found an alternative measurement, the anterior CA, (which is measured on the anterior commissure rather than the posterior commissure) to have a higher Youden index (0.344 vs 0.327 for CA), and there was a significant difference between shunt responders (98.3° ± 11.4°) and non-responders (108.6° ± 15.1°). Although the ACA again did not significantly correlate mRs or INPHGS outcome. A ROC analysis showed that a cutoff of 112° had an *OR* of 2.97 (95% *CI* 1.04–8.5). Three studies [14, 59, 60] report that CA has significant use in predicting shunt outcomes. Virhammar et al. [60] found a significant *OR* between responders and non-responders of 0.57 [(95% *CI*: 0.36–0.91), *p* = 0.017], and in a second paper, [59] calculated an *OR* of 0.97 [(95% *CI* 0.93–0.99), *p* < 0.05] and found CA to be significantly smaller (*p* > 0.05) in S-R (59° vs 68°). A cutoff of 63° had a sensitivity of 67% and specificity of 65%. These findings were shared by Grahnke et al. [16]: using a cutoff of 105.4° had a sensitivity of 41.5% and a specificity of 87%, the mean CA in responders was 108.4° compared to 117.6° in non-responders. It is worth noting the different planes used for CA measurement which may explain the vastly different angles between papers. The unadjusted *OR* was 0.96 [(95% *CI*: 0.93–0.998), *p* = 0.037] and they also found for every degree the CA was lower, a patient was 4% more likely to benefit from surgery.

#### Periventricular white matter changes

Six papers [2, 20, 34, 41, 46, 60] studied the effect of periventricular white matter (PVWM) changes on shunt outcome. Four studies [2, 20, 41, 60] used an ordinal scale, of which 2 used one developed by Fazekas et al. [12]. McGirt et al. [34] did not report a methodology of assessing PVWM changes on CT and MRI, while Poca et al. [46] noted lucencies in frontal and other locations using CT. The other 4 groups used MRI in the transverse plane. Five of 6 studies saw no relation between PVWM changes and shunt outcome. In univariate analysis, McGirt et al. [34], Virhammar et al. [60], and Hong et al. [20] all showed its insignificance and inability to predict shunt outcome. Narita et al. [41] found no significant (*p* > 0.1) association with post-surgical improvement and Agerskov et al. [2] saw no significant difference within each grade (0–3) of PVWH changes between responders and non-responders. However, Poca et al. [46] found that those with lucencies in frontal and other areas were significantly more likely to show improvement in the NPH scale, the memory, and orientation part of the Wechsler memory scale (WMS); one-way ANOVA = 7.56, *p* = 0.002, one-way ANOVA = 6.21, *p* = 0.006, and chi-square = 11.41, *p* = 0.003 respectively.

#### Dilated cortical sulci

Five studies [2, 5, 41, 46, 60] analyzed the outcome in patients who had an absence of dilated cortical sulci. Virhammar et al. [60] and Narita et al. [41] categorized focally enlarged cortical sulci as either present or absent, while Poca et al. [46] categorized sulci as normal, obliterated, or enlarged. Agerskov et al. [2] reported the effect of both focally enlarged sulci, numbered 0, 1, 2, > 2, and obliteration of high convexity sulci. Black [5] did not report a methodology but used pneumoencephalogram and CT, both Black and Poca et al. used CT. [46]; the rest used MRI. Agerskov et al. [2], Narita et al. [41], and Virhammar et al. [60] found no association between dilated sulci and outcome assessment scores. Black [5] found no difference in cortical size between responders and non-responders when using pneumoencephalograms (PEGs) and calculated a sensitivity and specificity of 66.7% and 35.7%. However, he did find a significant difference when using CT, with a sensitivity and specificity of 78.6% and 75.0% respectively. Poca et al. [46] saw those with enlarged sulci were less likely to improve in cognitive tests and reported a significant difference between groups in the information subset of WMS (chi-square = 10.05, *p* = 0.007).

#### Sylvian fissure size

Four studies [2, 41, 46, 60] evaluated how Sylvian fissure size is correlated with shunt outcome. Poca et al. [46], Agerskov et al. [2], and Narita et al. [41] used ordinal grading scales from narrowed to severely dilated, while Virhammar et al. [60] measured height in millimeters as well as an ordinal grade assessment. All but Poca et al. [46] used MRI. There were mixed reports of its significance. Poca et al. [46] reported those with normal fissures showed greater improvement in Trail Making Test B (chi-square test: 7.18, *p* = 0.007); however, Narita et al. [41] found the contrary; a significant correlation between Sylvian fissure dilation and change in iNPHGS gait domain (*B* = 0.59, *R*^2^ = 0.08, *p* = 0.02), but no significant associations with other outcome measures. Both Agerskov et al. [2] and Virhammar et al. [60] found no difference in sylvian fissure size between responders and non-responders.

#### Temporal horn size

Three studies [2, 46, 60] investigated temporal horn size. Poca et al. [46] categorized them into either normal or enlarged on CT, while Agerskov et al. [2] and Virhammar et al. [60] measured the maximum diameter on MRI. The former 2 report no effect of size on shunt outcome (*p* > 0.05), and the difference in size between responders and non-responders was 0.1 mm (9.0 mm vs 9.1 mm respectively) [2]. However, Virhammar et al. [60] found a significant difference between responders and non-responders and calculated an *OR* of 1.84 [(95% *CI*: 1.11–3.03), *p* = 0.018].

#### High convexity tightness

Narita et al. [41] and Virhammar et al. [60] measured high convexity tightness and assessed its use in identifying responders. Both used ordinal scales to grade tightness; the former found that it was significantly correlated with change in INPHGS total score and gait score in multilinear regression analysis, (*B* = 0.99, *R*^2^ = 0.24, *p* = 0 0.017) and (*B* = 0.52, *R*^2^ = 0.21, *p* = 0.006), respectively. They also saw it to be significantly correlated with change in MMSE in simple regression analysis, (*B* = 2.56, *R*^2^ = 0.17, *p* = 0.001). Virhammar et al. [60], however, found no significant difference between responders and non-responders.

#### Bulging of the lateral ventricular roof

Two papers [41, 60] noted the presence of bulges in the lateral roof in relation to shunt response. While Narita et al. [41] measured bumps above the thalamus in the transverse plane, Virhammar et al. [60] measured their presence on the roof of the lateral ventricle, in the sagittal plane. Neither study found an association with shunt response.

### Ventricular size

Four papers [2, 5, 46, 60] investigated measures of ventricular size other than EI. Black [5] found no difference in outcome between those with a ventricular span < 55 mm compared with those > 55 m, on pneumoencephalogram, and calculated its sensitivity as 90% and specificity a of 44.4%. Virhammar et al. [60] measured the widest diameter of the 3rd ventricle but reported it insignificant. Agerskov et al. [2] found no difference in both 3rd and 4th ventricle maximum diameter between responders and non-responders. The latter two studies used MRI. Poca et al. [46], however, used a ventricular score, a composite of multiple measures of the ventricles on CT and found it correlated between score and percentage change on the Digit Span Forward attention test (rho = 0.46, *p* = 0.002).

### Flow void

Two papers [2, 60] graded flow on an ordinal scale from 0 to 3 on MRI. Both Agerskov et al. [2] and Virhammar et al. [60] found that it was not useful in determining shunt outcome. The former found no significant difference between responders and non-responders in grade of flow void, while the latter found an insignificant odds ratio.

### CSF flow dynamics

Two studies [45, 49] evaluated CSF flow dynamics. Stecco et al. [52] measured the aqueductal stroke volume using a FFE sequence on phase contrast cine MRI and found a significant difference (*p* > 0.1) in stroke volume between patients who responded to shunting (271.85 ± 143.032) and those who did not (79.83 ± 31.24). This was corroborated by Poca et al. [45] who measured CSF flow velocity in the sagittal plane through the aqueduct using phase-contrast velocity MRI with retrospective cardiac gating. They found that patients with hyperdynamic CSF flow were more likely to respond, with a sensitivity of 90% and specificity of 50%.

### Cerebral atrophy

McGirt et al. [34] and Hong et al. [20] observed the effect of cerebral atrophy on shunt outcome. Both found no relation with SR. McGirt et al. [34] measured atrophy on CT or MRI and reported atrophy in 28% of patients but calculated an insignificant risk ratio. Hong et al. [20] scaled hippocampal atrophy from 0 to 4 according to the Scheltens’ scale on MRI and found no significant difference in atrophy between responders (mean grade 1.8 ± 0.9) and non-responders (1.9 ± 0.6). Univariate analysis showed an insignificant *OR*.

### Deep white matter hyperintensities

Four studies assessed [2, 20, 41, 60] deep white matter hyperintensities and all demonstrated no significant association with shunt outcome, in each grade assessed. All graded deep white matter hyperintensities in the transverse plane, on an ordinal scale from 0 to 3, both Narita et al. [41] and Hong et al. [20] used Fazekas’s scale [12], while Agerskov et al. [2] and Virhammar et al. [60] used their own. While Narita et al. [41] used T1-weighted MRI, the rest utilized T2-FLAIR MRI.

### Lacunae

Hong et al. [20] and Murakami et al. [39] reported a significant association between the presence of lacunae and failure to respond to shunting. The former counted lacunae manually on T2-FLAIR images while the latter used SPECT. Hong [20] found a significant difference between responders and non-responders in the number of lacunae (shunt responders mean lacunae 0.1 ± 0.2 and shunt non-responders mean: 1.1 ± 1.4, p = 0.009). They also calculated a significant *OR* = 0.161 in a univariate analysis. Murakami et al. [39] noted presence of lacunae could predict shunt non-responders with a sensitivity of 71.4% and a specificity of 80%.

### Miscellaneous markers

McGirt et al. [34] investigated corpus callosum distention using CT and MRI and found those with distention (28% of participants) were more likely to respond to shunting, with an overall risk ratio of 1.64 (95% *CI*: 1.05–2.58). Similarly, Agerskov et al. [2] used T1-MRI to grade the widening of the interhemispheric fissure from 0 to 2 but found no significant association with shunt response.

### DESH

Six papers [2, 14, 14, 20, 47, 60] investigated DESH in relation to SR prediction in iNPH. There were varied definitions of DESH. Agerskov et al. [2], Hong et al. [20], and Virhammar et al. [60] defined it as narrow sulci at the high convexity and dilated sylvian fissures. Garcia-Armengol et al. [14] used enlargement of the inferior sub-arachnoid spaces and high convexity subarachnoid spaces. Shinoda et al. [51] combined ventriculomegaly, dilated Sylvian fissures, acute callosal angle, and focal sulcal dilation to form a DESH score. Grahnke et al. [16] did not define DESH and were the only study to employ both CT and MRI; the remaining only used MRI (a combination of T2-FLAIR and T1). Four papers [14, 20, 47, 60] reported DESH’s statistical significance in predicting shunt response. Virhammar et al. [60] and Hong et al. [20] calculated *OR* of: 2.78 [(95% *CI* 1.09–7.061), *p* = 0.032] and 15.167 [(95% *CI* 1.509–152.461), *p* = 0.021], respectively. Garcia-Armengol et al. [14] reported that shunt responders were significantly more likely to have DESH (*p* < 0.001) and calculated DESH’s sensitivity as 79.4% and specificity as 80.8% while Shinoda et al. [51] demonstrated that a higher DESH score predicted improvement in postoperative iNPHGS, MMSE, trail making test-A (TMT-A) and timed 3-m up and go test (TUG-t). They also reported shunt responders had higher DESH scores (6.50 ± 2.0 vs 3.94 ± 1.5 in non-responders; *p* < 0.001). Both Agerskov et al. [2] and Grahnke et al. [16] found no association between DESH and shunt response.

### CT cisternography

Three studies [5, 26, 27] evaluated the use of CTC. Kawaguchi et al. [26] and Kazui et al. [27] used a CTC score (0–3) to compare contrast (iohexol (Omnipaque®: 180 mg/ml)) movement in ventricles to surrounding parenchyma at 0, 6, 24, and 48 h after injection. Black [5] graded images at 72 h post contrast injection into having delayed isotope clearance and failure of convexity ascent, mixed pattern or normal. Kazui et al. [27] found that significant changes in the parietal sulci at 48 h only were predictive of urinary symptom disappearance, *OR*: 0.47 [95% *CI* 0.25–0.88), *p* = 0.02]. Kawaguchi et al. [26] found CTC to have a high sensitivity and PPV with a low specificity of 95%, 85.1%, and 0%, respectively, when decision to shunt was based on stasis in any of lateral ventricles after 24 or 48 h, or parietal cortical sulci or Sylvian fissure after 48 h. In order to achieve the highest specificity, the sensitivity and PPV were somewhat compromised; this occurred when only accepting stasis in the lateral ventricles at 24 h which had a sensitivity of 51.7%, specificity 40%, PPV 83.8%, and NPV 12.1%. Black [5] found no significant difference between CTC patterns and shunt response.

### Cerebral blood flow

Seven papers [3, 8, 23, 27, 39, 62, 64] analyzed the use of cerebral blood flow. Five studies [8, 23, 27, 39, 62] used single positron emission CT (SPECT) and the 3DSRT method, while Zieglelitz et al. [64] and Agerskov et al. [3] used MRI FLAIR perfusion studies. Yamada’s group [62] investigated the percentage increase in regional CBF after technetium-99 m-L, L-ethylcysteinate dimer injection and while they found there was no correlation between recovery of cognitive functions and regional increase in %CBF, they did find that a < 20% increase in CBF post acetazolamide injection could predict improvement in MMSE with a sensitivity of 100% and specificity of 60%. Similarly, Ishii et al. [23] compared resting CBF to post 123-I-iodoamphet-amine injection CBF and stratified patients depending on the anatomical location of CBF reduction: anterior-dominant CBF reduction type (A-type), posterior-dominant CBF reduction type (P-type), and mixed or diffuse CBF reduction type (M-type). They found PPVs for A-type: 0.83, P-type: 0.9, and M-type 0.84, indicating P-type was the most accurate predictor of SR, although all had high PPVs. In a very similar analysis, Kazui et al. [27] observed only M-type could significantly predict improvement post-shunt, and only for the disappearance of urinary symptoms, *OR* [0.26 (95%*CI*: 0.07–0.89) *p* = 0.03]. Using N-isopropyl-p-[123I] iodoamphetamine injection, Murakami et al. [39] found responders have reduced CBF in the frontal base and the anterior part of limbic areas (cingulate gyrus) but did not report *p*-values. Chen et al. [8] used both inhaled xenon and acetazolamide to measure regional CBF, global CBF post ACT challenge, and cerebrovascular activity (CRC). They found a significant difference between non-responders (14.2 mL per 100 g/min) vs responders (24.1 mL per 100 g/min) (*p* = 0.008) in cerebral blood flow post ACT challenge, but no difference in regional CBF was found. They also found a > 20% CRC at the anterior area of the centrum semiovale was significantly different between non-responders (1.06%) and responders (1.41%), but cerebrovascular activity could not be used alone to predict response. Ziegelitz et al. [64] mapped CBF on dynamic susceptibility contrast (DSC) MRI FLAIR images in 15 anatomical locations of the brain. They found a significant negative correlation between regional cerebral blood flow along the white matter profile and grey matter with an improvement in NPH score. Importantly shunt responders had higher rCBF in the basal medial frontal cortex (*p* = 0.019), ROC analysis of the same variable revealed an AUC < 0.854 and using a cutoff of > 0.798 had a sensitivity of 80% and specificity of 100%. Agerskov et al. [3] again used DSC to map cerebral blood flow onto MRI FLAIR images. They found no significant difference in relative cerebral blood flow in any anatomical location between shunt responders and non-responder, neither in cerebral blood volume nor mean transit time.

### Intracranial volume

Palm et al. [42] found no significant difference between responders and non-responders when using MRI to calculate intracranial volume, total brain volume, ventricular CSF volume, and extra-ventricular CSF volume. Yamamoto et al. [63] employed voxel-based morphology on T1-MRI to measure CSF areas: volume of ventricles and Sylvian fissures (vVS) and volume of subarachnoid space at the high/midline convexity (vHCM). vVS/HCM were significantly correlated with change in frontal assessment battery (FAB) (*r* =  − 0.51), trail making test part A (TMT-A) (*r* = 0.59), timed up and go (TUG) time (*r* = 0.63) and TUG steps (*r* = 0.49). vHCM was significantly correlated with change in FAB (*r* = 0.51) and TMT-A (*r* =  − 0.64) and vVS was significantly correlated with change in FAB (*r* =  − 0.56), TUG time (*r* = 0.71) and TUG steps (*r* = 0.55).

### Novel imaging techniques

Aoki et al. [4] used exact-low-resolution-brain-electromagnetic-tomography, a 19 electrode EEG system assessing 5 frequency bands in high convexity areas; this system had a PPV of 61.1% and a NPV of 75% for use identifying shunt responders. Jurcoane et al. [25] employed diffuse tensor imaging using an echo-planar sequence and found that a decrease of > 1% in axial diffusivity could differentiate between responders and non-responders with a sensitivity of 87.5% and specificity of 75%. Similarly, Agerskov et al. [3] reported apparent diffusion coefficients (ADC) in 6 regions of the brain using transverse DWI MRI FLAIR but found no significant difference between responders and non-responders in ADC in any region. Chen et al. [8] demonstrated the use of magnetic resonance spectroscopy using the default chemical shifting method of TI/T2-FLAIR images. They measured the N-acetyl aspartate/creatine ratio (NAA/Cre) change at the anterior, middle, and posterior centrum semiovale but found no correlation between clinical response and NAA/Cre ratio. Wu et al. [61] and Kuchcinski et al. [31] both used automated methods of image analysis to predict shunt response. The former segmented a T1-MRI brain image into 283 region of interest (ROI) and then employed a machine deep learning algorithm, trained on those improved post CSF tap test, to predict those who will respond to shunting. The algorithm also used other variables such as age, gender and pre-op Tinetti scores as inputs. Its predicted Tinetti and MMSE scores post shunting significantly correlated with the ground truth with *r* = 0.8 and *r* = 0.88 respectively at best performance, indicating it is a strong predictive algorithm. Kuchcinski et al. [31] used T1-MRI to automate measurements of sulcal morphology — the size of the 10 sulcal openings — as well as the ratio of different sulci. They found that the lateral and superior temporal sulci were significantly correlated with score improvement: *r* = 0.42, *p* = 0.02; *r* = 0.38, *p* = 0.03 respectively.

### Meta-analysis

**Fig. 8:**
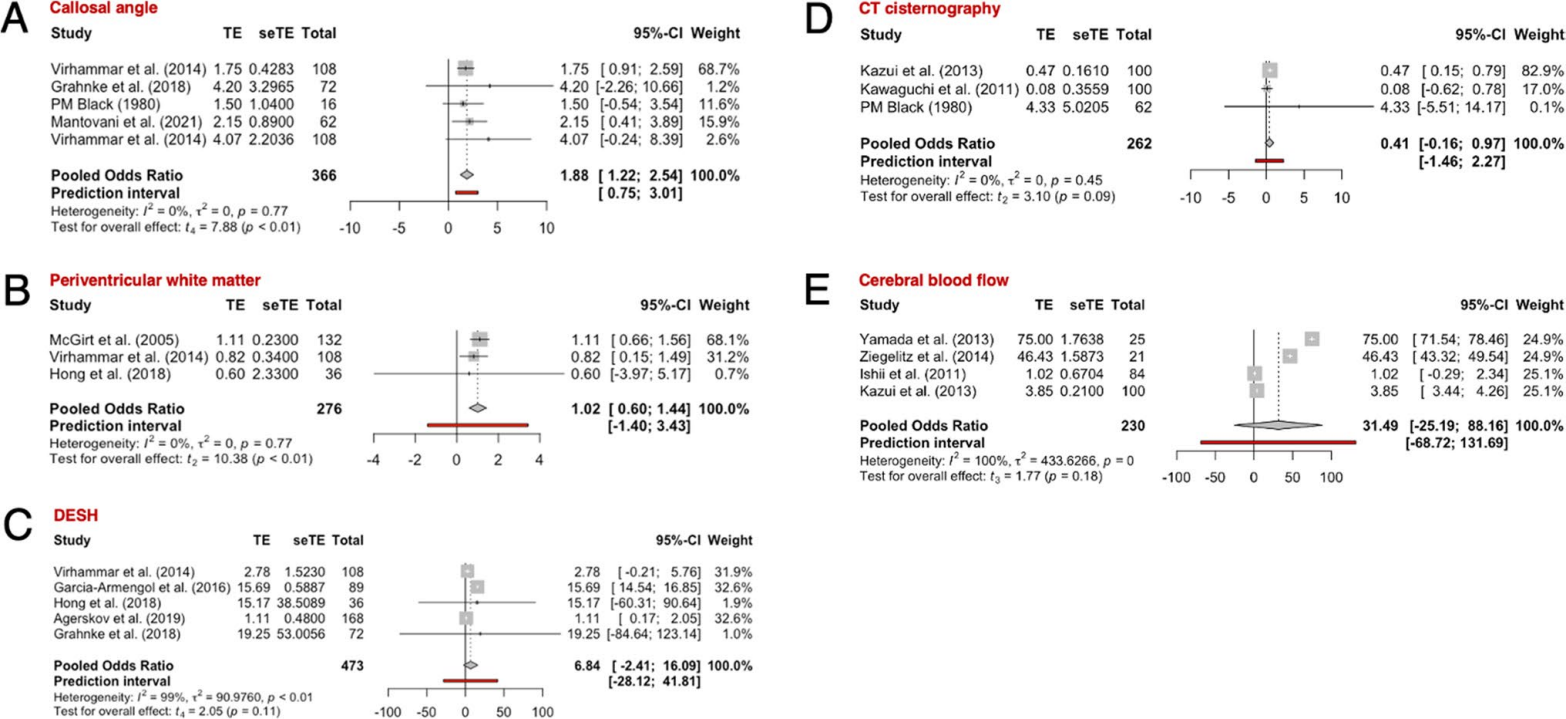
**A–E** Forest plots indicating and visualizing the treatment effect (“TE”) size in diagnostic odds ratio in the context of predicting shunt response in iNPH patients are shown for each of the following radiological markers, in this order: “Callosal angle”, “Periventricular white matter”, “DESH”, “CT cisternography” and “Cerebral blood flow” (*n* = 14 studies) [2, 5, 14, 16, 20, 23, 26, 27, 33, 34, 59, 60, 62, 64]. The size of the grey square of the “Diagnostic Odds Ratio” visual correlates to study sample size and the straight line indicated the confidence interval. The diamond at the bottom indicates the overall pooled odds ratio. The red bar below it indicates the prediction interval. Heterogeneity is indicated by the chi-squared statistic (*I* ^2^) with associated *r*^2^ and *p*-value. The 95% confidence intervals (*CI*) are shown in squared bracket ([]). *p*-value < 0.05 is deemed significant. Furthermore, for every study the following are displayed: study author with publication date (“Study”), total sample size number for each study (“Total”), and standard error of the treatment effect (“seTE”), test for significance of overall effect size as t_4_ and *p*-value, and weighting of each study in percentage (%). Significant pooled odds ratios were yielded for “Callosal angle” (**A**) and “Periventricular white matter” (**B**) (both *p* < 0.01), however “DESH” (**C**), “CT cisternography” (**D**) and “Cerebral blood flow” (**E**) yielded insignificant pooled odds ratios (*p* > 0.05). DESH, disproportionately enlarged subarachnoid space hydrocephalus

The meta-analysis was conducted for the following radiological markers, which met the inclusion criteria for meta-analysis: callosal angle, periventricular white matter changes, DESH, CT cisternography, and cerebral blood flow (Fig. 8). For callosal angle five studies [5, 16, 33, 59, 60], two scoring low risk of bias and three scoring moderate risk were included with a pooled sample size of *n* = 361 shunted patients, and the pooled *OR* estimate between shunt responders and shunt non-responder patients was 1.88 *OR* (*CI* 95%: 1.22–2.54), with *t* = 7.88 (*p* < 0.01) (Fig. 8). For periventricular white matter changes three studies [20, 34, 60], two scoring moderate and one study scoring serious risk of bias, with a pooled sample size of *n* = 271 shunted patients, were included and the pooled *OR* estimate was 1.01 *OR* (*CI* 95%: 0.59–1.44), with *t* = 10.27 (*p* < 0.01) (Fig. 8). For DESH five studies [2, 14, 16, 20, 60], three scoring moderate and two scoring low risk of bias, with a pooled sample size of *n* = 468 shunted patients, were included and the pooled *OR* estimate was 6.85 *OR* (*CI* 95%: − 2.40–16.09), with *t* = 2.06 (*p* = 0.11) (Fig. 8). For CT cisternography, three studies [5, 26, 27], two scoring low and two scoring moderate risk of bias, with a pooled sample size of *n* = 262 shunted patients, were included and the pooled *OR* estimate was 0.41 *OR* (*CI* 95%: − 0.16–0.97), with *t* = 3.10 (*p* = 0.09) (Fig. 8). For cerebral blood, flow four studies [23, 27, 62, 64], two scoring low and two scoring moderate risk of bias, with a pooled sample size of *n* = 201 shunted patients, were included and the pooled *OR* estimate was 31.49 *OR* (*CI* 95%: − 25.19–88.16), with *t* = 1.77 (*p* = 0.18) (Fig. 8). Statistical heterogeneity was found to be significant (*p* < 0.05) for DESH and CBF. Overall, the meta-analyses indicated significant odd ratios only for callosal angle and periventricular white matter changes: shunt responders are 1.88 times more likely than shunt non-responders to have a smaller angle on radiological imaging, as well as being 1.02 more likely to have abnormal periventricular white matter changes. All other examined radiological markers were found to not significantly differentiate between shunt responders and shunt non-responders.

#### Sensitivity analysis and linear regression

The meta-regressions scored the influence of all covariates on the overall effect size of each radiological marker (*OR*) (Table 22). For DESH, there were two significant co-variants that were found: “females” and “imaging plane” (particularly the coronal plane, Fig. 4) (*p* = 0.0458 and *p* = 0.0087, respectively). The results of the meta-regression in Fig. 4 imply that the proportion of females included in the study or the imaging plane chosen by each study may positively skew the odds ratio of DESH as radiological marker of shunt-responsive iNPH. To further assess the impact of the covariate “females,” another subgroup meta-analysis for DESH was performed, omitting the study with the highest proportion of females, namely Grahnke et al. [16]. However, this did not have a strong effect, as the SMD remained insignificant at *p* = 0.17 (Supplementary Material: Fig. 3). However, the statistical heterogeneity remained significant (*p* < 0.05). To address the heterogeneity for DESH, multiple sub-group meta-analyses were run, and the significant skewer of the data was found to be Agerskov et al., which is the study with the methodology most different from the rest of the included studies: the study employs non-NPH standard cognitive tests (the identical forms and Bingley memory test) while the other studies use validated NPH cognitive scales such as the INPH grading scale; and their definition of DESH was categorically different to the other studies. Eliminating Agerskov et al. from the meta-analysis led to the heterogeneity being eliminated (*I*^2^ = 0, *p* = 0.69) while the odds ratio of DESH remained insignificant (*p* > 0.05) (Supplementary Material: Fig. 4). A sensitivity analysis was not performed for “imaging plane” as the majority of studies used the coronal plane. The significantly negatively skewing co-variate were “females (*p* = 0.036) for cerebral blood flow (Table 22). To assess the statistical effect of the proportion of females on the *OR* of CBF to predict shunt response in iNPH, the study with the highest proportion of females, namely Kazui et al. [27] was omitted. However, the result remained insignificant at *p* = 0.20 (Supplementary Material: Fig. 5). Hence, overall, the co-variates highlighted in bold in Table 22 may skew the data but do not have a significant effect on the odds ratio of each radiological marker. PVM had a significant co-variate with negative estimate, namely “Year” (*p* = 0.0336), however, this could not be assessed by means of a sensitivity analysis due insufficient number of studies for PVM before 2010, and marginal spread of the remaining studies would have required more studies for a robust sensitivity analysis. A subgroup meta-analysis that excludes McGirt et al. [34], the only study scoring serious overall risk of bias, could not be performed to insufficient number of studies.

**Table 22:** Mixed-effects single-variate meta-regression

	CA	PVM	DESH	CTC	CBF
~ **Covariates**	Regression coefficients				
~ *Sample*	0.0011 (0.0087)	0.0096 (0.0962)	** − **0.1449 (0.0697)	-	** − **1.0236 (0.3098)
~ *Year*	0.0141 (0.0222)	− 0.0326 * [*p* = 0.0336] (0.0017)	** − **0.5473 (1.8678)	-	15.9875 (16.5183)
~ *Age*	0.4543 (0.8774)	** − **0.1660 (0.0036)	0.1975 (0.6287)	-	** − **13.1809 (11.2385)
~ *Females*	4.8480 (6.3222)	5.0498 (1.0436)	101.094 * [*p* = 0.0458] (30.6645)	-	− 420.111 * [*p* = 0.0306] (75.2840)
~ *HTN*	9.0327 (42.3043)	** − **1.2670 (0.0970)	13.8376 (4.3349)	-	-
~ *Gait-*	** − **86.7841 (90.9726)	0.1696 (4.0455)	** − **30.8103 (27.0620)	-	-
~ *mRS*	-	-	6.5049 (44.5366)	-	-
~ *MMSE*	-	-	-	-	** − **3.7608 (3.3687)
~ *EI*	-	-	** − **56.2127 (**− **16.1414)	-	1.9071 (0.5753)
~ *CA*	N/A	-	** − **0.2079 (0.1071)	-	-
~ *Depression*	2.0030 (9.3808)	-	-	-	-
~ *S-R*	** − **3.4882 (8.1168)	0.0210 (8.0901)	3.0879 (0.9748)	-	139.9761 (245.2653)
~ *Compl*	-	3.1574 (0.2010)	** − **36.4027 (0.4205)	-	-
~ *imaging plane*	-	-	Coronal: 15.6923 * [*p* = 0.0087] (0.2147)	NA	-
~ *imaging modality*	1.5 T MRI: 1.9071 (0.5753) 3.0 T MRI: 2.1500 (0.9813) CT or MRI: 4.2019 (3.3224)	-	1.5 T MRI: 1.3047 (0.5238) 3.0 T MRI: 15.1670 (38.5106) CT or MRI: 19.2500 (53.0068)	-	-

The results of the meta-regression of the meta-analyses of Callosal angle (“CA), periventricular white matter (“PVM”) changes, disproportionately Enlarged Subarachnoid space Hydrocephalus (“DESH”) and cerebral blood flow (“CBF”), for each of the covariates (sample, year, age, females, HTN, Gait-, mRS, EI, CA, depression, S-R, Compl.) as independent variable to the dependent variable odds ratio. In round brackets is the standard error. If significance is yielded (denoted with * and bold regression coefficient), the *p*-value of the regression coefficient is shown in squared bracket only if significant, otherwise assume non-significance. A meta-regression was not performed for CT cisternography (“CTC”) due to small sample size (*n* = 3). Significance is assumed for *p* < 0.05. If a covariate was covered by < 3 studies for a respective radiological marker, then a regression analysis was omitted (“- “) for this respective relationship due to insufficient data for strong regression analysis. The different explanatory variables were calculated singularly as sole covariates in separate meta-regressions

## Discussion

The major observation of this systematic review and meta-analysis is that only the radiological marker callosal angle and periventricular white matter change significantly differentiated iNPH shunt responders from shunt non-responders. However, both markers were weak predictors on their own. The other four radiological predictors (Evan’s index, DESH, cerebral blood flow, CT cisternography) did not significantly differentiate shunt responders from non-responders. This finding is of definite significance given that the radiological markers Evan’s index and DESH are included in current Japanese diagnostic guidelines [40] and the EI in the current American-European iNPH guidelines [40].

Radiological markers of ventricular size remain a pivotal part of the diagnostic guidelines of iNPH, together with the clinical presentation and supplementary tests [40]. It may, however, seem a paradox that fulfillment of diagnostic criteria does not necessarily imply clinical response to the only existing treatment — shunt surgery. Therefore, the diagnostic guidelines have also differentiated between shunt responsive and shunt non-responsive iNPH [48]. Accordingly, definite iNPH, according to the Japanese guidelines, is characterized by clinical response to shunting [40]. Since the iNPH disease was described in 1965, numerous methods and tests have been used to best predict clinical response to shunting in these patients. Over the years, the shunt response rate seems to have improved [15, 56], though several reports point to a low shunt response rate, even below 50% [5, 16]. The prediction of shunt response remains a challenge to physicians treating iNPH patients. In a previous systematic review and meta-analysis, the most accurate predictors of shunt response were ICP monitoring of pulse pressure (mean ICP wave amplitude, MWA) [10], followed by extended lumbar drain, and thereafter infusion testing [54]. The invasive tests are, however, more costly and carry a higher risk profile [11]. Therefore, a search for less invasive predictors of shunt response in iNPH is highly warranted [11]. In this regard, we recently reported in another systematic review and meta-analysis that the biochemical markers such as lumbar CSF levels of Phosphorylated-Tau and Total-Tau were significantly increased in iNPH shunt non responders compared to shunt responders [55]. Similarly, non-invasive or less invasive radiological markers predicting shunt response would be preferable.

A steep callosal angle (< 90°) is a widely used routine marker and indicates hydrocephalus due to the obstructed expansion of the corpus callosum at the falx and the continued rise of the roof of the lateral ventricles [32]. Interestingly, Cagnin et al. [7] showed that it was possible to reliably differentiate between patients with either Alzheimer’s, dementia with Lewy bodies, or iNPH using a callosal angle cutoff of 123° cutoff, at a sensitivity of 95.2% and specificity of 100%. The similarity in the symptomology of the dementia subtypes is thought to be one of the greatest causes of shunt failure in iNPH [35], and callosal angle estimation may provide an opportunity to overcome this. Also, interestingly, the correlation analysis found the callosal angle to be positively correlated with the rate of complications following shunt insertion [35], possibly indicating that the higher callosal angle (i.e., the less severe the disease), the higher the complication risk is. As iNPH shunt responders generally have a lower callosal angle than non-responders, the higher risk of complications coupled with the lower chance of shunt response outlines the importance of not proceeding with shunting in patients with large callosal angles. This meta-analysis found callosal angle to be the most reliable and, relatively speaking, strongest predictor of shunt response in iNPH. Therefore, we advocate that it should be given clear priority over other radiological markers. On the other hand, it should be noted that a DOR = 1.83 is much less predictive of shunt response than supplementary tests such as intracranial pressure monitoring and extended lumbar drainage, which have a DOR of 50.9 and 27.7 [54], respectively. In clinical practice, the callosal angle is usually calculated at the level of the posterior commissure. Recently, Mantovani et al. [33] have proposed to additionally measure the anterior callosal angle (measured on the anterior commissure plane), which they hypothesized may be more reflective of the forebrain dysfunction in iNPH. Combining both the posterior and anterior callosal angle, the diagnostic effectiveness of this radiological may be enhanced.

All included studies which individually investigated the relationship of periventricular white matter changes and shunt response in iNPH found no difference between responders and non-responders [2, 5, 20, 34, 41, 46]. Our meta-analysis however did find it to be one of the two significant radiological predictors of shunt response. We believe the underreporting of its significance to be a sample size error, which was mitigated using a meta-analysis, providing an amplified sample size. Sarica et al. [50] investigated whether increased CSF pressure causes alteration of periventricular white matter microstructure in patients with idiopathic intracranial hypertension (IIH) and lead to these changes, which are often reversible upon treatment. Although in NPH the ICP tends to be normal or slightly above normal in general, it has been shown by Eide et al. [10] that shunt-responsive iNPH patients had significantly higher mean ICP than shunt-non-responsive iNPH patients. Due to the relative increase in mean ICP wave amplitude, it may be that these patients also have periventricular white matter changes, similar to IIH patients. However, the sample size of the meta-analysis was low (*n* = 3), and the diagnostic *OR* is almost 1; hence, the validity of this finding is limited. We believe that periventricular white matter changes may be a promising radiological predictor, but more research is needed to consolidate its usefulness.

DESH remains a diagnostic criterion in the updated Japanese iNPH guidelines [40] and has previously been shown to be predict shunt response in several studies [14, 24]. However, our findings indicate it is not a significant indicator of iNPH shunt response. Agerskov et al. [2] suggests that selection bias in other papers may lead to overreporting of DESH’s usefulness as participants in some studies required an element of DESH positivity in order to be selected for shunting. On the contrary, none of the papers included in our meta-analysis had an element of DESH in their selection criteria. Agerskov et al. had the joint highest weighting of 31.6% in our meta-analysis, and its findings were contrary to most other papers analyzing DESH. There are two possible reasons for this discrepancy, one is the study’s use of non-NPH standard cognitive tests (the identical forms and Bingley memory test) while most others used validated NPH cognitive scales such as the INPH grading scale. We are unsure as to the validity of the former two tests. Secondly, their definition of DESH only included dilation of the Sylvian fissure with obliteration of the high convexity sulci, whereas most other studies [40, 47] include ventriculomegaly and focal sulcal dilatation as well. Given the findings of our meta-analysis, we question the position of DESH as a main radiological criterion of shunt responsive iNPH. Given that the findings of this meta-analysis are diametrical to the Japanese guidelines, as well as those of our recent meta-analyses on clinical tests and biochemical markers of shunt response [54, 55], the question arises whether the Japanese iNPH guidelines are a reliable source for clinicians on the topic of iNPH management. The authors believe that the narrative review design of the Japanese iNPH guidelines may undermine the significance of their recommendations, in addition to much of their diagnostic review being graded as weak evidence. However, the Japanese iNPH guidelines still recommended the use of DESH, as well as the use of CA to predict shunt response, despite the lack of a meta-analysis, contrary to our meta-analysis findings. The authors believe that future guidelines on iNPH management must strive to run meta-analyses and systematic reviews before making definitive recommendations.

A landmark paper by Kockum et al. [28] reports the use of the Radscale in evaluating iNPH patients; it consists of a scale comprising of Evan’s index, callosal angle, size of temporal horns, narrow high-convexity sulci, dilated Sylvian fissures, focally dilated sulci, periventricular white matter changes, and bulging of the lateral ventricular roof. We have assessed each feature and found that only the callosal angle and periventricular white matter changes were significant in identifying shunt response. This study initially correlated the Radscale with symptom severity in iNPH patients who had ≥ 2 symptoms of Hakim’s triad but underwent no further confirmatory investigations. It is generally considered that symptomatology alone is insufficient to diagnose iNPH due to the similarity of symptom mimics, a concept recently supported by a systematic review and meta-analysis [54]. Furthermore, many of these features are in fact also found in symptom mimics, for example, periventricular white matter changes have been noted in Alzheimer’s disease [65]. Kockum et al. [29] later published a study highlighting its usefulness in identifying confirmed iNPH shunt responders versus healthy controls; this study was better designed and showed again the Radscale’s utility; however, we recommend a superior design comparing the Radscale scores between shunt responders and shunt non-responders. Interestingly, our study found age and Evan’s index to be negatively correlated, despite literature reporting these two factors to be positively correlated [6]. A reason for this may be that the included studies in our analysis had a shared source of bias leading to a skewed patient sample, where older patients had a smaller Evans index. However, another explanation may be that the findings regarding Evan’s index have been limited so far in validity due to sample size error, and in fact, it may not only have a different correlation to age than expected; perhaps, its use in the diagnosis of NPH may be limited too. We could not perform a meta-analysis on EI due to limited comparable data; however, future studies must strive to do this, to elucidate the relevance of EI.

In the present study, the choice of imaging modality was not found to be a significant factor influencing the diagnostic efficacy of the radiological markers. It was previously reported by Hurley et al. [22] that MRI may visualize CSF flow better than CT, and as hyperdynamic CSF flow through the ventricle is a marker of shunt responsive NPH, Hurley et al. [22] proposed to measure CSF flow as a marker of iNPH. Due to the limited number of studies, no meta-analysis was conducted on this marker. More studies are needed to establish its use. Until it has been proven as a powerful marker, the choice between CT and MRI is statistically not a relevant one but must be judged based on radiation exposure and availability. Due to limited data, it was not possible to assess the impact of imaging plane for each biomarker. More studies will therefore be needed to confidently recommend which plane setting is most effective for each biomarker.

A strength of this study has been seen that it highlights important novel techniques. In particular, machine learning has been shown by Wu et al. [61] to provide an accurate prediction of not only shunt response, but also the degree of improvement. Additionally, Rudhra et al. [49] achieved 98% sensitivity and 100% specificity in identifying iNPH patients using MRI against healthy controls, although their study did not aim to identify shunt responsive iNPH patients; therefore, it could be argued that this was a clinically insignificant task. The obvious advantage of machine learning is its ability to incorporate features outside of radiology into its predictive model, such as patient demographics, co-morbidities, and symptom severity. An algorithm that combines these features with invasive clinical tests such as ICP measurements or ELD could be a very powerful tool for clinicians and for which further research is required. Machine learning has already proven successful in identifying spinal cord compression in patients with degenerative cervical myelopathy, and future research is advocated to harness similar techniques in the accurate diagnosis of iNPH [36]. The authors believe that only by combining the multitude of readily available MRI and CT radiological markers in the context of a machine learning–based prediction model, the diagnostic potential of radiological markers may be greatly improved, to perhaps an extent that the non-invasive radiological SR predictors may match the current gold standard, invasive SR predictors.

## Limitations

The main limitation of our meta-analysis is the heterogeneous methodology of measuring specific radiological parameters employed by the included studies. Albeit referring to the same parameter nominally, often the anatomical location, imaging plane, or modality differed and thereby hindered direct statistical comparison. However, the authors aimed to control this heterogeneity by assessing the impact of co-variates by means of regression, sub-group meta-analyses, and sensitivity analyses. Given that, upon employing these measures, the *I*^2^ heterogeneity for all markers except PVM turned insignificant; we believe our findings for these to be robust. Nonetheless, future meta-analyses to consolidate our findings will be beneficial, particularly for PVM. Future studies exploring the use of radiological markers of shunt-responsive iNPH must aim to employ the conventional methodologies to assess the radiological marker’s prediction of shunt response, to allow for a valid comparison by means of a meta-analysis. Furthermore, 8 of the studies included did not account for neurological co-morbidities in the statistical analysis of their results [5, 20, 34, 39, 46, 60, 62, 64], which are an important source of potential bias and must be controlled by regression analysis. Statistical heterogeneity was only detected for DESH and PVM. For these two markers, our findings hence, further research employing a stratified approach in patient selection and subsequent analysis is needed to consolidate our findings and allow for a robust regression analysis on the impact of neurological co-morbidities on the diagnostic efficiency results of the included studies.

## Conclusion

Callosal angle (DOR = 1.88, *p* < 0.01) and periventricular white matter changes (DOR = 1.01, *p* < 0.01) are statistically the only diagnostically effective radiological predictors of shunt response in iNPH patients; all other radiological markers do not significantly differentiate between shunt responders and non-responders. However, due to the DORs of callosal angle and periventricular white matter changes approximating 1, they are insufficient as sole predictors and are advised to be used only in combination with other clinical tests and biochemical markers of shunt response. Future research must evaluate the combined use of multiple radiological predictors such as the RADSCALE, particularly using state-of-the-art predictive modelling techniques such as machine learning, as doing so may yield beneficial additive effects that may allow for more robust radiological prediction of shunt response in iNPH.

## Supplementary Information

Below is the link to the electronic supplementary material.

Supplementary file1 (PDF 1304 KB)

## Abbreviations

CA: Callosal angle
CSF: Cerebrospinal fluid
CT: Computerized tomography
CTC: Computerized tomography cisternography
DWMH: Deep white matter hyperintensities
DTI: Diffuse tensor imaging
DESH: Disproportionately enlarged sub-arachnoid space hydrocephalus
DSC: Dynamic susceptibility contrast
EI: Evan’s index
FAB: Frontal assessment battery
IIH: Idiopathic intracranial hypertension
iNPHGS: Idiopathic normal pressure hydrocephalus grading scale
iNPH: Idiopathic NPH
LOESS: Locally estimated scatterplot smoothing
ML: Machine learning
MRI: Magnetic resonance imaging
MMSE: Mini-mental state exam
mRs: Modified Rankin scale
NPH: Normal pressure hydrocephalus
OR: Odds ratio
OCEBM: Oxford Centre of Evidence-Based Medicine
PVWM: Periventricular white matter
PEG: Pneumoencephalogram
PRISMA: Preferred Reporting Items for Systematic Reviews and Meta-Analyses
ROI: Region of interest
rCBF: Relative cerebral blood flow
S-NR: Shunt non-responders
S-R: Shunt responders
SR: Shunt response
SPECT: Single positron emission CT
SPLOM: Scatter plot of matrices
TUG-t: Timed 3-m up and go test
TMT-A: Trail making test-A
vHCM: Volume of subarachnoid space at the high/midline convexity
vVS: Volume of ventricles and Sylvian fissures
WMS: Wechsler memory score
