# Supplementary material for: Radiological predictors of shunt response in the diagnosis and treatment of idiopathic normal pressure hydrocephalus: a systematic review and meta-analysis
Source: Acta Neurochir (Wien). 2022 Nov 26;165(2):369–419. doi: 10.1007/s00701-022-05402-8 (PMC9922237; doi:10.1007/s00701-022-05402-8)
Supplement: Supplementary file 1 — Supplementary file1 (PDF 1304 KB) [file 701_2022_5402_MOESM1_ESM.pdf]

Supplementary Material

Radiological predictors of shunt response in the diagnosis and treatment of idiopathic normal pressure hydrocephalus: a systematic review and meta-analysis.

Santhosh G. Thavarajasingam[1*] ; Mahmoud El-Khatib[1*] ;

Kalyan Vemulapalli[1] ; Hector A. Sinzinkayo Iradukunda[1] ;

Sajeenth Vishnu K.[1] ; Robin Borchert[2] ;

Salvatore Russo[3] ; Per K. Eide[4,5]

INSTITUTION:

1. Faculty of Medicine, Imperial College London, London, United Kingdom.
2. Department of Clinical Neurosciences, Cambridge University Hospital NHS Trust, Cambridge, United Kingdom
3. Department of Neurosurgery, Imperial College Healthcare NHS Trust, London, United Kingdom
4. Department of Neurosurgery, Oslo University Hospital – Rikshospitalet, Oslo, Norway
5. Institute of Clinical Medicine, Faculty of Medicine, University of Oslo, Oslo, Norway

Contents

Supplementary Figure 1 4
Supplementary Figure 2 6
Supplementary Figure 3 7
Supplementary Figure 4 9
Supplementary Table 1 10
Supplementary Table 2 13
Supplementary Table 3 14
Supplementary Table 4 15
Supplementary Table 5 17
Supplementary Table 6 18
References 18

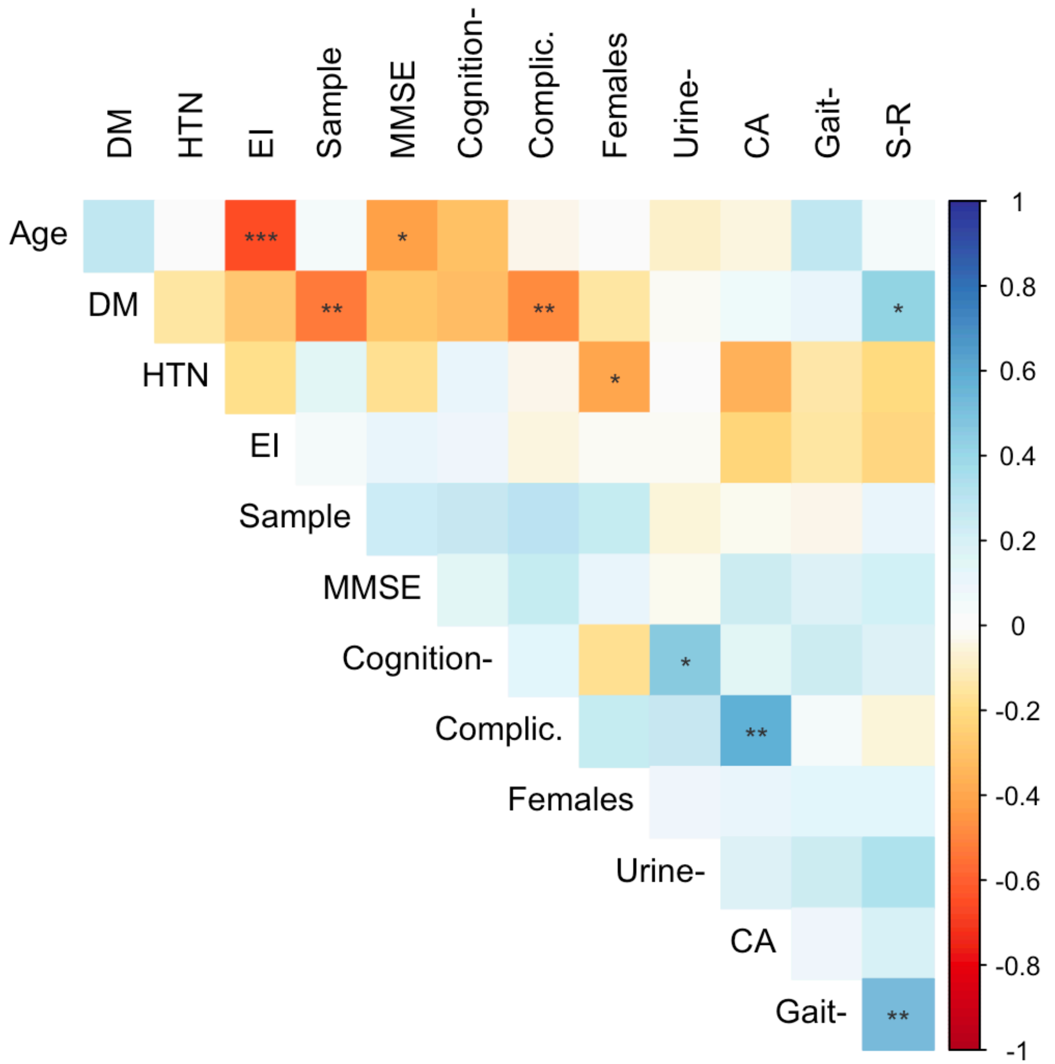
DM
HTN
EI
Sample
MMSE
Cognition-
Complic.
Females
Urine-
CA
Gait-
S-R
Age
***
*
**
**
*
*
*
**
**
1
0.8
0.6
0.4
0.2
0
-0.2
-0.4
-0.6
-0.8
-1

Supplementary Figure 1: A correlation matrix, based on machine learning imputation for missing values, visualises the relationships of following parameters among all studies included in the systematic review (n=28): The following parameters are used here: Patient sample size ("Sample"), mean age of the patients ("Age"), proportion of patient sample being female ("Females"), pre-existing diabetes mellitus ("DM"), pre-existing arterial hypertension ("HTN"), gait deficits ("Gait-", 94%), cognitive deficits ("Cognition-"), urinary dysfunction ("Urine-"), mean proportion of patients being shunt-responsive ("S-R"), and proportion of complications ("Complic."), mean patient scores on the Mini Mental State Exam ("MMSE"). Furthermore, mean patient scores for Evan's Index ("EI") and mean values for Callosal Angle ("CA"). The legend bar at the right of the matrix explains the colouring. One asterisk (*) indicates a statistical significance of p<0.05, two asterisks (**) indicate p<0.01, three asterisks (***) indicate p<0.001.

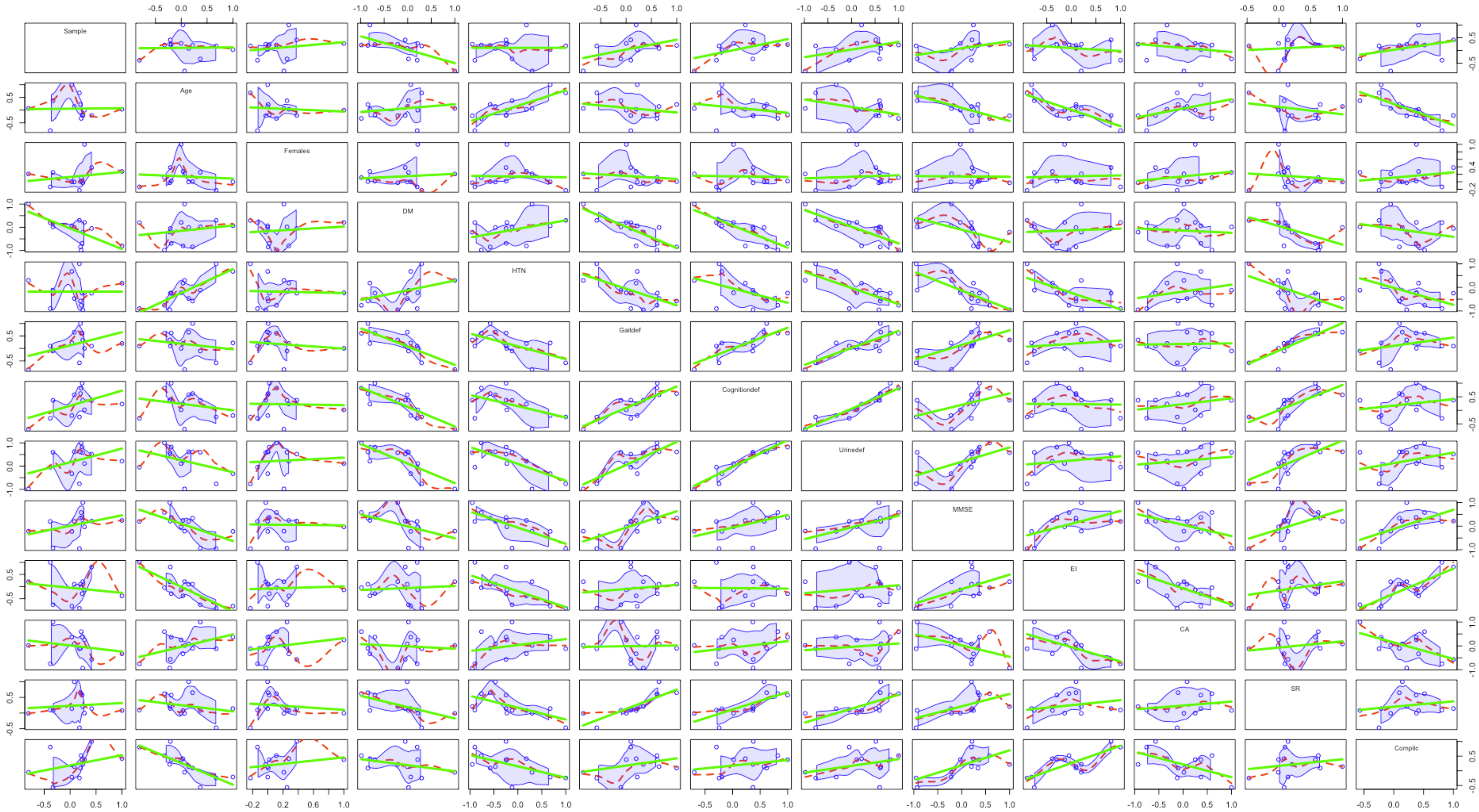
Sample
Age
Females
DM
HTN
Gaitdef
Cognitiondef
Urinedef
MMSE
EI
CA
SR
Complic

Supplementary Figure 2: A scatterplot matrix without machine learning imputation visualises the pairwise relationships of following parameters among all studies included in the systematic review (n=28): The following parameters are used here: Patient sample size ("Sample"), mean age of the patients ("Age"), proportion of patient sample being female ("Females"), pre-existing diabetes mellitus ("DM"), pre-existing arterial hypertension ("HTN"), gait deficits ("Gait-", 94%), cognitive deficits ("Cognition-"), urinary dysfunction ("Urine-"), mean proportion of patients being shunt-responsive ("S-R"), and proportion of complications ("Complic."), mean patient scores on the Mini Mental State Exam ("MMSE"). Furthermore, mean patient scores for Evan's Index ("EI") and mean values for Callosal Angle ("CA"). The green line is the linear regression line, and the red line is the LOESS (locally estimated scatterplot smoothing) regression line, the blue bubbles are the data points, and the blue-lined area is the ellipse.

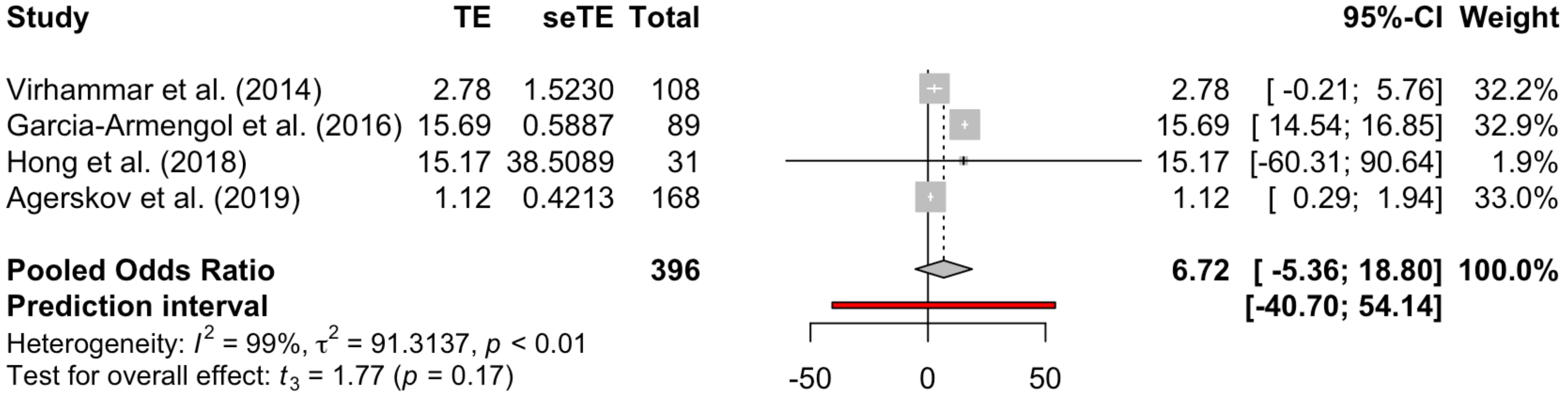

Supplementary Figure 3: Forest plot indicating and visualizing the treatment effect ("TE") size in diagnostic odds ratio in the context of predicting shunt response in iNPH patients are shown for DESH (n = 4 studies), after having excluded the study with the highest proportion of females (Grahnke et al., 2018)[3]. The results remained insignificant with p=0.17 suggesting insignificant effect of the female covariate on the odds ratio of the radiological predictor (DESH) as seen in main paper Figure 6C. The size of the grey square of the "Diagnostic Odds Ratio" visually correlates to study sample size and the straight line indicated the confidence interval. The diamond at the bottom indicates the overall pooled odds ratio. The red bar below it indicates the prediction interval. Heterogeneity is indicated by the chi-squared statistic (I^2) with associated r^2 and p-value. The 95% confidence intervals (CI) are shown in squared bracket ([ ]). P-value < 0.05 is deemed significant. Furthermore, for every study the following are displayed: study author with publication date ("Study"), total sample size number for each study ("Total"), and standard error of the treatment effect ("seTE"), test for significance of overall effect size as t_4 and p-value, and weighting of each study in percentage (%). DESH, Disproportionately Enlarged Subarachnoid space Hydrocephalus

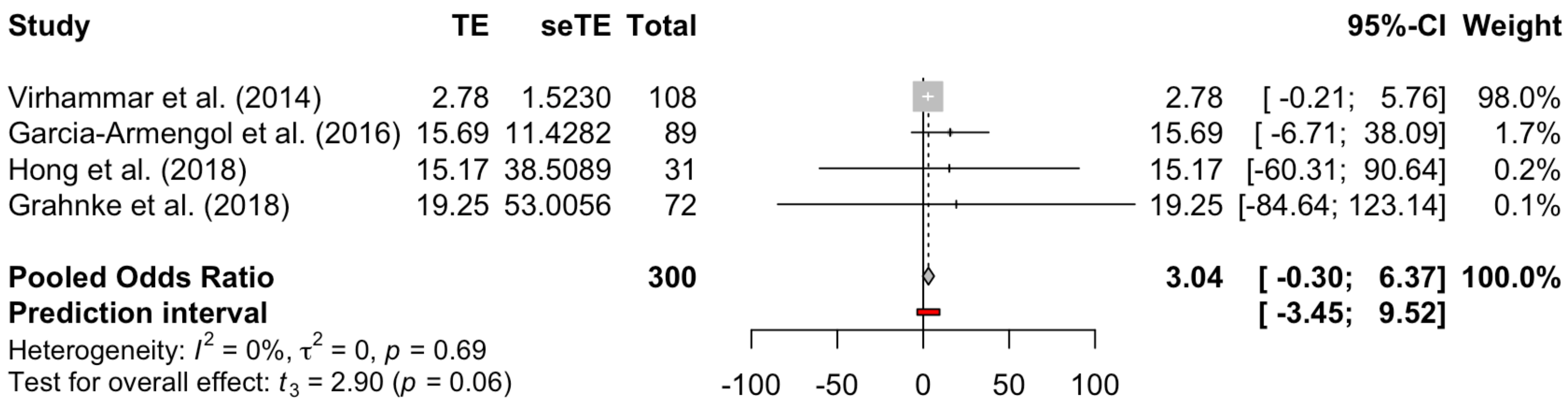

Supplementary Figure 4: Forest plot indicating and visualizing the treatment effect (“TE”) size in diagnostic odds ratio in the context of predicting shunt response in iNPH patients are shown for DESH (n = 4 studies), after having excluded the study with the most different methodology (Agerskov et al., 2019) [3]. The results remained insignificant with p=0.06 and heterogeneity became insignicant (p=0.69). The size of the grey square of the “Diagnostic Odds Ratio” visually correlates to study sample size and the straight line indicated the confidence interval. The diamond at the bottom indicates the overall pooled odds ratio. The red bar below it indicates the prediction interval. Heterogeneity is indicated by the chi-squared statistic (I^2) with associated r^2 and p-value. The 95% confidence intervals (CI) are shown in squared bracket ([ ]). P-value < 0.05 is deemed significant. Furthermore, for every study the following are displayed: study author with publication date (“Study”), total sample size number for each study (“Total”), and standard error of the treatment effect (“seTE”), test for significance of overall effect size as t_4 and p-value and weighting of each study in percentage (%). DESH, Disproportionately Enlarged Subarachnoid Space Hydrocephalus

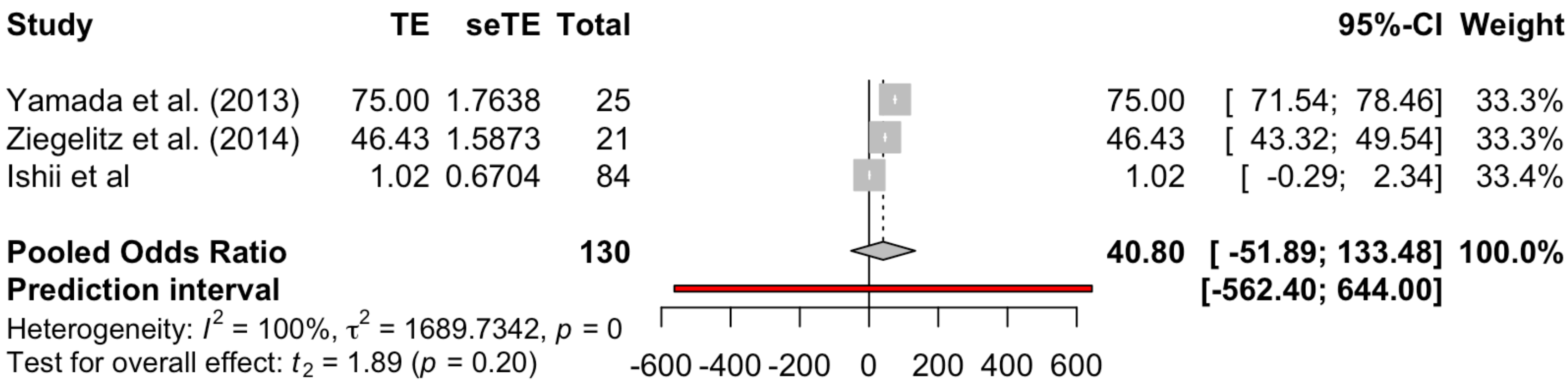

Supplementary Figure 5: Forest plots indicating and visualizing the treatment effect ("TE") size in diagnostic odds ratio in the context of predicting shunt response in iNPH patients are shown for CBF (n = 3 studies), after having excluded the study with the highest proportion of females (Kazui et al., 2013)[6]. The results remained insignificant with p=0.20 suggesting insignificant effect of the female covariate on the odds ratio of the radiological predictor (CBF) as seen in main paper Figure 6E. The size of the grey square of the "Diagnostic Odds Ratio" visually correlates to study sample size and the straight line indicated the confidence interval. The diamond at the bottom indicates the overall pooled odds ratio. The red bar below it indicates the prediction interval. Heterogeneity is indicated by the chi-squared statistic (I^2) with associated r^2 and p-value. The 95% confidence intervals (CI) are shown in squared bracket ([ ]). P-value < 0.05 is deemed significant. Furthermore, for every study the following are displayed: study author with publication date ("Study"), total sample size number for each study ("Total"), and standard error of the treatment effect ("seTE"), test for significance of overall effect size as t_4 and p-value, and weighting of each study in percentage (%). CBF, Cerebral Blood flow.

Supplementary Table 1: The 27-point checklist furnished by the Preferred Reporting Items for Systematic Reviews and Meta-Analyses (PRISMA) 2020 statement, addressing the individual sections in this systematic review and meta-analysis.[7]

Section and Topic	Item #	Checklist item	Location where item is reported
**TITLE**			
Title	1	Identify the report as a systematic review.	Page 1
**ABSTRACT**			
Abstract	2	See the PRISMA 2020 for Abstracts checklist.	Page 2
**INTRODUCTION**			
Rationale	3	Describe the rationale for the review in the context of existing knowledge.	Page 3
Objectives	4	Provide an explicit statement of the objective(s) or question(s) the review addresses.	Page 4
**METHODS**			
Eligibility criteria	5	Specify the inclusion and exclusion criteria for the review and how studies were grouped for the syntheses.	Page 5; Supplementary Table 3
Information sources	6	Specify all databases, registers, websites, organisations, reference lists and other sources searched or consulted to identify studies. Specify the date when each source was last searched or consulted.	Page 5; Supplementary Table 2
Search strategy	7	Present the full search strategies for all databases, registers and websites, including any filters and limits used.	Page 5; Supplementary Table 2
Selection process	8	Specify the methods used to decide whether a study met the inclusion criteria of the review, including how many reviewers screened each record and each report retrieved, whether they worked independently, and if applicable, details of automation tools used in the process.	Page 5
Data collection process	9	Specify the methods used to collect data from reports, including how many reviewers collected data from each report, whether they worked independently, any processes for obtaining or confirming data from study investigators, and if applicable, details of automation tools used in the process.	Page 5 to Page 6
Data items	10a	List and define all outcomes for which data were sought. Specify whether all results that were compatible with each outcome domain in each study were sought (e.g. for all measures, time points, analyses), and if not, the methods used to decide which results to collect.	Page 6
	10b	List and define all other variables for which data were sought (e.g. participant and intervention characteristics, funding sources). Describe any assumptions made about any missing or unclear information.	Page 6
Study risk of bias assessment	11	Specify the methods used to assess risk of bias in the included studies, including details of the tool(s) used, how many reviewers assessed each study and whether they worked independently, and if applicable, details of automation tools used in the process.	Page 6

Section and Topic	Item #	Checklist item	Location where item is reported
Effect measures	12	Specify for each outcome the effect measure(s) (e.g. risk ratio, mean difference) used in the synthesis or presentation of results.	Page 6
Synthesis methods	13a	Describe the processes used to decide which studies were eligible for each synthesis (e.g. tabulating the study intervention characteristics and comparing against the planned groups for each synthesis (item #5)).	Page 6
	13b	Describe any methods required to prepare the data for presentation or synthesis, such as handling of missing summary statistics, or data conversions.	Page 6 to Page 7
	13c	Describe any methods used to tabulate or visually display results of individual studies and syntheses.	Page 6
	13d	Describe any methods used to synthesize results and provide a rationale for the choice(s). If meta-analysis was performed, describe the model(s), method(s) to identify the presence and extent of statistical heterogeneity, and software package(s) used.	Page 6 to Page 8
	13e	Describe any methods used to explore possible causes of heterogeneity among study results (e.g. subgroup analysis, meta-regression).	Page 6 to Page 8
	13f	Describe any sensitivity analyses conducted to assess robustness of the synthesized results.	Page 6 to Page 8
Reporting bias assessment	14	Describe any methods used to assess risk of bias due to missing results in a synthesis (arising from reporting biases).	Page 6
Certainty assessment	15	Describe any methods used to assess certainty (or confidence) in the body of evidence for an outcome.	Page 6 to Page 8
**RESULTS**			
Study selection	16a	Describe the results of the search and selection process, from the number of records identified in the search to the number of studies included in the review, ideally using a flow diagram.	Page 68; Figure 1
	16b	Cite studies that might appear to meet the inclusion criteria, but which were excluded, and explain why they were excluded.	Page 68; Figure 1
Study characteristics	17	Cite each included study and present its characteristics.	Page 30 to Page 34; Table 1
Risk of bias in studies	18	Present assessments of risk of bias for each included study.	Page 22 to Page 23; Page 69; Figure 2
Results of individual studies	19	For all outcomes, present, for each study: (a) summary statistics for each group (where appropriate) and (b) an effect estimate and its precision (e.g. confidence/credible interval), ideally using structured tables or plots.	Page 11 to Page 22; Page 35 to 63, Tables 2 to 21; Page 76 to 77, Figure 6
Results of syntheses	20a	For each synthesis, briefly summarise the characteristics and risk of bias among contributing studies.	Page 9 to Page 10
	20b	Present results of all statistical syntheses conducted. If meta-analysis was done, present for each the summary estimate and its precision (e.g. confidence/credible interval) and measures of statistical heterogeneity. If	Page 22 to Page 23

Section and Topic	Item #	Checklist item	Location where item is reported
		comparing groups, describe the direction of the effect.	
	20c	Present results of all investigations of possible causes of heterogeneity among study results.	Page 11 to Page 22
	20d	Present results of all sensitivity analyses conducted to assess the robustness of the synthesized results.	Page 11 to Page 22
Reporting biases	21	Present assessments of risk of bias due to missing results (arising from reporting biases) for each synthesis assessed.	Page 68, Figure 2; Supplementary material; Table 4
Certainty of evidence	22	Present assessments of certainty (or confidence) in the body of evidence for each outcome assessed.	Supplementary material; Table 5
**DISCUSSION**			
Discussion	23a	Provide a general interpretation of the results in the context of other evidence.	Page 24 to Page 28
	23b	Discuss any limitations of the evidence included in the review.	Page 28
	23c	Discuss any limitations of the review processes used.	Page 28
	23d	Discuss implications of the results for practice, policy, and future research.	Page 29
**OTHER INFORMATION**			
Registration and protocol	24a	Provide registration information for the review, including register name and registration number, or state that the review was not registered.	Page 5
	24b	Indicate where the review protocol can be accessed, or state that a protocol was not prepared.	Page 5
	24c	Describe and explain any amendments to information provided at registration or in the protocol.	N/A
Support	25	Describe sources of financial or non-financial support for the review, and the role of the funders or sponsors in the review.	Page 1
Competing interests	26	Declare any competing interests of review authors.	Page 1
Availability of data, code and other materials	27	Report which of the following are publicly available and where they can be found: template data collection forms; data extracted from included studies; data used for all analyses; analytic code; any other materials used in the review.	Supplementary material; Table 6

Supplementary Table 2: The search strategy performed on 1st November 2021 is shown below outlining the respective databases, the search terms, publication dates chosen as limiting factors, and number of results from each database

Database	Search terms	Publication dates	Results (n)
Medline	normal pressure hydrocephalus.mp. [mp=ti, ab, hw, tn, ot, dm, mf, dv, kf, fx, dq, nm, ox, px, rx, an, ui, sy] limit 1 to yr="1965 - 2021"	1965 - 2021	n = 4,133
Embase	normal pressure hydrocephalus.mp. [mp=ti, ab, hw, tn, ot, dm, mf, dv, kf, fx, dq, nm, ox, px, rx, an, ui, sy] limit 1 to yr="1965 -2021"	1965 - 2021	n = 2,891
Scopus	normal AND pressure AND hydrocephalus AND response OR responsiveness OR responder OR predict AND (EXCLUDE (PUBYEAR , 2021) OR EXCLUDE (PUBYEAR , 2020) OR EXCLUDE (PUBYEAR , 2019) OR EXCLUDE (PUBYEAR , 2018) OR EXCLUDE (PUBYEAR , 2022))	1970 - 2021	n = 4,456
Pubmed	(normal pressure hydrocephalus) AND ((response) OR (responsiveness) OR (responder) OR (predict))	1971 - 2021	n = 1,024
Google Scholar	idiopathic normal pressure hydrocephalus shunt response predict marker	1965 - 2021	n = 5,850
JSTOR	Normal pressure hydrocephalus	1965 - 2021	n = 83

Supplementary Table 3: Inclusion and Exclusion criteria used when filtering studies based off search results (found in Supplementary Table 2).

Inclusion criteria	Exclusion criteria
• Published in the English language<br>• Peer-reviewed journals<br>• Adult patients with radiological confirmation of idiopathic normal pressure hydrocephalus<br>• Use of a cerebrospinal fluid shunt<br>• Functional grading pre-operatively and at least 3 months post operatively<br>• Radiological predictors of shunt response	• All non-English languages<br>• Commentaries, case series, case reports, narrative reviews, letters to editors, books<br>• Any animal studies and lab-based studies<br>• Studies with known cause of hydrocephalus without any idiopathic cases<br>• Studies not discussing shunt response<br>• Studies with no radiology and those discussing other predictors only

Supplementary Table 4: Risk of Bias assessment for all included studies against all domains of the ROBINS-1 tool.

Study	D1	D2	D3	D4	D5	D6	D7	D8	Weight
Yamada et al., 2013	SERIOUS	MODERATE	LOW	LOW	LOW	MODERATE	LOW	MODERATE	1
Ishii et al., 2011	LOW	LOW	LOW	LOW	LOW	LOW	LOW	LOW	1
Kazui et al,. 2013	LOW	LOW	LOW	LOW	LOW	LOW	LOW	LOW	1
Murakami et al., 2007	SERIOUS	LOW	LOW	LOW	LOW	MODERATE	LOW	MODERATE	1
Aoki et al., 2020	LOW	MODERATE	LOW	LOW	LOW	MODERATE	LOW	MODERATE	1
Jurcoane et al., 2013	LOW	MODERATE	LOW	LOW	LOW	SERIOUS	LOW	MODERATE	1
Peter Black, 1980	SERIOUS	LOW	LOW	LOW	LOW	MODERATE	LOW	MODERATE	1
Poca, 2004	SERIOUS	LOW	LOW	LOW	LOW	LOW	LOW	MODERATE	1
McGirt et al,. 2005	SERIOUS	MODERATE	LOW	LOW	LOW	SERIOUS	LOW	SERIOUS	1
Kawaguchi et al, 2011	LOW	LOW	LOW	LOW	LOW	LOW	LOW	LOW	1
Chen et al., 2008	MODERATE	LOW	LOW	LOW	LOW	LOW	LOW	LOW	1
Palm et al, 2006	LOW	MODERATE	LOW	LOW	LOW	MODERATE	LOW	MODERATE	1
Stecco et al., 2020	SERIOUS	LOW	LOW	LOW	LOW	MODERATE	LOW	MODERATE	1
Poca et al., 2002	SERIOUS	MODERATE	LOW	LOW	LOW	MODERATE	LOW	MODERATE	1
Agerskov et al., 2020	SERIOUS	LOW	LOW	LOW	LOW	MODERATE	LOW	MODERATE	1
Agerskov et al., 2019	SERIOUS	LOW	LOW	LOW	LOW	LOW	LOW	MODERATE	1
Narita et al., 2016	LOW	LOW	LOW	LOW	MODERATE	LOW	LOW	LOW	1
Virhammar et al., 2014 MRI - ALL	SERIOUS	LOW	LOW	LOW	LOW	LOW	LOW	MODERATE	1
Hong et al., 2018	SERIOUS	LOW	LOW	LOW	MODERATE	LOW	LOW	MODERATE	1
Garcia-Armengol et al., 2016	MODERATE	LOW	LOW	LOW	LOW	LOW	LOW	LOW	1
Ziegelitz et al., 2013	SERIOUS	LOW	LOW	LOW	LOW	MODERATE	LOW	MODERATE	1
Yamamoto et al., 2013	MODERATE	LOW	LOW	LOW	LOW	MODERATE	LOW	MODERATE	1
Mantovani et al, 2021	MODERATE	LOW	LOW	LOW	LOW	MODERATE	LOW	MODERATE	1
Wu et al, 2021	MODERATE	LOW	LOW	LOW	LOW	MODERATE	LOW	MODERATE	1
Kuchcinski et al, 2019	LOW	LOW	LOW	LOW	LOW	LOW	LOW	LOW	1
Shinoda et al, 2017	LOW	MODERATE	LOW	LOW	LOW	MODERATE	LOW	MODERATE	1

Virhammar et al., 2014 CA ONLY	LOW	LOW	LOW	LOW	LOW	LOW	LOW	LOW	1
Grahnke et al., 2018	LOW	LOW	LOW	LOW	LOW	MODERATE	LOW	LOW	1

Supplementary Table 5: Level of evidence of each of the included studies based on the Oxford Centre of Evidence-Based Medicine (OCEBM) Levels of Evidence

Study number	Author, Year	Level of evidence
Study 1	Yamada et al., 2013	2b
Study 2	Ishii et al., 2011	1b
Study 3	Kazui et al,. 2013	2b
Study 4	Murakami et al., 2007	3b
Study 5	Aoki et al., 2020	2b
Study 6	Jurcoane et al., 2013	2b
Study 7	Peter Black, 1980	2b
Study 8	Poca, 2004	3b
Study 9	McGirt et al,. 2005	2b
Study 10	Kawaguchi et al, 2011	1b
Study 11	Chen et al., 2008	1b
Study 12	Palm et al, 2006	2b
Study 13	Stecco et al., 2020	2b
Study 14	Poca et al., 2002	2b
Study 15	Agerskov et al., 2020	2b
Study 16	Agerskov et al., 2019	1b
Study 17	Narita et al., 2016	3b
Study 18	Virhammar et al., 2014	2b
Study 19	Hong et al., 2018	1b
Study 20	Garcia-Armengol et al., 2016	2b
Study 21	Ziegelitz et al., 2013	3b
Study 22	Yamamoto et al., 2013	3b
Study 23	Mantovani et al, 2021	2b
Study 24	Wu et al, 2021	2b
Study 25	Kuchcinski et al, 2019	3b
Study 26	Shinoda et al, 2017	2b
Study 27	Virhammar et al., 2014	2b
Study 28	Grahnke et al., 2018	2b

Supplementary Table 6: The R code that was utilised for the meta-analysis and associated figures, ROB graphs, as well as the meta-regression

```
#Install relevant packages

install.packages(c("robumeta", "metafor", "dplyr"))


install.packages("meta")

install.packages("dmetar")

install.packages("readxl")

#into library load

library("robumeta")

library("metafor")

library("dplyr")

library("mada")

library("meta")

library("dmetar")

library("readxl")
```

```r
#install updated packaged for Rtools required now (2022)

write('PATH="${RTOOLS40_HOME}\\usr\\bin;${PATH}"', file = "~/.Renviron", append = TRUE)

Sys.which("make")

## "C:\\rtools40\\usr\\bin\\make.exe"

install.packages("jsonlite", type = "source")


#Install relevant packages

install.packages(c("robumeta", "metafor", "dplyr"))

install.packages("meta")

install.packages("devtools")



install.packages("readxl")

install.packages("devtools")

install_github("mcguinlu/robvis")

install.packages("robvis")

install.packages("utf8")
```

```
#into library load
library("robumeta")
library("metafor")
library("dplyr")
library("mada")
library("meta")
library("dmetar")
library("readxl")
library("robvis")
library("utf8")



#ROB analysis
#loading excel sheet

NPHBio_Risk_of_bias_NR <- read_excel("~/Desktop/Radiological NPH ROB NEW.xlsx")
```

```
NPHBio_Risk_of_bias_NR


#running Risk of bias analysis


rob_summary(NPHBio_Risk_of_bias_NR, tool = "ROBINS-I", weighted = FALSE , overall = TRUE)

######################## DESH ########################################


#load Excel file

library(readxl)
```

```
DOR_DESH_NEW <- read_excel("Desktop/iNPH Radio New/Relevant stuff/Tables/DESH/DESH NEW.xlsx")



#meta-analysis
meta_Radio_DESH_NEW <- metagen(TE = DOR_DESH_NEW$TE,
                               seTE = DOR_DESH_NEW$seTE,
                               studlab = DOR_DESH_NEW$Author,
                               title = "DESH",
                               data = DOR_DESH_NEW,
                               sm = "",
                               fixed = FALSE,
                               random = TRUE,
                               method.tau = "DL",
                               lower = DOR_DESH_NEW$lower,
                               upper = DOR_DESH_NEW$upper,
                               pval = DOR_DESH_NEW$pval,
                               hakn = TRUE,
```

```
                              n.e = DOR_DESH_NEW$Sample,
                              text.random = "Pooled Odds Ratio",
                              label.e = "",
                              prediction = TRUE,
                              backtransf = TRUE)

forest.meta(meta_Radio_DESH_NEW,
            lab.e = "",
            JAMA.pval = FALSE,
            test.overall.random = TRUE)



#DESH analysis 2 for heterogeneity

library(readxl)
DOR_DESH_NEW2 <- read_excel("Desktop/iNPH Radio New/Relevant stuff/Tables/DESH/DESH NEW 2.xlsx")
```

```
#meta-analysis
meta_Radio_DESH_NEW2 <- metagen(TE = DOR_DESH_NEW2$TE,
                               seTE = DOR_DESH_NEW2$seTE,
                               studlab = DOR_DESH_NEW2$Author,
                               title = "DESH",
                               data = DOR_DESH_NEW2,
                               sm = "",
                               fixed = FALSE,
                               random = TRUE,
                               method.tau = "DL",
                               lower = DOR_DESH_NEW2$lower,
                               upper = DOR_DESH_NEW2$upper,
                               pval = DOR_DESH_NEW2$pval,
                               hakn = TRUE,
                               n.e = DOR_DESH_NEW2$Sample,
                               text.random = "Pooled Odds Ratio",
                               label.e = "",
```

```
                                prediction = TRUE,
                                backtransf = TRUE)

forest.meta(meta_Radio_DESH_NEW2,
            lab.e = "",
            JAMA.pval = FALSE,
            test.overall.random = TRUE)
#meta-regression for DESH metareg: only if studies >3, single ones (all)

meta_Radio_DESH_Sample<- metareg(meta_Radio_DESH_NEW,
                                 ~ Sample)
print(meta_Radio_DESH_Sample)



meta_Radio_DESH_Year<- metareg(meta_Radio_DESH_NEW,
                                  ~ Year)
print(meta_Radio_DESH_Year)
```

```
meta_Radio_DESH_Age <- metareg(meta_Radio_DESH_NEW,
                            ~ Age)
print(meta_Radio_DESH_Age)


#note females is significant regression factor!
meta_Radio_DESH_Females <- metareg(meta_Radio_DESH_NEW,
                               ~ Females)
print(meta_Radio_DESH_Females)


meta_Radio_DESH_HTN <- metareg(meta_Radio_DESH_NEW,
                              ~ HTN)
print(meta_Radio_DESH_HTN)


meta_Radio_DESH_Gait<- metareg(meta_Radio_DESH_NEW,
                               ~ Gait)
print(meta_Radio_DESH_Gait)
```

```
meta_Radio_DESH_mRS<- metareg(meta_Radio_DESH_NEW,
                           ~ mRS)
print(meta_Radio_DESH_mRS)


meta_Radio_DESH_EI<- metareg(meta_Radio_DESH_NEW,
                              ~ EI)
print(meta_Radio_DESH_EI)

meta_Radio_DESH_CA<- metareg(meta_Radio_DESH_NEW,
                             ~ CA)
print(meta_Radio_DESH_CA)

#DESH is significant regression factor!
meta_Radio_DESH_DESH<- metareg(meta_Radio_DESH_NEW,
                             ~ DESH)
print(meta_Radio_DESH_DESH)
```

```
meta_Radio_DESH_SR<- metareg(meta_Radio_DESH_NEW,
                               ~ SR)
print(meta_Radio_DESH_SR)


meta_Radio_DESH_Comp <- metareg(meta_Radio_DESH_NEW,
                             ~ Complic.)
print(meta_Radio_DESH_Comp)


meta_Radio_DESH_Comp <- metareg(meta_Radio_DESH_NEW,
                                ~ Complic. + DESH + SR + CA + EI + mRS + Gait + HTN)
print(meta_Radio_DESH_Comp)


#categorical variables: imaging plane and modality
```

```
meta_Radio_DESH_Imagingplane <- metareg(meta_Radio_DESH_NEW,
                                        ~ 0 + factor(Imagingplane))
print(meta_Radio_DESH_Imagingplane)


meta_Radio_DESH_Imagingmodality <- metareg(meta_Radio_DESH_NEW,
                                        ~ 0 + factor(Imagingmodality))
print(meta_Radio_DESH_Imagingmodality)


######################## Periventricular WM ########################################




#load Excel file

library(readxl)
```

```
DOR_PVWM_NEW <- read_excel("Desktop/iNPH Radio New/Relevant stuff/Tables/PVWM/Periventricular WM NEW.xlsx")

#meta-analysis

meta_Radio_PVWM_NEW <- metagen(TE = DOR_PVWM_NEW$TE,
                               seTE = DOR_PVWM_NEW$seTE,
                               studlab = DOR_PVWM_NEW$Author,
                               title = "DESH",
                               data = DOR_PVWM_NEW,
                               sm = "",
                               fixed = FALSE,
                               random = TRUE,
                               method.tau = "DL",
                               lower = DOR_PVWM_NEW$lower,
                               upper = DOR_PVWM_NEW$upper,
                               pval = DOR_PVWM_NEW$pval,
                               hakn = TRUE,
```

```
                              n.e = DOR_PVWM_NEW$Sample,
                              text.random = "Pooled Odds Ratio",
                              label.e = "",
                              prediction = TRUE)

forest.meta(meta_Radio_PVWM_NEW,
            label.e = "",
            JAMA.pval = FALSE,
            test.overall.random = TRUE)



meta_Radio_PVWM_Sample <- metareg(meta_Radio_PVWM_NEW,
                                  ~ Sample)
print(meta_Radio_PVWM_Sample)


meta_Radio_PVWM_Age <- metareg(meta_Radio_PVWM_NEW,
                               ~ Age)
```

```
print(meta_Radio_PVWM_Age)


meta_Radio_PVWM_Females <- metareg(meta_Radio_PVWM_NEW,
                                   ~ Females)
print(meta_Radio_PVWM_Females)


meta_Radio_PVWM_HTN <- metareg(meta_Radio_PVWM_NEW,
                               ~ HTN)
print(meta_Radio_PVWM_HTN)

meta_Radio_PVWM_Gait <- metareg(meta_Radio_PVWM_NEW,
                                ~ Gait)
print(meta_Radio_PVWM_Gait)
```

```
meta_Radio_PVWM_SR <- metareg(meta_Radio_PVWM_NEW,
                                ~ SR)
print(meta_Radio_PVWM_SR)



meta_Radio_PVWM_Comp <- metareg(meta_Radio_PVWM_NEW,
                                ~ Complic.)
print(meta_Radio_PVWM_Comp)


meta_Radio_PVWM_Year <- metareg(meta_Radio_PVWM_NEW,
                                ~ Year)
print(meta_Radio_PVWM_Year)

#categorical variables: imaging plane and modality
```

```
meta_Radio_PVWM_Imagingplane <- metareg(meta_Radio_PVWM_NEW,
                                        ~ 0 + factor(Imagingplane))
print(meta_Radio_PVWM_Imagingplane)


meta_Radio_DESH_Imagingmodality <- metareg(meta_Radio_DESH_NEW,
                                           ~ 0 + factor(Imagingmodality))
print(meta_Radio_DESH_Imagingmodality)



######################## Callosal Angle ########################################




#load Excel file

library(readxl)
```

```
DOR_CA_NEW <- read_excel("Desktop/iNPH Radio New/Relevant stuff/Tables/Callosal Angle/CA
NEW.xlsx")



#meta-analysis

meta_Radio_CA_NEW <- metagen(TE = DOR_CA_NEW$TE,
                             seTE = DOR_CA_NEW$seTE,
                             studlab = DOR_CA_NEW$Author,
                             title = "DESH",
                             data = DOR_CA_NEW,
                             sm = "",
                             fixed = FALSE,
                             random = TRUE,
                             method.tau = "DL",
                             lower = DOR_CA_NEW$lower,
                             upper = DOR_CA_NEW$upper,
                             pval = DOR_CA_NEW$pval,
                             hakn = TRUE,
                             n.e = DOR_CA_NEW$Sample,
```

```
                                text.random = "Pooled Odds Ratio",
                                label.e = "",
                                prediction = TRUE)

forest.meta(meta_Radio_CA_NEW,
            lab.e = "",
            JAMA.pval = FALSE,
            test.overall.random = TRUE)



#regression

meta_Radio_CA_Sample <- metareg(meta_Radio_CA_NEW,
                                     ~ Sample)
print(meta_Radio_CA_Sample)



meta_Radio_CA_Age <- metareg(meta_Radio_CA_NEW,
                                  ~ Age)
```

```
print(meta_Radio_CA_Age)



meta_Radio_CA_Females <- metareg(meta_Radio_CA_NEW,
                                  ~ Females)
print(meta_Radio_CA_Females)



meta_Radio_CA_HTN <- metareg(meta_Radio_CA_NEW,
                              ~ HTN)
print(meta_Radio_CA_HTN)


meta_Radio_CA_Depression <- metareg(meta_Radio_CA_NEW,
                             ~ Depression)
print(meta_Radio_CA_Depression)
```

```
meta_Radio_CA_Gait <- metareg(meta_Radio_CA_NEW,
                                ~ Gait)
print(meta_Radio_CA_Gait)


meta_Radio_CA_CA <- metareg(meta_Radio_CA_NEW,
                              ~ CA)
print(meta_Radio_CA_CA)



meta_Radio_CA_SR <- metareg(meta_Radio_CA_NEW,
                            ~ SR)
print(meta_Radio_CA_SR)


meta_Radio_CA_SR <- metareg(meta_Radio_CA_NEW,
                            ~ Year)
print(meta_Radio_CA_SR)
```

```
#categorical variables: imaging plane and modality

meta_Radio_CA_Imagingplane <- metareg(meta_Radio_CA_NEW,
                                       ~ imagingplane)
print(meta_Radio_CA_Imagingplane)

meta_Radio_CA_Imagingmodality <- metareg(meta_Radio_CA_NEW,
                                          ~ 0 + factor(imagingmodality))
print(meta_Radio_CA_Imagingmodality)

######################## CBF ########################################

#load Excel file
```

```
library(readxl)

DOR_CBF_NEW <- read_excel("Desktop/iNPH Radio New/Relevant stuff/Tables/Cerebral blood
flow/Cerebral blood flow NEW1 .xlsx")
```

```
#meta-analysis

meta_Radio_CBF_NEW <- metagen(TE = DOR_CBF_NEW$TE,

                              seTE = DOR_CBF_NEW$seTE,

                              studlab = DOR_CBF_NEW$Author,

                              title = "CBF",

                              data = DOR_CBF_NEW,

                              sm = "",

                              fixed = FALSE,

                              random = TRUE,

                              method.tau = "DL",

                              lower = DOR_CBF_NEW$lower,

                              upper = DOR_CBF_NEW$upper,

                              pval = DOR_CBF_NEW$pval,
```

```
                              hakn = TRUE,
                              n.e = DOR_CBF_NEW$Sample,
                              text.random = "Pooled Odds Ratio",
                              label.e = "",
                              prediction = TRUE)

forest.meta(meta_Radio_CBF_NEW,
            label.e = "",
            JAMA.pval = FALSE,
            test.overall.random = TRUE)



#regression

meta_Radio_CBF_Sample <- metareg(meta_Radio_CBF_NEW,
                                  ~ Sample)
print(meta_Radio_CBF_Sample)
```

```
meta_Radio_CBF_Age <- metareg(meta_Radio_CBF_NEW,
                                 ~ Age)
print(meta_Radio_CBF_Age)
```

```
meta_Radio_CBF_Females <- metareg(meta_Radio_CBF_NEW,
                                 ~ Females)
print(meta_Radio_CBF_Females)
```

```
meta_Radio_CBF_MMSE <- metareg(meta_Radio_CBF_NEW,
                             ~ MMSE)
print(meta_Radio_CBF_MMSE)
```

```
meta_Radio_CBF_SR <- metareg(meta_Radio_CBF_NEW,
                              ~ SR)
print(meta_Radio_CBF_SR)


meta_Radio_CBF_Year <- metareg(meta_Radio_CBF_NEW,
                                ~ Year)
print(meta_Radio_CBF_Year)



#categorical variables: imaging plane and modality


meta_Radio_CBF_Imagingplane <- metareg(meta_Radio_CBF_NEW,
                                        ~ Imagingplane)
print(meta_Radio_CBF_Imagingplane)
```

```
meta_Radio_CBF_Imagingmodality <- metareg(meta_Radio_CBF_NEW,
                                          ~ 0 + factor(imagingmodality))
print(meta_Radio_CBF_Imagingmodality)

####################### CTC ########################################

#load Excel file

library(readxl)
DOR_CTC_NEW <- read_excel("Desktop/iNPH Radio New/Tables/CTC/CTC NEW.xlsx")
View(DOR_CTC_NEW)



#meta-analysis
meta_Radio_CTC_NEW <- metagen(TE = DOR_CTC_NEW$TE,
                              seTE = DOR_CTC_NEW$seTE,
                              studlab = DOR_CTC_NEW$Author,
```

```
                              title = "CTC",
                              data = DOR_CTC_NEW,
                              sm = "",
                              fixed = FALSE,
                              random = TRUE,
                              method.tau = "DL",
                              lower = c(0.250,
                                        0.046,
                                        0.911),
                              upper = DOR_CTC_NEW$upper,
                              pval = DOR_CTC_NEW$pval,
                              hakn = TRUE,
                              n.e = DOR_CTC_NEW$Sample,
                              text.random = "Pooled Odds Ratio",
                              label.e = "",
                              prediction = TRUE)

forest.meta(meta_Radio_CTC_NEW,
            lab.e = "",
```

```
            JAMA.pval = FALSE,
            test.overall.random = TRUE)




####################### Study characteristics graphs ##################

library(readxl)
Heatmap <- read_excel("Desktop/iNPH Radio New/Tables/Qualitative graphs/Heatmap.xlsx")
View(Heatmap)

####### 1. Make bar plot for study design

studydesigntable <- table(Heatmap$`Study design`)
```

```
# Load ggplot2
library(ggplot2)

# Create data


data1 <- data.frame(
  name=c("Prospective", "Retrospective") ,
  value=c(19, 9)
)




barplot(height = data1$value,
        names.arg = data1$name,
        xlab = "Study design",
        ylab = "Number of studies",
        ylim = c(0,20), xpd = TRUE,
```

```
        col="black", angle = c(45, 90), density = c(10,30))




###### 2. Make bar plot for study sample size

library(readxl)
Heatmap_Final_April_ <- read_excel("Desktop/iNPH Radio New/Tables/Heatmap Final April .xlsx")
View(Heatmap_Final_April_)

data2 <- data.frame(
  name=Heatmap_Final_April_$Number,
  value=Heatmap_Final_April_$Sample
)

barplot(height = data2$value,
        names.arg = data2$name,
        xlab = "Study",
```

```
        ylab = "Sample size",

        ylim = c(0, 200),

        col = "black",

        xpd = TRUE)




###### 3. Make bar plot for study year



studysamplesizetable <- table(Heatmap$date)


data3 <- data.frame(

  name=c("1980", "2002", "2004", "2005", "2006", "2007", "2008",

         "2011", "2013",  "2014",  "2016","2017", "2018", "2019", "2020", "2021"),

  value=c( 1,1,1,1,1,1,1,3,

           3,3,2,1,2,2,3,2)

 )
```

```
barplot(height = data3$value,
        names.arg = data3$name,
        xlab = "Year of study publication",
        ylab = "Number of studies",
        col = "black",
        ylim = NULL, xpd = TRUE)



library(RColorBrewer)
coul3 <- brewer.pal(9, "Spectral")

####################### Imaging characteristics graphs ##################

#Imaging modality

studyradiotable <- table(Heatmap$`Imaging modality`)
```

```
data4 <- data.frame(
  name=c( "1.5T MRI", "1.5T or 0.5T MRI", "3T MRI",
                    "0.5-3T MRI", "Undefined MRI", "CTC",
                    "CT or MRI", "SPECT", "PEG"),
  value=c(8, 1,5,2,1,2,4,2,3)
)

library(RColorBrewer)
coul <- brewer.pal(11, "PiYG")



barplot(height = data4$value,
        names.arg = data4$name,
        xlab = "Imaging modality",
        ylab = "Number of studies",
        col = c("palegreen", "seagreen1", "seagreen2", "seagreen3", "seagreen4",
                "slateblue1", "plum2", "blue", "salmon"),
        ylim = NULL, xpd = TRUE)
```

```
###################### Patient characteristics: Percentage ##################

# Mean patient  characteristics in percentages
data5 <- data.frame(
  name=c("Female","HTN", "DM", "Cognition-","Urine-", "HTriad+", "S-R", "Complic."),
value=c(0.418, 0.503, 0.279, 0.766, 0.666, 0.663, 0.736, 0.126))

library(RColorBrewer)

coul <- brewer.pal(7, "OrRd")


barplot(height = data5$value,
        names.arg = data5$name,
        xlab = "Patient characteristics",
        ylab = "Mean proportion",
        col = coul,
        ylim = c(0,1), xpd = TRUE)
```

```
# Mean clinical score absolute values

coul2 <- brewer.pal(5, "Greens")

data6 <- data.frame(
  name=c("MMSE", "EI", "mRS", "TUG",
         "CA"),
value=c(21.9, 0.4, 2.4, 19.7,
        76.6))



bp <- barplot(height = data6$value,
        names.arg = data6$name,
        xlab = "Patient characteristics",
        ylab = "Mean bsolute values",
        col = coul2,
```

```
        ylim = c(0,80), xpd = TRUE,
        )
text(bp, 0, data6$value,cex=2, pos=3, col ="black")




library(RColorBrewer)


coul <- brewer.pal(9, "Greys")



 my_bar <- barplot(height = data6$value,
        names.arg = data6$name,
        xlab = "Patient characteristics",
        ylab = "Mean value",
        col = coul,
        ylim = NULL, xpd = TRUE)
```

```
###################### Complex heatmap #################

library(readxl)
Heatmap_Final_April_ <- read_excel("Desktop/iNPH Radio New/Tables/Heatmap Final April .xlsx")
View(Heatmap_Final_April_)

# transform into matrix
HeatmapCool3 <- data.matrix(Heatmap2)

#replace NA by 0
is.na(HeatmapCool3) <- sapply(HeatmapCool3, is.infinite)
HeatmapCool3[is.na(HeatmapCool3)] <- 100


rownames(HeatmapCool3) <- c("Agerskov et al. (2019)",
                            "Agerskov et al. (2020)",
                            "Aoki et al. (2020)",
                            "Chen et al. (2008)",
```

"Garcia-Armengol et al. (2016)",

"Grahnke et al. (2018)",

"Hong et al. (2018)",

"Ishii et al. (2011)",

"Jurcoane et al. (2013)",

"Kawaguchi et al. (2011)",

"Kazui et al. (2013)",

"Kuchcinski et al. (2019)",

"Mantovani et al. (2021) ",

"McGirt et al. (2005)",

"Murakami et al. (2007)",

"Narita et al. (2016)",

"Palm et al. (2006)",

"PM Black (1980)",

"Poca et al. (2002)",

"Poca et at. (2004)",

"Shinoda et al. (2017)",

"Stecco et al. (2020)",

"Virhammar et al. (2014) CA",

```
                            "Virhammar et al. (2014)",
                            "Wu et al. (2021)",
                            "Yamada et al. (2013)",
                            "Yamamoto et al. (2013)",
                            "Ziegelitz et al. (2014)")



print(HeatmapCool3)



#print heatmap: THE MAGIC
heatmap(HeatmapCool3,
        col = heat.colors(10000),
        scale = "column",
```

```
        margins = c(6,6)
)


legend(x=0.98, y=0.07, bty = "n", cex = 0.6,
       border = "white", legend=c("min", "min-med","med/NA", "med-max",
"max"),fill=heat.colors(5))



################ impute missing data: MICE ######################


install.packages("mice")
install.packages("devtools")
devtools::install_github(repo = "amices/mice")
library("mice")
library("devtools")
library("usethis")
#load cleaned dataset with atleast 4+ studies covering the variable


library(readxl)
```

```
Heatmap_3 <- read.csv("Desktop/iNPH Radio New/Relevant stuff/Tables/Qualitative
graphs/Heatmap3NEW.csv")

Heatmap_3_DF <- as.data.frame(Heatmap_3)

imputed_Heatmap_3 <- mice(Heatmap_3)

summary(imputed_Heatmap_3)

final_imp_HP <- complete(imputed_Heatmap_3, 5)

final_imp_HP

#problem: mice cannot predict perfectly colinear variables (super sparse data), so need to find
and remove those columns

mice:::find.collinear(Heatmap_3)

complete(imputed_Heatmap_3)
```

```
imputed_Heatmap_3$imp$C



#try again with new clean dataset

library(readxl)

Heatmap_3_Cleaned <- read_excel("Desktop/iNPH Radio New/Relevant stuff/Tables/Qualitative graphs/Heatmap Cleaned Noncolinear.xlsx")


mice:::find.collinear(Heatmap_3_Cleaned)


complete(imputed_Heatmap_3_Cleaned)


imputed_Heatmap_3_Cleaned$imp$C


Heatmap_3_DF <- as.data.frame(Heatmap_3_Cleaned)


imputed_Heatmap_4 <- mice(Heatmap_3_Cleaned_DF)


summary(imputed_Heatmap_4)
```

```
 final_imp_HP_June <- complete(imputed_Heatmap_4, 5)

 final_imp_HP_June

imputed_HP_matrix <- as.matrix(final_imp_HP_June)

write.csv(imputed_Heatmap_matrix, file = "imputedheatmapjune.csv")

#import imputed heatmap

imputedheatmap <- read_excel("Desktop/iNPH Radio New/Tables/Qualitative
graphs/imputedheatmap.xlsx")

#prep heatmap with imputed data by turning it into DF

# transform into matrix
ImputedHeatmapDF <- data.matrix(imputedheatmap)
```

```
rownames(ImputedHeatmapDF) <- c("Agerskov et al. (2019)",
                             "Agerskov et al. (2020)",
                             "Aoki et al. (2020)",
                             "Chen et al. (2008)",
                             "Garcia-Armengol et al. (2016)",
                             "Grahnke et al. (2018)",
                             "Hong et al. (2018)",
                             "Ishii et al. (2011)",
                             "Jurcoane et al. (2013)",
                             "Kawaguchi et al. (2011)",
                             "Kazui et al. (2013)",
                             "Kuchcinski et al. (2019)",
                             "Mantovani et al. (2021) ",
                             "McGirt et al. (2005)",
                             "Murakami et al. (2007)",
                             "Narita et al. (2016)",
                             "Palm et al. (2006)",
                             "PM Black (1980)",
```

```
                            "Poca et al. (2002)",
                            "Poca et at. (2004)",
                            "Shinoda et al. (2017)",
                            "Stecco et al. (2020)",
                            "Virhammar et al. (2014) CA",
                            "Virhammar et al. (2014)",
                            "Wu et al. (2021)",
                            "Yamada et al. (2013)",
                            "Yamamoto et al. (2013)",
                            "Ziegelitz et al. (2014)")


#print heatmap: THE MAGIC
heatmap(ImputedHeatmapDF,
        col = cm.colors(1000000),
        scale = "column",
        margins = c(5,5)
)
```

```
legend(x=0.95, y=0.099, bty = "n", cex = 0.6,

       border = "black", legend=c("min", "min-med","med", "med-max", "max"),fill=cm.colors(5))




######### sensitivity analysis ############




# Load Total set dataset from dmetar (or download and open manually)


library(readxl)

iNPH_Radio_Sens <- read_excel("Desktop/iNPH Radio New/Tables/iNPH Total for Sensitivity
Analysis.xlsx")



# Use metcont to pool results.


meta_Total_Radio <- metagen(iNPH_Radio_Sens$TE,
```

```
iNPH_Radio_Sens$seTE,
data = iNPH_Radio_Sens,
 subset = NULL,
 exclude = NULL,
 id = NULL,
 sm = "",
 level = gs("level"),
 level.ma = gs("level.ma"),
 fixed = gs("fixed"),
 hakn = gs("hakn"),
 adhoc.hakn = gs("adhoc.hakn"),
 method.tau = gs("method.tau"),
 method.tau.ci = gs("method.tau.ci"),
 tau.preset = NULL,
 TE.tau = NULL,
 tau.common = gs("tau.common"),
 detail.tau = "",
 prediction = gs("prediction"),
 level.predict = gs("level.predict"),
```

```
null.effect = 0,
method.bias = gs("method.bias"),
n.e = NULL,
n.c = NULL,
iNPH_Radio_Sens$pval,
level.ci = 0.95,
method.mean = "Luo",
method.sd = "Shi",
backtransf = gs("backtransf"),
pscale = 1,
irscale = 1,
irunit = "person-years",
text.fixed = gs("text.fixed"),
text.random = gs("text.random"),
text.predict = gs("text.predict"),
text.w.fixed = gs("text.w.fixed"),
text.w.random = gs("text.w.random"),
title = gs("title"),
complab = gs("complab"),
```

```
        outclab = "",
        label.e = gs("label.e"),
        label.c = gs("label.c"),
        label.left = gs("label.left"),
        label.right = gs("label.right")
)

#numerical visualisation of meta_ttau (note that SR are the experimental group so reference group, so minus result means it is less in them)

print(meta_Total_Radio)

#make the plot

forest.meta(meta_Total_Radio,
            sortvar = TE,
            predict = TRUE,
            lab.e = "",
            lab.c = "",
            JAMA.pval = FALSE,
```

```
            test.overall.random = TRUE,
            label.test.overall.random = "Overall statistical result of model: ")



#Eggers (Publication bias) calculation

eggersplot <- metabias(
  meta_Total_Radio,
  method.bias = meta_Total_Radio$method.bias,
  plotit = TRUE,
  correct = FALSE,
  k.min = 1
)

print(eggersplot)

print(eggersplot,
      digits = gs("digits"),
```

```
      digits.stat = gs("digits.stat"),
      digits.pval = max(gs("digits.pval"), 2),
      digits.se = gs("digits.se"),
      digits.tau2 = gs("digits.tau2"),
      scientific.pval = gs("scientific.pval"),
      big.mark = gs("big.mark"),
      zero.pval = gs("zero.pval"),
      JAMA.pval = gs("JAMA.pval"),
      text.tau2 = gs("text.tau2"))



#Eggers (Publication bias) plot


eggersplot2 <-metabias(
  meta_Total_Radio,
  method.bias = meta_Total_Radio$method.bias,
```

```
  plotit = TRUE,
  correct = FALSE,
  k.min = 1
)

funnel.meta(meta_Total_Radio,
            xlim = c(-100, 100),
            studlab = FALSE,
            method.bias = "linreg")

################ correlation plot ##############

library(readxl)
Correlation_table <- read_excel("Desktop/iNPH Radio New/nonimputedheatmap.xlsx")

install.packages("corrplot")
library("corrplot")

CorRadio <- cor(Correlation_table, use="pairwise.complete.obs")
```

```
#option 1
testRes = cor.mtest(Correlation_table, conf.level = 0.95)

corrplot(CorRadio, p.mat = testRes$p, method = 'circle', type = 'lower', insig='blank',
         addCoef.col ='black', number.cex = 0.7, order = 'AOE', diag=FALSE)

#option 2
corrplot(CorRadio, p.mat = testRes$p, method = 'color', diag = FALSE, type = 'upper',
         sig.level = c(0.001, 0.01, 0.05), pch.cex = 0.9, number.cex = 0.7, tl.col="black",
         insig = 'label_sig', pch.col = 'grey20', order = 'AOE', col = COL2('RdYlBu'))

#option 2
CorRadio <- cor(CorRadio, use="pairwise.complete.obs")

corrplot(CorRadio2, p.mat = testRes$p, method = 'color', diag = FALSE, type = 'upper',
         sig.level = c(0.001, 0.01, 0.05), pch.cex = 0.9, number.cex = 0.7, tl.col="black",
         insig = 'label_sig', pch.col = 'grey20', order = 'AOE', col = COL2('RdYlBu'))
```

```
install.packages("PerformanceAnalytics")
library(PerformanceAnalytics)

install.packages("psych")
library(psych)

corpl(CorRadio$Age, CorRadio$EI)

pairs.panels(CorRadio,
             smooth = TRUE,      # If TRUE, draws loess smooths
             scale = FALSE,      # If TRUE, scales the correlation text font
             density = TRUE,     # If TRUE, adds density plots and histograms
             ellipses = TRUE,    # If TRUE, draws ellipses
             method = "pearson", # Correlation method (also "spearman" or "kendall")
             pch = 21,           # pch symbol
             lm = FALSE,         # If TRUE, plots linear fit rather than the LOESS (smoothed) fit
             cor = TRUE,         # If TRUE, reports correlations
             jiggle = FALSE,     # If TRUE, data points are jittered
             factor = 2,         # Jittering factor
```

```
            hist.col = 4,       # Histograms color
            stars = TRUE,       # If TRUE, adds significance level with stars
            ci = TRUE)          # If TRUE, adds confidence intervals


install.packages("car")
library(car)
scatterplotMatrix(~ Sample + Age + Females + DM + HTN + Gaitdef + Cognitiondef + Urinedef + MMSE +
EI + CA + SR + Complic, data = CorRadio,
                  diagonal = FALSE,             # Remove kernel density estimates
                  regLine = list(col = "green", # Linear regression line color
                                 lwd = 3),      # Linear regression line width
                  smooth = list(col.smooth = "red",   # Non-parametric mean color
                                col.spread = "blue",
                                legend.plot = TRUE)) # Non-parametric variance color
```

References

[1] Agerskov S, Wallin M, Hellstrom P, Ziegelitz D, Wikkelso C, Tullberg M (2019) Absence of Disproportionately Enlarged Subarachnoid Space Hydrocephalus, a Sharp Callosal Angle, or Other Morphologic MRI Markers Should Not Be Used to Exclude Patients with Idiopathic Normal Pressure Hydrocephalus from Shunt Surgery. AJNR Am J Neuroradiol 40:74-79. doi:10.3174/ajnr.A5910

[2] Garcia-Armengol R, Domenech S, Botella-Campos C, Goncalves FJ, Menendez B, Teixidor P, Munoz-Narbona L, Rimbau J (2016) Comparison of elevated intracranial pressure pulse amplitude and disproportionately enlarged subarachnoid space (DESH) for prediction of surgical results in suspected idiopathic normal pressure hydrocephalus. Acta Neurochir (Wien) 158:2207-2213. doi:10.1007/s00701-016-2858-5

[3] Grahnke K, Jusue-Torres I, Szujewski C, Joyce C, Schneck M, Prabhu VC, Anderson DE (2018) The Quest for Predicting Sustained Shunt Response in Normal-Pressure Hydrocephalus: An Analysis of the Callosal Angle's Utility. World Neurosurgery 115:e717-e722. doi:https://doi.org/10.1016/j.wneu.2018.04.150

[4] Hong YJ, Kim MJ, Jeong E, Kim JE, Hwang J, Lee JI, Lee JH, Na DL (2018) Preoperative biomarkers in patients with idiopathic normal pressure hydrocephalus showing a favorable shunt surgery outcome. J Neurol Sci 387:21-26. doi:10.1016/j.jns.2018.01.017

[5] Ishii K, Hashimoto M, Hayashida K, Hashikawa K, Chang CC, Nakagawara J, Nakayama T, Mori S, Sakakibara R (2011) A multicenter brain perfusion SPECT study evaluating idiopathic normal-pressure hydrocephalus on neurological improvement. Dement Geriatr Cogn Disord 32:1-10. doi:10.1159/000328972

[6] Kazui H, Mori E, Ohkawa S, Okada T, Kondo T, Sakakibara R, Ueki O, Nishio Y, Ishii K, Kawaguchi T, Ishikawa M, Takeda M (2013) Predictors of the disappearance of triad symptoms in patients with idiopathic normal pressure hydrocephalus after shunt surgery. J Neurol Sci 328:64-69. doi:10.1016/j.jns.2013.02.020

[7] Page MJ, McKenzie JE, Bossuyt PM, Boutron I, Hoffmann TC, Mulrow CD, Shamseer L, Tetzlaff JM, Akl EA, Brennan SE, Chou R, Glanville J, Grimshaw JM, Hróbjartsson A, Lalu MM, Li T, Loder EW, Mayo-Wilson E, McDonald S, McGuinness LA, Stewart LA, Thomas J, Tricco AC, Welch VA, Whiting P, Moher D (2021) The PRISMA 2020 statement: an updated guideline for reporting systematic reviews. BMJ 372:n71

[8] Virhammar J, Laurell K, Cesarini KG, Larsson EM (2014) Preoperative prognostic value of MRI findings in 108 patients with idiopathic normal pressure hydrocephalus. AJNR Am J Neuroradiol 35:2311-2318. doi:10.3174/ajnr.A4046

[9] Yamada SM, Masahira N, Kawanishi Y, Fujimoto Y, Shimizu K (2013) Preoperative acetazolamide SPECT is useful for predicting outcome of shunt operation in idiopathic normal pressure hydrocephalus patients. Clin Nucl Med 38:671-676. doi:10.1097/RLU.0b013e31829959a9

[10] Ziegelitz D, Starck G, Kristiansen D, Jakobsson M, Hultenmo M, Mikkelsen IK, Hellström P, Tullberg M, Wikkelsø C (2014) Cerebral perfusion measured by dynamic susceptibility contrast MRI is reduced in patients with idiopathic normal pressure hydrocephalus. J Magn Reson Imaging 39:1533-1542. doi:10.1002/jmri.24292
